# Supplementary material for: How do children’s hospitals address health inequalities: a grey literature scoping review
Source: BMJ Open. 2024 Jan 3;14(1):e079744. doi: 10.1136/bmjopen-2023-079744 (PMC10773373; doi:10.1136/bmjopen-2023-079744)
Supplement: Supplementary data [file bmjopen-2023-079744supp002.pdf]

Table 1. Detailed summary of included approaches

In-text ref	Country	Hospital	Approach	Type	Details	Year	Inequity	Setting	Population	Outcomes reported	Staff/ resources involved	Other useful learning	Evaluation/ measure of success
35	England	Birmingham Women and Childrens Hospital	Intervention	Micro	Pilot project targeting families at risk of nonattendance - offered free transport to the hospital.	2023 Pilot	Social determinants of health	Hospital	Families in most deprived areas	90% of the study group took up the offer of help with travel and another 140 appointments that were reallocated.	Administration staff worked extra hours making phone calls, supported by Trust's interpreter team.	Staff also started to develop better relationships with the people they were contacting, helping to build trust and increase engagement with their local community.	Number of uptake, reduction in WNB.
36	England	Sheffield Children's Hospital	Intervention	Micro	Families Matter Project - aimed to provide parents with resources to allow them to maintain basic hygiene from the point of admission, ensure that no families go hungry by developing a food pledge and supporting families by signposting them to resources to support them financially alongside the development of access to Citizen's Advice booths.	2022	Social determinants of health	Hospital	All	Positive feedback from families on the provision of basic care resources, particularly sanitary products. Staff members have found that families are less stressed about how they will look after themselves when in patients, it has also been a positive for staff wellbeing in taking care of our families.	All staff	N/A	Qualitative feedback from parents.
37	Australi a	Sydney Children's Hospital Network	Strategy	Macro	Strategy with interventions as a result such as projects/ increasing number of Aboriginal staff, coordinator role for Aboriginal Health Outcomes and Equity Project (aiming to ensure the constant delivery of high-quality, equitable services to Aboriginal children).	2018	General	Hospital and community	Aboriginal children	Aboriginal workforce has grown by 20 staff in clinical and non-clinical roles. Established new dashboard to look at real time data and if aboriginal patients have been seen by an Aboriginal health worker within 24 hours of admission.	All staff withing the hospital network, the Aboriginal Health Outcomes and Equity Project Manager, Aboriginal Health Workers	"The advantage of addressing Aboriginal health as part of a children's hospital network is that we have a wide range of services and, therefore, an opportunity to intervene early in the life course."	Not reported.
38	Canada	Health Sciences Centre Children's hospital Winnipeg	Intervention	Micro	Employed a 'Jordan's Principal Coordinator' - The Jordan's Principle Coordinator at HSC Children's Hospital will assist First Nation children and youth under 18 and their families in accessing programs, services, and supports to improve health, education, and social outcomes.	2022	Access	Hospital	First nation children	None reported	Coordinator and team	N/A	Not reported.
39	England	Newcastle Upon Tyne Hospitals NHS Trust	Intervention	Micro	Health contact for school-aged children living in homeless accommodation (previously only offered to <5 year olds). Children and parents are offered an appointment for a health assessment on two days per month, initially for a three-month pilot period.	2022	Access	Community	Children living in homeless accommodation	In the three-month trial period, 20 school age children were offered health assessments. 17 out of those 20 have had the health assessments. Ten children were supported with oral hygiene needs, with four referrals into treatment at acute level, six had vision needs and four were referred to Ophthalmology for treatment. Five had incomplete immunisations, three received referrals to tuberculosis service, and two had an increased BMI.	Two public health school nurses	The service has used this model of service delivery to support health assessments for asylum seekers locally, and all children under 19 have received health assessments prior to being rehoused. Staff visit the asylum-seeking families' temporary accommodation once per week. Also plan to offer the same whole family support to families in a domestic abuse refuge.	Number of children offered health assessments and number of health interactions that resulted from those (e.g. referrals to a certain service, immunisations).
39	England	Gateshead Health NHS Foundation Trust	Service	Meso	Single point of access for refugee and asylum seekers. Undertake a health needs assessment and remove barriers by connecting families directly to the right health, statutory or voluntary services.	2022	Access	Community	Refugees and Asylum Seekers	Over 140 children had comprehensive health assessments in a secondary care clinic, and dozens of families had similar assessments in a local asylum seeker accommodation through primary care. "Health needs that would have been unrecognised and unmet have been addressed, improving health outcomes for these families." Based on these data, a project spanning primary and secondary care, linking a wide range of other services, has been commissioned to further improve capacity and quality of this work.	Not specified	Engagement of the various stakeholders is critical for this approach to be successful.	Numbers of appointments.

Table 1. Detailed summary of included approaches

In-text ref	Country	Hospital	Approach	Type	Details	Year	Inequity	Setting	Population	Outcomes reported	Staff/ resources involved	Other useful learning	Evaluation/ measure of success
40	Canada	Hospital for Sick Children Toronto (SickKids)	Strategy	Macro	EDI strategy and indigenous health council aims to make care more equitable for indigenous people, ultimately striving to improve child health outcomes.	2020	General	Hospital and community	All	None reported.	Patients, EDI Committee	N/A	Measurement and evaluation of disparities in access to care; research and education to minimize variation .
41	England	Alder Hey, Liverpool	Strategy	Macro	Innovation strategy and associated projects e.g. analysis of 'was not brought' (WNB) children data to identify families at risk of nonattendance. Other innovation e.g. transparent masks	2022 pilot	General	Hospital	All	WNB brought project- AI- ability to calculate the likelihood of whether a child will come to their appointment or not, and so make an early intervention: piloted on 34 patients in general paediatrics, model has 80% accuracy.	Innovation team	Planning to share WNB project AI tool with other Trusts.	Success of tools e.g. accuracy of AI predictor.
42	England	Sheffield Children's Hospital	Strategy	Macro	Strategy seeks to minimise the impact of health inequalities on mental and physical health every time a family interacts with us to improve experience, access, and outcomes. Working with partners to address the root causes of inequalities and invest where the need is greatest.	2022	General	Hospital and advocating wider	All	None reported.	Not specified	N/A	Not reported.
43	USA	Boston Children's Hospital	Declaration and goals	Macro	To address racial injustice and health disparities, Boston Children's released a Declaration on Equity, Diversity, and Inclusivity. The declaration established six goals to elevate health equity as an enterprise priority, core to everything the hospital does.	2020	General	Hospital	All	Six goals committing to addressing inclusion, recruitment, structural racism, employee education, leading on elimination of and metrics to track health disparities. No other outcomes reported.	Chief Equity and Inclusion Officer	N/A	Not reported.
44	New Zealand	Starship Hospital, Auckland	Ethos	Macro	The Starship Foundation is committed to meeting children and their whānau where they are, ensuring all children in New Zealand have access to world-class healthcare regardless of their ethnicity, where they live, or their family circumstance, decreasing ethnic health disparities in Māori Children, seeking to improve the health journey for tamariki Māori and their whānau, to ultimately achieve equitable outcomes.	2019	General	Hospital and community	All	Online content resonates strongly with Māori and Pacific audiences, with 667,006 post engagements reflecting strong connection and cut-through, Healthy Homes supported 492 households and fitted 237 homes with thermal curtains.	All staff, Starship Child Health has now embedded a Māori Health Team	Produce clinical guidelines for other hospitals.	Yearly impact reports.
45	England	Evelina	Intervention	Macro	NHS Rainbow Badges are being worn by staff. When staff sign up to wear the badge they are provided with information about the challenges people who identify as LGBT+ can face accessing healthcare and what they can do to support them. Wearing the badge is a sign that the wearer is someone patients can talk to about issues of sexuality and gender identity.	2019	Access	Hospital	Young LGBT+	Almost a third of staff have signed up to wear and be trained. This has since been rolled out across NHS Trusts in the UK and an associated toolkit developed. In 2021 the NHS Rainbow badge moved from a purely visual symbol to also incorporating an assessment and accreditation mode for NHS Trust.	All staff	Rolled out across other Trusts in England.	Uptake.
46	USA	Boston Children's Hospital	Established department / centre	Meso	Office of Health Equity and Inclusion. Coordinates equity, diversity and inclusion initiatives across the hospital.	2017	General	Hospital	All	Initiatives such as professional development seminars and training, career advancement and pipeline programs, mentoring, research, development of metrics.	Led by Chief Equity and Inclusion Officer	Work in partnership with Harvard Medical School and other partners.	Not reported.
47	USA	Boston Children's Hospital	Established research centre/ portfolio	Meso	Institute for Paediatric Health Equity: focuses on 3 areas: health equity research, inclusion, and public policy. Ensuring equitable health care access, delivery, and outcomes for all children.	2021	General	Hospital	All	Research projects to recruit diverse families, studies investigating missed appointments and culturally effective interventions. Seed grants to fund inequalities research.	Led by Senior Vice President. Also, Chief Equity and Inclusion Officer, Paediatric Radiologist	N/A	Not reported.
48	USA	Children's Hospital of Philadelphia (CHOP)	Established department / centre	Meso	Established Center for health equity: 4 core workstreams: Community Translation, Clinical Quality and Safety, Advocacy and Social Justice, and Research and Education.	2022	General	Hospital and community	All	Formation of a Neighbourhood Council.	Led by senior manager. Employ community consultant for each core area of focus and a village of community health workers. The Neighbourhood Council will consist of CHOP employees who live in the West Philadelphia area.	Has website and repository of resources for professionals.	Not reported.

Table 1. Detailed summary of included approaches

In-text ref	Country	Hospital	Approach	Type	Details	Year	Inequity	Setting	Population	Outcomes reported	Staff/ resources involved	Other useful learning	Evaluation/ measure of success
49	USA	Children's Hospital of Philadelphia (CHOP)	Established research centre/ portfolio	Meso	'Policy Lab': A research portfolio investigating how policies and programs can improve the health of historically marginalized children and adolescents. Advancing health equity through a research lens. PolicyLab is a Center of Emphasis within Children's Hospital of Philadelphia's Research Institute	2022	General	Online, hospital, community	Minority infants and mothers, LGBTQ children, immigrant families, low-income families	Research articles and multiple projects including a brachial plexus recruitment study. Before the health equity intervention, 54% of diverse families and 35% of white families agreed to participate in the study. After implementing the recommended strategies, 68% of diverse families and 71% of white families enrolled in the study.	Team includes 35 faculty and more than 60 staff who are experts in medicine, public health, social work, psychology, law, biostatistics, health services research and population health	Database/ repository of projects and publications.<br>Inclusive recruitment strategies help recruit patients into trials, so that the study population is more representative of the real population.<br>Simple modifications are required to completely change the outcome of recruitment – "it's simple things that make a difference."	Brachial plexus study - the % of diverse and % of white families that were invited to participate in the research that decided to take part (changes pre to post study).
50	USA	Cincinnati Children's	Established department / centre	Meso	The Center for Child Equity builds equitable care capabilities among staff through training, consultation and partnerships with research and clinical divisions, diversity and community relations teams, as well as a broad coalition of families and other external stakeholders.	2021	General	Hospital and community	All	None reported.	Hospital, community and social care partners	N/A	Not reported.
51	USA	Seattle Children's Hospital	Established department / centre	Meso	Center for Diversity and Health Equity (CDHE). Includes patient and family education programme, interpreter services, cultural navigation, family resource centre (kitchen, showers, computers, laundry, TV, Wi-Fi, parent support, phone chargers etc.), guest services, spiritual care, resources for transgender patients.<br>Health Equity Research Grants Program: to fund project to advance work around health equity, have Education and resources for healthcare professionals on equity, and a programme around human trafficking.	2021	General	Hospital	All	Programme of work around human trafficking including video workshops and video. Training for staff around health equity.	Diversity and Health Equity team: 14 people, work closely with the Odessa Brown Children's Clinic, Seattle Children's Research Institute and many other groups within Seattle Children's	Created a self-assessment toolkit for staff to assess performance towards health equity.	Not reported.
52	USA	Children's Hospital of Philadelphia (CHOP)	Partnership Initiative	Meso	To target the social determinants of health. Healthier Together partners with community groups, nonprofits and government agencies to learn from each other and multiply the impact of the work. Residents and community organisations participate in oversight/governance meetings, and outreach for program development. Advisers include experts from across CHOP, residents from West and Southwest Philadelphia, and representatives from the School District of Philadelphia, Philadelphia Department of Public Health and other services.	2019	Social determinants of health	Hospital and community	Children in three zip codes in West and Southwest Philadelphia	$10 million invested in the community. 13,450 individuals served with 21 partners and collaborators. $3,840 back to families on average through financial counselling and tax prep. 136 homes renovated to reduce asthma triggers. 45,570+ healthy meals distributed to families	Hospital and partners	Purchase goods and services from local companies that value a diverse workforce and provide meaningful work opportunities to underrepresented members of our community.<br>Healthier Together partners with community groups, nonprofits and government agencies to learn from each other and multiply the impact of the work. Residents and community organisations participate in oversight/governance meetings, and outreach for program development.	$ invested in community, individuals served, homes renovated, meals distributed.
53	Canada	Holland Bloorview Kids Rehabilitation Hospital, Toronto	Screening tool	Meso	Created a social needs screening tool, used during appointments by trained clinicians to identify the social needs and barriers of clients and their families. Family Navigation Hub was created: a resource that offers one-on-one consultations and services as well as monthly virtual workshops in partnership with external organizations, known as the CommunityCONNECT series, for families to access and to help close the gaps when it comes to housing, food, childcare, respite and community connection. Developed a partnership with FoodShare to look at food security needs.	2023	Social determinants of health	Hospital and community	Children with a disability	Community Kitchens Series from FoodShare Toronto: families who register are sent ingredients to prepare culturally diverse recipes. They prepare the meals together with other families while on Zoom, Housing support, social needs screening tool for clinicians.	Screened by clinicians, if eligible family support specialists work with families	The FoodShare program has brought families together and provided a sense of community.	Qualitative feedback from parents.

Table 1. Detailed summary of included approaches

In-text ref	Country	Hospital	Approach	Type	Details	Year	Inequity	Setting	Population	Outcomes reported	Staff/ resources involved	Other useful learning	Evaluation/ measure of success
54	Australia	Queensland Childrens Hospital, Brisbane	Service	Meso	Indigenous liaison service - to support, advocate, provide assistance to indigenous people accessing health and social services.	2020	Outcomes	Hospital	Children and families from Aboriginal and Torres Strait Islander communities	None reported.	Indigenous liaison officers	N/A	Not reported
55	USA	Boston Children's Hospital	Advocacy	Micro	Analysed data on impact of Covid-19 on communities of colour. Raised awareness on structural racism and differences in outcomes. Communication through Twitter.	2021	Outcomes	Online	Communities of colour	The posts reached more than 100,000 people, with reposts by hospitals and several state legislators.	Group of paediatricians	Analysing data showed that rates of Covid-19 infection, serious illness, and death were far higher in Black populations throughout the country. Recognized that data can play an important role in driving more equitable decisions and policies. Social media can be a useful medium to spread awareness, but it has its limitations as well.	Number of people reached on Twitter, number of reposts.
56	Canada	Hospital for Sick Children Toronto (SickKids)	Screening tool	Micro	Developed a paediatric data tool and health equity survey. Also provided training for staff.	2019	General	Hospital	All	Increased focus on equity. Better data collection. Staff trained.	Clerical staff collected data	Registration is an optimal point in the healthcare visit to collect sociodemographic information. Patients and caregivers willing to provide information.	Satisfaction of parents in giving information. Number and percentage of tools completed. Number of staff trained.
57	Canada	British Columbia Children's Hospital Vancouver	Service	Micro	Primary healthcare outreach clinics for children, youth, and families in Vancouver's inner-city neighbourhoods. the RICHER program seeks to address health equity disparities and particularly disparities in health care access that many marginalized and vulnerable children and families experience. The model recognizes the importance of trusted relationships and seeks to increase capacity and resilience for the families served.	2023	Access	Community	Inner city families	A wide range of community outreach services at a wide range of centres.	Nurse Practitioners, general paediatricians, development paediatricians, Adolescent Health and Medicine doctors, Child and Adolescent Psychiatry physicians, dermatology doctor, research manager	N/A	Not reported.
58	Australia	Sydney Children's Hospital Network	Intervention	Micro	Two new projects - the RuralKidsGPS project and PEACH project. Aim to improve equity and access to healthcare services. RuralKids will trial integrated care in rural districts and PEACH will establish technological solutions and sustainable systems for complex needs.	2021	Access	Community	Rural populations and socioeconomically disadvantaged communities	RuralKidsGPS: Paediatric care co-ordination has already shown promising results in metropolitan Sydney. Over a two-year period, it demonstrated a 40 per cent reduction in emergency department presentations and a 42 per cent decrease in day-only admissions – saving more than 50,000km of travel for families.	Care coordinators will work closely with local care teams and families to create shared care plans, support technology-enabled healthcare, and connect families with local services to meet the child's needs.	N/A	The success of the RuralKidsGPS and PEACH will be evaluated in the next few years with the intention to roll out both programs more widely across the state.
59	USA	Boston Children's Hospital	Intervention	Micro	Case management and home visits by a community health worker for children from low-income families of colour with asthma.	2023	Outcomes	Community	Children with asthma from low-income families of colour	By 2022, there were more than 2,491 patients who enrolled in the Community Asthma Initiative. Their involvement led to: 82% reduction in the percentage of patients with asthma-related hospitalization, 55% reduction in the percentage of patients with Emergency Department visits, 45% reduction in the percentage of patients with missed school days for children, 55% reduction in the percentage of patients with lost work days for parents.	Community health workers	Beneficial to wider health of children involved.	Changes in the percentage (%) of patients with asthma-related hospitalization, % of patients with Emergency Department visits, % of patients with missed school days for children, % of patients with lost workdays for parents.
