# Supplementary material for: How do children’s hospitals address health inequalities: a grey literature scoping review
Source: BMJ Open. 2024 Jan 3;14(1):e079744. doi: 10.1136/bmjopen-2023-079744 (PMC10773373; doi:10.1136/bmjopen-2023-079744)
Supplement: Supplementary data [file bmjopen-2023-079744supp001.pdf]

Appendix I: Search terms

Database	Search	Terms
Overton	1	(child OR children OR paediatric OR pediatric OR "young people") AND (hospital) AND ("health inequality" OR "health disparity" OR "health equity")
	2	title: equity child
	3	title: inequality child
	4	title: children hospital equity
	5	title: addressing inequalities
	6	title: addressing inequity
Open Grey	1	Health inequality
	2	Health equity
	3	Health Disparity
Openmd.com	1	(child OR children OR paediatric OR pediatric OR "young people") AND (hospital) AND ("health inequality" OR "health disparity" OR "health equity")
Tripdatabase	1	title: inequalities children hospital
	2	title: address health equity
	3	addressing child health inequality hospital
Duckduckgo	1	hospital based approaches to addressing health inequalities
	2	how do childrens hospitals reduce inequalities
	3	(child OR children OR paediatric OR pediatric OR "young people") AND (hospital) AND ("health inequality" OR "health disparity" OR "health equity")
Google	1	(child OR children OR paediatric OR pediatric OR "young people") AND (hospital) AND ("health inequality" OR "health disparity" OR "health equity")
	2	how do childrens hospitals reduce inequalities
	3	childrens hospital approach to inequalities
	4	Childrens hospital strategy to reduce health inequalities
APHA	1	Childrens hospital
	2	child health inequality
	3	child equity
	4	child health disparity
WHO	1	children's hospital
	2	health inequality
	3	child health equity
	4	health equity
	5	health disparity
NHS England	1	childrens hospital
	2	health inequality
	3	health equity
	4	health disparity
	5	health inequality
OHID	1	"children's hospital"
	2	"child health inequalities"
	3	"health inequalities" AND "hospital"
HMIC	1	“inequalities”
Hospital websites	All	Inequal* OR inequity OR disparit*

Appendix II: Data extraction form

**Study details and characteristics**	
Reference/ weblink	
Hospital name	
Country of study	
Year published/ website updated	
Population and sample size	
Context (healthcare system)	
Publication type (e.g. report/ website/ news article	
Stand alone hospital / part of network	
**Details of the approach**	
Type of approach (e.g. strategy/ centre..)	
Aim of the approach	
Inequality in focus	
Population in focus	
Setting	
Length of intervention?	
Staff	
**Results extracted from study** (in relation to the concept of the scoping review)	
Main Outcomes/ Results	
Additional outcomes/ any differential effects	
Key findings/ Conclusions	
How were effects measured?	
Other notes	
