# Supplementary material for: How do children’s hospitals address health inequalities: a grey literature scoping review
Source: BMJ Open. 2024 Jan 3;14(1):e079744. doi: 10.1136/bmjopen-2023-079744 (PMC10773373; doi:10.1136/bmjopen-2023-079744)
Supplement: Author's manuscript [file bmjopen-2023-079744.draft_revisions.pdf]

BMJ Open

BMJ Open is committed to open peer review. As part of this commitment we make the peer review history of every article we publish publicly available.

When an article is published we post the peer reviewers' comments and the authors' responses online. We also post the versions of the paper that were used during peer review. These are the versions that the peer review comments apply to.

The versions of the paper that follow are the versions that were submitted during the peer review process. They are not the versions of record or the final published versions. They should not be cited or distributed as the published version of this manuscript.

BMJ Open is an open access journal and the full, final, typeset and author-corrected version of record of the manuscript is available on our site with no access controls, subscription charges or pay-per-view fees (http://bmjopen.bmj.com).

If you have any questions on BMJ Open's open peer review process please email info.bmjopen@bmj.com

BMJ Open

How do children’s hospitals address health inequalities: a grey literature scoping review

Journal:	*BMJ Open*
Manuscript ID	bmjopen-2023-079744
Article Type:	Original research
Date Submitted by the Author:	10-Sep-2023

Complete List of Authors:	Brennan, Louise; Lancaster University, Medical School; St Helens and Knowsley Teaching Hospitals NHS Trust<br>Broad, Eleanor; Dumfries and Galloway Royal Infirmary<br>Stres, Dora Pestotnik; Lancaster University<br>Egboko, Fiona; Lancaster University<br>Patel, Pallavi; Lancaster University; St Helens and Knowsley Teaching Hospitals NHS Trust,<br>Brewster, Liz; Lancaster University, Lancaster Medical School<br>Lunn, Judith; Lancaster University<br>Isba, Rachel; Lancaster University; Alder Hey Children's NHS Foundation Trust

Keywords:	Health Equity, PAEDIATRICS, PUBLIC HEALTH

SCHOLARONE™ Manuscripts

I, the Submitting Author has the right to grant and does grant on behalf of all authors of the Work (as defined in the below author licence), an exclusive licence and/or a non-exclusive licence for contributions from authors who are: i) UK Crown employees; ii) where BMJ has agreed a CC-BY licence shall apply, and/or iii) in accordance with the terms applicable for US Federal Government officers or employees acting as part of their official duties; on a worldwide, perpetual, irrevocable, royalty-free basis to BMJ Publishing Group Ltd ("BMJ") its licensees and where the relevant Journal is co-owned by BMJ to the co-owners of the Journal, to publish the Work in this journal and any other BMJ products and to exploit all rights, as set out in our licence.

The Submitting Author accepts and understands that any supply made under these terms is made by BMJ to the Submitting Author unless you are acting as an employee on behalf of your employer or a postgraduate student of an affiliated institution which is paying any applicable article publishing charge ("APC") for Open Access articles. Where the Submitting Author wishes to make the Work available on an Open Access basis (and intends to pay the relevant APC), the terms of reuse of such Open Access shall be governed by a Creative Commons licence – details of these licences and which Creative Commons licence will apply to this Work are set out in our licence referred to above.

Other than as permitted in any relevant BMJ Author's Self Archiving Policies, I confirm this Work has not been accepted for publication elsewhere, is not being considered for publication elsewhere and does not duplicate material already published. I confirm all authors consent to publication of this Work and authorise the granting of this licence.

For peer review only

How do children's hospitals address health inequalities: a grey literature scoping review

Louise Brennan [1, 2] l.brennan2@lancaster.ac.uk

Dora Pestotnik Stres [1] d.pestotnikstres@lancaster.ac.uk

Fiona Egboko [1] f.egboko@lancaster.ac.uk

Pallavi Patel [1,2] pallavi.patel4@nhs.net

Eleanor Broad [3] eleanor.broad@doctors.org.uk

Liz Brewster [1] e.brewster@lancaster.ac.uk

Judith Lunn [1] j.lunn1@lancaster.ac.uk

Rachel Isba [1,4] rachel.isba@lancaster.ac.uk (corresponding author)

[1] Lancaster Medical School, Lancaster University, Lancaster, UK

[2] St Helens and Knowsley Teaching Hospitals NHS Trust, Prescot, UK

[3] Dumfries and Galloway Royal Infirmary, Cargenbridge, Dumfries, UK

[4] Alder Hey Children's NHS Foundation Trust, Liverpool, UK

For peer review only

For peer review only - http://bmjopen.bmj.com/site/about/guidelines.xhtml

ABSTRACT (280)

Introduction:

Health inequalities are systematic differences in health between people, that are avoidable and unfair. Health inequalities can be measured by life expectancy, health outcomes and access to care. Globally, more political strategies are required to address health inequalities, which have increased since the global SARS-CoV-2/COVID-19 pandemic, with a disproportionate impact on children.

Aim:

This scoping review aimed to identify and collate information on how hospitals around the world that deliver care to children have addressed health inequalities.

Methods:

This scoping review focussed solely on grey literature due to the appropriateness of that approach for complex, multistakeholder interventions that lack robust data and predictability. Following Joanna Briggs Institute guidelines, a four-step approach to identifying literature was adopted. Retrieved items were screened against clear inclusion and exclusion criteria before data were extracted by two independent reviewers using a data extraction tool.

Results:

Our study identified 26 approaches to reduction of health inequalities, from 17 children's hospitals. Approaches were categorised based on their size and scope. Seven approaches were defined as macro, including hospital-wide inequality strategies. Ten approaches were classed as meso, including establishment of new departments and research centres. Micro approaches (n=9) included one-off projects or interventions offered to specific groups/ services. Almost half of the reported approaches did not discuss evaluation of impact.

Conclusions:

Children's hospitals provide a suitable location to conduct public health interventions. This scoping review provides examples of approaches on three scales delivered at hospitals across high-income countries. Hospitals with the most comprehensive and extensive range of approaches employed dedicated staff within the hospital and community. This review indicates the value of recruitment of both public health-trained staff as well as culturally-similar staff to deliver community-based interventions.

For peer review only

For peer review only - http://bmjopen.bmj.com/site/about/guidelines.xhtml

Strengths and limitations:

- Unlike traditional scoping reviews of peer-reviewed literature, this review focusses solely on grey literature.
- An extensive search of online grey literature databases, targeted websites and children's hospitals webpages was conducted.
- Grey literature may be more appropriate for areas of rapidly changing research such as new approaches to addressing health inequalities.
- Items were only included if available in English or translatable through online apps.

Key words:

Health equity, paediatrics, public health

Word Count: 3,833

For peer review only

For peer review only - http://bmjopen.bmj.com/site/about/guidelines.xhtml

INTRODUCTION

Health inequalities are systematic differences in health between people, that are avoidable and unfair[1]. Whilst most-commonly researched in relation to socioeconomic deprivation [2 3 4 5], the links between health inequalities and other types of social inequality such as disability [6], race [7], religious beliefs [8] and sexual orientation [9] are also well-evidenced.

Health inequalities are evident between and within countries across the world. Two examples of this are life expectancy and under-5s mortality rates. In sub-Saharan Africa, children are 14 times more likely to die before the age of 5 than in the rest of the world [10]. In India, the under-5 mortality rate was 106 per 1000 births to mothers with no education, compared with 49 per 1000 births to mothers with secondary or higher education [11]. In the UK, life expectancy is also socially patterned, with, for example, people residing in the most deprived areas of England living an average of 14 years less than those in the least deprived areas [12].

Disparities in health outcomes are also evident globally. Research conducted in the USA found that rates of low birthweight were three times higher amongst babies born to Black mothers with no college education, compared with babies born to white mothers with a college education [13]. In the UK, children living in deprived areas are more likely to suffer from conditions such as tooth decay, asthma, diabetes and obesity [14].

However, health inequalities are not just measured in life expectancy and health outcomes; patterns in accessing care are also correlated to socioeconomic status. In New Zealand, Māori young people are the least likely to be enrolled with a Primary Health Care provider [15]. In the UK, children from the most deprived areas are less likely to be brought to outpatient appointments [16], but more likely to attend the Emergency Department, than those living in the least deprived areas [17 18].

Whilst the World Health Organization (WHO) has promoted the reduction of health inequalities as a global priority since its inaugural World Health Report in 1995 [19], political strategies to address the social determinants of health are still required in both high-, middle- and low-income countries [20]. In the UK, the National Health Service (NHS; a government-funded, free at the point of access, universal medical and healthcare service) has a legal duty to address health inequalities [21], and this was made a key commitment in the *NHS Long Term Plan* [22]. However, the SARS-CoV-2/COVID-19 pandemic has widened health inequalities [23] and critics have argued that the absence of a national health inequalities strategy has led to local healthcare systems – including hospitals – struggling to develop their own approach to addressing health inequalities [24] .

Recognising the disproportionately negative impact of the pandemic on children [25, 26], and lack of central steer on health inequalities in children and young people (CYP) in the UK [2728] and other countries [29], this scoping review aimed to identify and collate information on how hospitals around the world that deliver care to children have addressed health inequalities. The findings of this scoping review are intended to be used to inform children's hospitals on approaches, outcomes and experiences based on previous work, to influence future interventions and policy.

Review Questions

1. What approaches do hospitals take to address health inequalities in children and young people?

2. What health inequalities do the approaches focus on?

3. How is effectiveness measured and demonstrated?

For peer review only - http://bmjopen.bmj.com/site/about/guidelines.xhtml

METHODS

As presented in the published protocol [30], this scoping review focussed solely on grey literature due to the appropriateness of that approach for complex, multistakeholder interventions that lack robust data and predictability [31]. Grey literature is also more appropriate for topics that are under current review and moving at a fast pace, as is the case with interventions to address health inequalities. Furthermore, as hospitals and clinicians often have less capacity to publish academic literature in peer-reviewed journals, this methodology was deemed more appropriate as it allowed for a deeper, more systematic search of the internet and hospital websites.

Inclusion and exclusion criteria

As detailed in the protocol, items were included if initiated by a hospital, published in English, post-2010, focussed on children and with a clear aim to address health inequalities. Items were excluded if they did not explicitly state reducing health inequalities in their aim/ focus, and if the item did not describe an approach. Whole hospital approaches (including adults) and antenatal and prenatal services were also excluded.

Search Strategy

During January 2023, the online databases Overton, Open Grey, Openmd and tripdatabase were searched using the search terms outlined in the protocol. Secondly, search engines duckduckgo (allowing searches unaffected by previous search histories) and Google (using the anonymised search function) were searched using the search terms detailed in Appendix 1. The first 10 pages of returns were screened, unless items appeared to have continuing relevancy past 10 pages. Thirdly, targeted websites of the American Public Health Association (APHA), WHO, NHS England, the UK's Office for Health Improvement and Disparities (OHID) and the Health Management Information Consortium (HMIC) were explored using their search functions.

Fourth, as an add-on to the original search strategy, a list of worldwide children's hospitals was identified through an online encyclopaedia [32]. The authors searched the full list of children's hospital's websites individually, using individual website search bars and key terms. Non-English websites were translated using the Google Translate function.

Finally, snowballing was conducted at all stages of the search, where additional resources are identified through citations or weblinks [33].

The full search strategy and terms is detailed in Appendix 1.

Study selection and data extraction

Searches and results were managed using Microsoft Excel. All items were screened based on their title (and where necessary associated text). Items deemed relevant were recorded by saving their website link. All items retrieved in this title screening stage were then screened in full against the inclusion and exclusion criteria by two independent reviewers. Any uncertainties or disagreements were first discussed between the two reviewers and then resolved by a third reviewer (RI). Data extraction was carried out by two separate reviewers, using a data extraction form developed using JBI guidelines [34] (Appendix 2). Where more than one approach was identified from the same hospital, they were classed as separate (and classed as a unique item) if they had separate aims. In some cases, multiple records were identified from one hospital, however they were all relating to the same aim/ approach (e.g. items under one strategy), and in these cases, records were combined into one entry.

For peer review only - http://bmjopen.bmj.com/site/about/guidelines.xhtml

Data synthesis

Following data extraction, studies were tabulated by hospital. Key details of the approach and its associated resources, setting and other key details were also presented alongside any outcome data. Due to the varied nature of studies and approaches no meta-analysis was conducted. Quality assessment was not undertaken due to the grey literature focus and lack of reporting consistency.

Patients and public involvement

No patients or public were involved.

RESULTS

Search results

In total, 2,752 results were returned from our database searches (identification stage). Of these, 2,585 were deemed inappropriate for inclusion, leaving 167 records for full text screening against the inclusion and exclusion criteria. During full text screening a further 18 records were identified through snowballing. Forty-five records were identified as duplicates and excluded. Items were then excluded if they did not meet the inclusion criteria (n=108). Six records were excluded after discussion with a third independent reviewer as consensus could not be met between the initial two reviewers, leading to 115 exclusions in total. Data were extracted from 26 texts (Figure 1).

General characteristics of studies

Articles included in the results were published online between 2017 and the search date in January 2023. The approaches were from five high-income countries (United States of America, Australia, Canada, UK and New Zealand). Seventeen separate hospitals were included in the results. Boston Children's Hospital initiated five of the included approaches, Children's Hospital of Philadelphia (CHOP) initiated three separate approaches, with The Hospital for Sick Children Toronto (SickKids) and Sheffield Children's Hospital each initiating two; the remainder of hospitals had one record each. A summary of the included approaches is presented in Table 1. Whilst our protocol intended to investigate all hospitals providing care to children our results originated solely from children's hospitals.

Categorisation of approaches

Approaches were categorised into three themes based on the size and reach of the approach they were describing and are detailed below. 'Macro' approaches were organisation-wide, impacting the activity of the whole children's hospital, e.g. a formally-stated strategy. The approaches were classified as 'meso' if they involved setting up a new department or would change the activity of more than one department or service. 'Micro' approaches were single interventions, or projects that were short term or small in scale, e.g. reducing missed outpatient appointments. Almost half of the total 26 approaches (n=12) aimed to address a broad range of inequalities for the whole paediatric population. Meso or micro level approaches were more likely to focus on specific inequalities (e.g. financial hardship [35 36]) or inequalities in access or outcomes for specific groups (e.g. Aboriginal children [37] First Nation Children [38] or refugees and asylum seekers [39]).

Macro

Seven approaches were categorised as macro. Four of these were hospital-wide strategies, specifically aimed to address: health equity (Australia) [37]; equity, diversity and inclusion (EDI)

For peer review only - http://bmjopen.bmj.com/site/about/guidelines.xhtml

(Canada) [40]; health inequalities within an innovation strategy (UK) [41]; and inclusion of health inequalities in a clinical strategy (UK) [42].

Two hospitals had an 'ethos of equity' – in Boston this was formalised through a declaration and goals [43]. At the Starship Hospital in New Zealand this was not formalised in a strategy but is conceptualised as a commitment in all that they do [44].

The final macro approach to health inequalities began as a micro approach but has since become hospital-wide and replicated across the UK. The *rainbow badge initiative* is for staff, who, when signing up to wear a rainbow badge receive information and training about the challenges that people who identify as LGBT+ can face when accessing healthcare and what they can do to support them [45].

Meso

Ten approaches were characterised as meso. Four of the US hospitals had established a centre aiming to address equity with dedicated staff. In Boston, the *Office for Health Equity and Inclusion*, aimed to coordinate EDI initiatives across the whole hospital [46] and the *Fenwick Institute*, focused on research into paediatric health equity [47]. The latter has provided funding for inequalities research and delivered research projects, including investigation into missed appointments and effective interventions to recruit diverse families. CHOP also established a *Center for Health Equity* [48] and a research *Policy Lab*, which aimed to advance health equity through a research lens [49]. The Cincinnati Children's Hospital's *Center for Child Equity* aimed to build equitable care capabilities in staff [50], and Seattle Children's Hospital's *Center for Diversity and Health Equity* focused on patient and family education programmes [51]. These latter hospitals have no separate research centre but include research in their equity approach.

Alongside their centre, CHOP had also established the *Healthier Together* partnership, together with community groups and non-profit government agencies to invest money into the community, families and individuals. Interventions included distribution of healthy meals, home renovation and financial counselling [52]. In Toronto, Canada, the *Family Navigation Hub* was established following the development of a social needs screening tool [53]. The hub aimed to help families to access and close the gaps around availability of housing, food, childcare, respite and community connection [49].

Several meso level approaches focused on specific patient groups. An *Indigenous Liaison Service,* established in Queensland Children's Hospital, Australia, targeted support, advocacy and provided assistance to indigenous people accessing health and social services [54]. Using the nationally-established *Jordan's Principle*, which ensures that First Nations children living in Canada can access services when/ as they are needed, the Health Sciences Centre Children's Hospital (Winnipeg) employed a *Jordan's Principle* co-ordinator to assist First Nations children and young people to access care at their facility [38]. Similarly, in Gateshead, England, a service had been developed to provide a single point of access for refugee and asylum-seeking children and families. This aimed to reduce barriers to healthcare by connecting families to the right health, statutory or voluntary service leading to referrals for oral health, obesity, immunisations and the tuberculosis (TB) service [39].

Micro

Nine approaches were categorised as 'micro' meaning they were projects or interventions used across one service/ department rather than at a larger scale. Three projects originated in England. The *Families Matter* project in Sheffield aimed to help parents with financial cost of staying in

For peer review only

hospital, and provided resources to allow them to maintain basic hygiene and needs, including sanitary products, food and additional support such as signposting to additional providers of financial advice. At the time of data extraction, the hospital was piloting a free breakfast for all parents staying with their child. Feedback from staff and families was positive, with staff reporting that caregivers appeared less stressed as a result of the enhanced offers of support [36]. In Birmingham, a pilot project was running to offer free transport to families at risk of non-attendance at the hospital. Pilot data showed that 90% of the study group took up the offer of help and 140 appointments were relocated during the eight weeks [35]. Thirdly, Newcastle upon Tyne Hospitals NHS Trust trialled assigning a health contact, who conducted health assessments for school-aged children living in homeless accommodation. In the three-month pilot, 17 of the 20 children assigned a health contact had health assessments, leading to support with oral health, ophthalmology treatment, referrals for immunisations and access to the TB service [39].

Data collection and analysis was the focus of two projects that fell into the micro approach category. During the SARS-CoV-2/COVID-19 pandemic, Boston Children's Hospital analysed data on the impact of the pandemic on communities of colour and published the findings through Twitter. The posts reached more than 100,000 people, raising awareness and reaching state legislators [55]. SickKids, Canada developed a paediatric data tool and health equity survey as part of a data collection pilot. Clerical staff collected data and 3,000 staff received training on culturally-competent care. Parents and carers reported satisfaction with the data tool and registration was identified as the optimal point in the healthcare visit to collect sociodemographic data [56].

Community outreach projects were common in the approaches we found. In Canada, led by British Columbia Children's Hospital, the *RICHER* (Responsive Intersectoral Child and Community Health Education and Research) programme was designed to address disparities in access to primary care for children, youth and families in Vancouver's inner cities [57]. A project run by Sydney Children's Hospital aimed to improve access and equity for children with complex needs using technology and those living in rural districts. They demonstrated a 40% reduction in Emergency Department visits and a 42% decrease in day admissions, saving more than 50,000km travel for families [58]. In Boston, children from low-income families of colour, with asthma, were the target population for case management and home visits by a community health worker. An 82% reduction in asthma-related hospitalisation rates was reported in the 2,491 enrolled patients. Other outcomes included 55% reduction in lost workdays for parents and 45% reduction in missed school days [59].

Evaluation tools

Nearly half (n=12) of the approaches reported no evaluation/ measure of success in their communication. Those hospitals reporting their outcomes used a range of different measures, with uptake/ number of people involved being the most common (n=10). Several approaches used caregiver satisfaction (n=3), with qualitative findings providing powerful quotes from service users [53]. Other less common measures were number of staff trained (n=2), accuracy of prediction tool (n=1), and money invested in the wider community by the hospital (n=1). Those hospitals that attempted to quantify their impact on health inequalities reported outcomes such as homes renovated [52] or modified [44], change in research study uptake in diverse families [49], reductions in hospitalisation, missed days from work and lost days at school [59], reduction in emergency admissions and miles travelled [58].

Staffing/ resources

For peer review only - http://bmjopen.bmj.com/site/about/guidelines.xhtml

Approaches varied in their approaches to staffing. Shorter-term projects used existing staff such as clerical staff for data collection [56 35], whereas new centres/ dedicated departments reported new staff structures with dedicated teams and additional infrastructure. For community outreach or targeted interventions with specific communities, positive outcomes were attributed to culturally-similar staffing [44 37 54 38]. Taking the service out into the community was also reported to improve outcomes for traditionally-marginalised patients, and in these cases, community health workers were often utilised. Approaches did not specify the cost of establishing and delivering their approach and therefore data could not be compared.

DISCUSSION

In this paper we have provided a synthesis of the approaches taken by children's hospitals in addressing health inequalities experienced by children and young people. Our search of grey literature aimed to be a comprehensive way to investigate unpublished literature produced by hospitals and their partners. Whilst our search aimed to investigate all hospitals providing care for children, the results originated solely from children's hospitals.

Our study identified 26 approaches to reduction of health inequalities, from 16 children's hospitals, all in high-income countries. Approaches have been categorised according to their depth and breadth of reach, from macro whole-organisation approaches, down to small micro pilot projects. The majority of approaches were categorised as meso and were commonly newly-established departments or centres dedicated to addressing equity and research into this area.

Twelve approaches had no specific inequality as their focus, instead taking a broad approach to addressing equity. Seven hospitals addressed inequalities in access to healthcare, a further four focussed on social determinants of health and the remaining three addressed health outcomes for specific groups.

Almost half of the approaches failed to report outcomes or their methods of evaluation/ assessment and very few reported costs. Although it is notoriously difficult to demonstrate the impact of public health interventions [60], there is still a recognised need to build an evidence base around interventions that aim reduce health inequalities and improve population health [61]. It is therefore important for hospitals to better evaluate and communicate their activities and findings in the future.

Few publications relating to hospital approaches to address health inequalities in the academic literature led to the refocus of this scoping review to grey literature only. Previous research has suggested that lack of time is the most common reason for non-publication of medical and health related studies [62]; however, this scoping review highlights the merits of publishing information on websites and other non-peer reviewed repositories of information, whilst also supporting the utilisation of grey literature for reviews of hospital-based interventions.

Hospitals that did evaluate their approaches varied in their evaluation methods. At the most basic level, hospitals counted numbers involved and uptake of interventions. More complex evaluations looked at changes in missed appointments and emergency admissions by demographics such as ethnicity. Other hospitals considered missed school days and days from work for parents and carers. As this is a scoping review, an assessment of study quality was not undertaken, and findings should therefore be interpreted with care.

Hospitals with dedicated centres and a team of staff reported a greater number of more comprehensive outcomes. Hospitals with a dedicated health inequalities focussed strategy, or health

For peer review only - http://bmjopen.bmj.com/site/about/guidelines.xhtml

inequalities within their wider strategies were also more likely to have an increased level of activity. However, strategy did not always equate to outcomes, and it is therefore important for hospitals to consolidate strategy with projects and appropriate staff resources. Projects involving culturally-similar staff or dedicated community teams reported success in reaching historically marginalised communities.

There are several reasons for the possible exclusion of approaches from middle- and low-income countries. Firstly, due to time and resource constraints our search was limited to materials available in the English language. Translate apps were utilised where possible as all searching was completed using the internet, nevertheless this possibly restricted findings from some sites where translation was not accurate enough to identify alternative terms for our keywords. Secondly, it is highly probable that the terminology used to describe health inequalities e.g. disparities and inequities, are not universally adopted around the world, and approaches that were relevant were excluded due to the way the intervention was described. This was confirmed during screening of worldwide children's hospitals websites, where several potentially relevant approaches aimed to provide culturally-appropriate care rather than equity in health, and were therefore excluded. Hence, lack of reporting on approaches does not imply lack of activity in this review. Thirdly the exclusion of hospital-wide, antenatal, prenatal and adult focussed approaches may have led to the exclusion of early years and some family-centred approaches to addressing health inequalities.

The context of healthcare systems is important to consider when interpreting these results. The majority of approaches originated from US hospitals, where healthcare is not universal and hospital activity could be driven by factors different to those where healthcare is free at the point of access. Furthermore, since the Affordable Care Act of 2010, American non-profit hospitals are mandated to undertake a community needs assessment and documentation of how they are addressing needs every three years [63]. Similarly, in England there has been a national drive to address health inequalities due to their inclusion in the NHS Long Term Plan [22] and the current 'cost of living crisis' has impacted access to care [64] and fuelled activity to address the cost of hospital appointments for patients.

CONCLUSIONS AND RECOMMENDATIONS

Children's hospitals and hospitals that care for children provide a suitable location to conduct public health interventions. This scoping review has explored and summarised the approaches that some children's hospitals in high-income countries have taken to address health inequalities. Several children's hospitals (mainly in the US) have advanced equity programmes of work, but this may be driven by the regulatory environment.

There was a lack of robust evaluation of the approaches, despite public health interventions requiring appropriate evaluation to enable evidence-based practice.

The lack of published literature on hospital-led approached to health inequalities highlights the need for hospitals to promote the work that they are doing, so areas of good practice and learning can be shared and implemented. As demonstrated here, this could be via hospital websites or through networks. The dearth of peer-reviewed articles also highlights the relevance of grey literature for hospital-based research such as this. The development of further methodological guidance on grey literature-focused scoping and systematic reviews is warranted.

The findings of this review originated from a small number of high-income countries. It is possible that relevant approaches are being used elsewhere but not being published, and/ or are not labelled

For peer review only - http://bmjopen.bmj.com/site/about/guidelines.xhtml

as health inequalities. Further research into this would be warranted to complete a broader summary of approaches.

Hospitals with the most comprehensive and extensive range of approaches employ dedicated staff within the hospital and community. This review therefore provides evidence for the value of the recruitment of both public health trained staff as well as culturally-similar staff to deliver community-based interventions.

Conflicts of interests

The authors declare that they have no known competing financial interests or personal relationships that could have appeared to influence the work reported in this paper.

Author statement

Brennan, Isba, Brewster and Lunn conceptualised the work. Brennan, Pestotnik Stres, Egboko, Patel and Broad were responsible for running searches and screening retrieved items. Brennan and Pestotnik Stres independently extracted data. Discrepancies were discussed with Isba. Brennan prepared the original draft. Isba and Brewster critically reviewed and edited the work. All authors edited and agreed the final version and are accountable for the accuracy and integrity of the work.

Acknowledgements

Thank you to Louise Speakman, Lancaster University who kindly advised on the original search strategy.

Funding Statement

This work was supported by the National Paediatric Accelerator Programme, now the Children's Hospital Alliance, contracted by Sheffield Children's Hospital, grant number SCH5628.

For peer review only

References

1 Williams E, Buck D, Babalola G, et al. What are health inequalities?. *The King's Fund* 2022.

2 Marmot M. Fair society, healthy lives. *Fair society, healthy lives* 2013:1-74.

3 Graham H. Unequal lives: McGraw-Hill Education (UK) 2007.

4 Acheson D. Independent Inquiry into Inequalities in Health. *Science* 1998;10:217-33.

5 Patel JA, Nielsen F, Badiani AA, et al. Poverty, inequality and COVID-19: the forgotten vulnerable. *Public Health* 2020;183:110.

6 Emerson E, Baines S, Allerton L, et al. Health inequalities and people with learning disabilities in the UK 2011.

7 Elwell-Sutton T, Deeny S, Stafford M. Emerging findings on the impact of COVID-19 on black and minority ethnic people. *The Health Foundation* 2020.

8 Office for National Statistics. Religion and Health in England and Wales. 2020; Available at: https://www.ons.gov.uk/peoplepopulationandcommunity/culturalidentity/religion/articles/religionandhealthinenglandandwales/february2020. Accessed 30/06/2023.

9 Booker CL, Rieger G, Unger JB. Sexual orientation health inequality: evidence from understanding society, the UK longitudinal household study. *Prev Med* 2017;101:126-32.

10 World Health Organization. Health Inequalities and their causes. 2018; Available at: https://www.who.int/news-room/facts-in-pictures/detail/health-inequities-and-their-causes. Accessed 30/06/2023.

11 Balarajan Y, Selvaraj S, Subramanian SV. Health care and equity in India. *The Lancet* 2011;377:505-15.

12 Rashid T, Bennett JE, Paciorek CJ, et al. Life expectancy and risk of death in 6791 communities in England from 2002 to 2019: high-resolution spatiotemporal analysis of civil registration data. *The Lancet Public Health* 2021;6:e805-16.

13 Pollock EA, Gennuso KP, Givens ML, et al. Trends in infants born at low birthweight and disparities by maternal race and education from 2003 to 2018 in the United States. *BMC Public Health* 2021;21:1117.

14 Wickham S, Anwar E, Barr B, et al. Poverty and child health in the UK: using evidence for action. *Arch Dis Child* 2016;101:759-66.

15 Irurzun-Lopez M, Jeffreys M, Cumming J. The enrolment gap: who is not enrolling with primary health organizations in Aotearoa New Zealand and what are the implications? An exploration of 2015–2019 administrative data. *International journal for equity in health* 2021;20:1-11.

16 French LR, Turner KM, Morley H, et al. Characteristics of children who do not attend their hospital appointments, and GPs' response: a mixed methods study in primary and secondary care. *British Journal of General Practice* 2017;67:e483-9.

For peer review only - http://bmjopen.bmj.com/site/about/guidelines.xhtml

17 Jarvis S, Livingston J, Childs A, et al. Outpatient appointment non-attendance and unplanned health care for children and young people with neurological conditions: a retrospective cohort study. *Developmental Medicine & Child Neurology* 2019;61:840-6.

18 Scantlebury R, Rowlands G, Durbaba S, et al. Socioeconomic deprivation and accident and emergency attendances: cross-sectional analysis of general practices in England. *British Journal of General Practice* 2015;65:e649-54.

19 King NB, Harper S, Young ME. Who cares about health inequalities? Cross-country evidence from the World Health Survey. *Health Policy Plan* 2013;28:558-71.

20 Rebouças P, Falcão IR, Barreto ML. Social inequalities and their impact on children's health: a current and global perspective. *J Pediatr* 2022;98:55-65.

21 Equality Act. Equality Act 2010.

22 NHS. The NHS long term plan. *NHS* 2019.

23 Buck D, Lewis T. NHS England and tackling inequalities: times are changing. *The Kings Fund* 2021.

24 Ford J, Sowden S, Olivera J, et al. Transforming health systems to reduce health inequalities. *Future Healthcare Journal* 2021;8:e204.

25 McLellan A, Abbasi K. The NHS is not living with covid, it's dying from it. *BMJ* 2022;378.

26 Pickett K, Taylor-Robinson D, Erlam J. Child of the North: Building a fairer future after COVID-19 2021.

27 Bradley SH. The ethics and politics of addressing health inequalities. *Clinical Medicine* 2021;21:147.

28 Choudry M. Tackling health inequalities and promoting prevention: If not now, then when?. *Future Healthcare Journal* 2021;8:4.

29 Dickman SL, Himmelstein DU, Woolhandler S. Inequality and the health-care system in the USA. *The Lancet* 2017;389:1431-41.

30 Brennan L, Brewster L, Lunn J, et al. How do hospitals address health inequalities experienced by children and young people: a grey literature scoping review protocol. *BMJ open* 2023;13:e071682.

31 Tyndall J, Merlin T, Lockwood C. Impact of findings from grey literature on the outcomes of systematic reviews on interventions to prevent obesity among children: a systematic review. *JBI Evidence Synthesis* 2012;10:1-14.

32 Wikipedia. List of Children's hospitals. 2023; Available at: https://en.wikipedia.org/wiki/List_of_children%27s_hospitals. Accessed 18/03/2023.

33 Sayers A. Tips and tricks in performing a systematic review. *British Journal of General Practice* 2008;58:136.

For peer review only - http://bmjopen.bmj.com/site/about/guidelines.xhtml

34 Peters MD, Godfrey CM, Khalil H, et al. Guidance for conducting systematic scoping reviews. *JBI Evidence Implementation* 2015;13:141-6.

35 Birmingham Women's and Children's NHS Foundation Trust. New pilot project reduces 'was not brought' rates for children with hospital appointments. 2023; Available at: https://bwc.nhs.uk/news/new-pilot-project-reduces-was-not-brought-rates-for-children-with-hospital-appointments-7946. Accessed 18/03/2023.

36 Sheffield Children's Hospital. Reducing the financial cost – Families Matter. 2022; Available at: https://www.sheffieldchildrens.nhs.uk/patients-and-parents/patienthq/families-matter/. Accessed 18/03/2023.

37 The Sydney Children's Network. Transforming health for a more equitable future. Available at: https://www.schn.health.nsw.gov.au/news/articles/2022/03/transforming-health-for-a-more-equitable-future. Accessed 18/03/2023.

38 Southern Chiefs' Organization. SCO adds Jordan's Principle Coordinator at HSC Winnipeg Children's Hospital. 2022; Available at: https://hsc.mb.ca/wp-content/uploads/SCO-Media-Release-SCO-Adds-JP-Coordinator-at-HSC.pdf.

39 Child Health and Wellbeing Network. Addressing Child Health Inequalities in the North East and North Cumbria - Tackling health inequalities in school age children living in homeless accommodation. *North East and North Cumbria* 2022 doi:https://northeastnorthcumbria.nhs.uk/media/0ybppw33/addressing-child-health-inequalities-project-showcase.pdf.

40 SickKids. Equity, Diversity and Inclusion Strategy. 2022; Accessed 18/03/2023.

41 Alder Hey Children's NHS Foundation Trust. Alder Hey Innovation announces far-reaching strategy to address healthcare challenges faced by children and young people. 2023; Available at: https://www.alderhey.nhs.uk/alder-hey-innovation-announces-far-reaching-strategy-to-address-healthcare-challenges-faced-by-children-and-young-people/. Accessed 18/03/2023.

42 Sheffield Children's NHS Foundation Trust. A clinical strategy for a healthier future. 2022; Available at: https://www.sheffieldchildrens.nhs.uk/download/316/general/58220/a-clinical-strategy-for-a-healthier-future.pdf. Accessed 18/03/2023.

43 Boston Children's Hospital. About Us: Health Equity. 2023; Available at: https://www.childrenshospital.org/about-us/health-equity. Accessed 18/03/2023.

44 Starship. Equity. 2022; Available at: https://starship.org.nz/foundation/equity-ensuring-no-child-is-left-behind/. Accessed 18/03/2023.

45 Guy's and St Thomas' NHS Foundation Trust. NHS rainbow badges promote inclusion. 2019; Available at: https://www.evelinalondon.nhs.uk/about-us/news-events/2019-news/20190321-nhs-rainbow-badges-promote-inclusion.aspx. Accessed 18/03/2023.

46 Boston Children's Hospital. About Us: Office of Health Equity & Inclusion. 2023; Available at: https://www.childrenshospital.org/about-us/health-equity/office-health-equity-inclusion. Accessed 18/03/2023.

For peer review only - http://bmjopen.bmj.com/site/about/guidelines.xhtml

47 Boston Children's Hospital. The Sandra L. Fenwick Institute for Pediatric Health Equity and Inclusion. 2023; Available at: https://www.childrenshospital.org/about-us/health-equity/fenwick-institute. Accessed 18/03/2023.

48 Children's Hospital of Philadelphia. Center for Health Equity. Available at: https://www.chop.edu/centers-programs/center-health-equity. Accessed 18/03/2023.

49 Children's Hospital of Philadelphia. Policy Lab. Health Equity. 2022; Available at: https://policylab.chop.edu/our-research/health-equity. Accessed 18/03/2023.

50 Cincinnati Children's Hospital. Cincinnati Children’s new Center for Child Health Equity named for departing CEO Michael Fisher. 2021; Available at: https://www.cincinnatichildrens.org/news/release/2021/child-health-equity. Accessed 18/03/2023.

51 Seattle Children's Hospital. Center for Diversity and Health Equity. 2023; Available at: https://www.seattlechildrens.org/clinics/diversity-health-equity/. Accessed 18/03/2023.

52 Children's Hospital of Philadelphia. Healthier Together Initiative. 2022; Available at: https://www.chop.edu/centers-programs/healthier-together#parentHorizontalTab4. Accessed 18/03/2023.

53 Holland Bloorview Kids Rehabilitation Hospital. Addressing family needs through the power of community. 2023; Available at: https://hollandbloorview.ca/stories-news-events/news/addressing-family-needs-through-power-community.

54 Queensland Children's Hospital. Aboriginal and Torres Strait Island Support. 2022; Available at: https://www.sunshinecoast.health.qld.gov.au/patients-and-visitors/aboriginal-and-torres-strait-islander-support#section__aboriginal-and-torres-strait-islander-hospital-liaison-officers. Accessed 18/03/2023.

55 Boston Children's Hospital. COVID-19 exposed health inequities. These doctors let people know. 2021; Available at: https://answers.childrenshospital.org/covid-19-health-inequities-twitter/. Accessed 18/03/2023.

56 SickKids. Advancing Health Equity at the Hospital for Sick Children. 2019; Available at: https://www.health.belgium.be/sites/default/files/uploads/fields/fpshealth_theme_file/karmali_health_equity_brussels_nov2019.pdf. Accessed 18/03/2023.

57 British Columbia Children's Hospital. Social Pediatrics. 2023; Available at: http://www.bcchildrens.ca/our-services/clinics/social-pediatrics#RICHER--Initiative.

58 The Sydney Children's Hospitals Network. Priority Populations programs given multi-million dollar boost. 2021; Available at: https://www.schn.health.nsw.gov.au/news/articles/2021/10/priority-populations-programs-given-multi-million-dollar-boost.

59 Boston Children's Hospital. Community Asthma Initiative. 2023; Available at: https://www.childrenshospital.org/programs/community-asthma-initiative. Accessed 18/03/2023.

60 Petticrew M. Emerging challenges in evidence-based public health and how to address them. *Journal of Health Monitoring* 2020;5:7.

For peer review only - http://bmjopen.bmj.com/site/about/guidelines.xhtml

61 Klepac B, Krahe M, Spaaij R, et al. Increasing research evidence translation and utilisation to improve population health outcomes 2022.

62 Song F, Loke Y, Hooper L. Why are medical and health-related studies not being published? A systematic review of reasons given by investigators. *PloS one* 2014;9:e110418.

63 Wahowiak L. Community needs assessments leading to better outcomes: ACA requirement fortifying health. *The Nation's health* 2017:1-8.

64 Healthwatch. Cost of living: People are increasingly avoiding NHS appointments and prescriptions. 2023; Available at: https://www.healthwatch.co.uk/news/2023-01-09/cost-living-people-are-increasingly-avoiding-nhs-appointments-and-prescriptions#:~:text=The%20impact%20the%20cost%20of%20living%20crisis%20is,prescriptions%20and%20over-the-counter%20medications%20because%20of%20the%20costs.

For peer review only

For peer review only - http://bmjopen.bmj.com/site/about/guidelines.xhtml

Appendix I: Search terms

Database	Search	Terms
Overton	1	(child OR children OR paediatric OR pediatric OR "young people") AND (hospital) AND ("health inequality" OR "health disparity" OR "health equity")
	2	title: equity child
	3	title: inequality child
	4	title: children hospital equity
	5	title: addressing inequalities
	6	title: addressing inequity
Open Grey	1	Health inequality
	2	Health equity
	3	Health Disparity
Openmd.com	1	(child OR children OR paediatric OR pediatric OR "young people") AND (hospital) AND ("health inequality" OR "health disparity" OR "health equity")
Tripdatabase	1	title: inequalities children hospital
	2	title: address health equity
	3	addressing child health inequality hospital
Duckduckgo	1	hospital based approaches to addressing health inequalities
	2	how do childrens hospitals reduce inequalities
	3	(child OR children OR paediatric OR pediatric OR "young people") AND (hospital) AND ("health inequality" OR "health disparity" OR "health equity")
Google	1	(child OR children OR paediatric OR pediatric OR "young people") AND (hospital) AND ("health inequality" OR "health disparity" OR "health equity")
	2	how do childrens hospitals reduce inequalities
	3	childrens hospital approach to inequalities
	4	Childrens hospital strategy to reduce health inequalities
APHA	1	Childrens hospital
	2	child health inequality
	3	child equity
	4	child health disparity
WHO	1	children's hospital
	2	health inequality
	3	child health equity
	4	health equity
	5	health disparity
NHS England	1	childrens hospital
	2	health inequality
	3	health equity
	4	health disparity
	5	health inequality
OHID	1	"children's hospital"
	2	"child health inequalities"
	3	"health inequalities" AND "hospital"
HMIC	1	“inequalities”
Hospital websites	All	Inequal* OR inequity OR disparit*

For peer review only - http://bmjopen.bmj.com/site/about/guidelines.xhtml

Appendix II: Data extraction form

**Study details and characteristics**	
Reference/ weblink	
Hospital name	
Country of study	
Year published/ website updated	
Population and sample size	
Context (healthcare system)	
Publication type (e.g. report/ website/ news article	
Stand alone hospital / part of network	
**Details of the approach**	
Type of approach (e.g. strategy/ centre..)	
Aim of the approach	
Inequality in focus	
Population in focus	
Setting	
Length of intervention?	
Staff	
**Results extracted from study** (in relation to the concept of the scoping review)	
Main Outcomes/ Results	
Additional outcomes/ any differential effects	
Key findings/ Conclusions	
How were effects measured?	
Other notes	

For peer review only - http://bmjopen.bmj.com/site/about/guidelines.xhtml

Figure 1 – Flow diagram of searches and screening

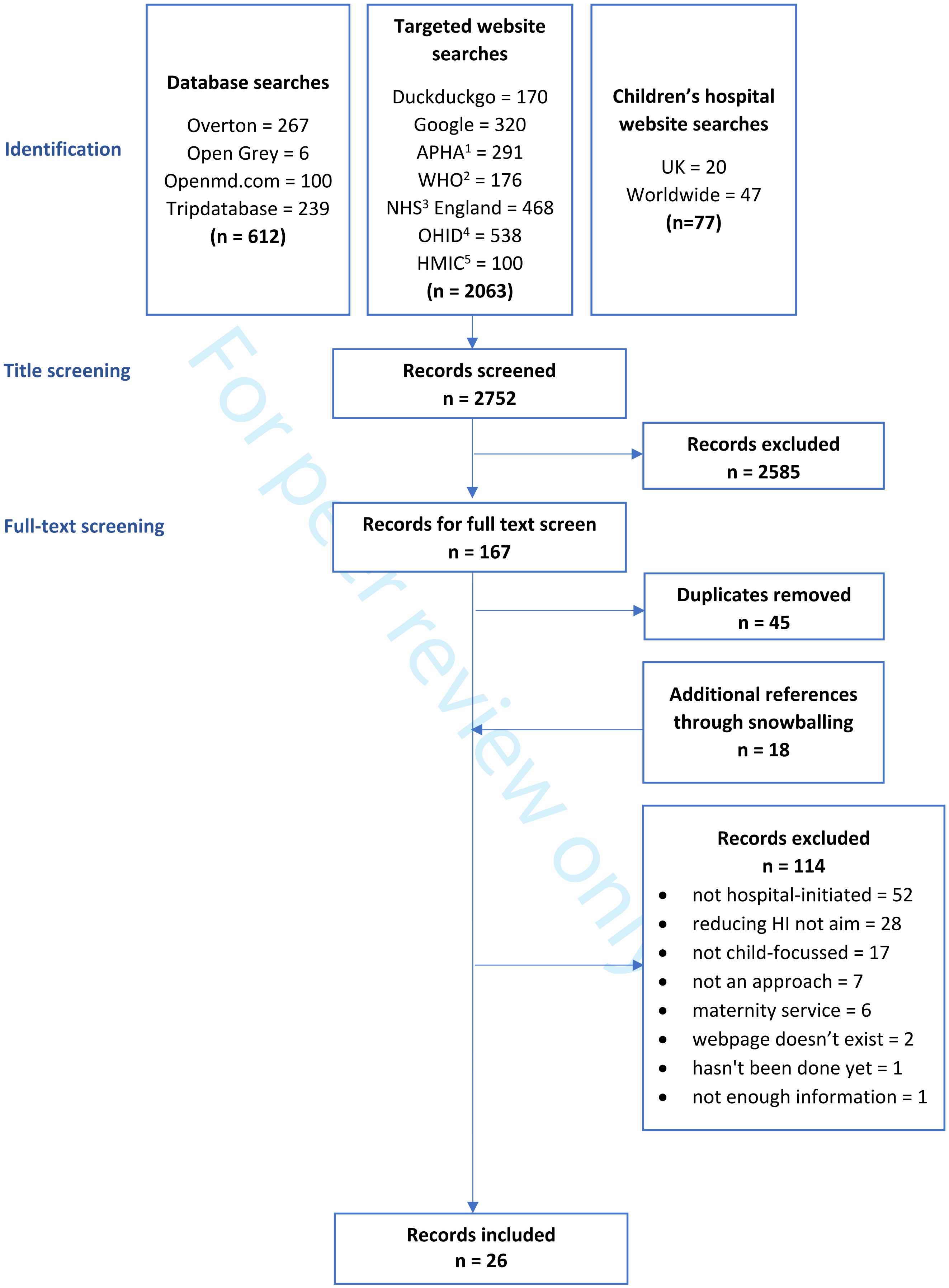

[1] American Public Health Association, [2] World Health Organization, [3] National Health Service, [4] Office for Health Improvement and Disparities, [5] Health Management Information Consortium

For peer review only

Table 1. Summary of included approaches

In-text ref	Approach	Country	Hospital	Approach	Details	Year	Inequity	Setting	Population	Outcomes reported	Staff/ resources involved	Other useful learning	Evaluation/ measure of success
35	Micro	England	Birmingham Women and Childrens Hospital	Intervention	Pilot project targeting families at risk of nonattendance - offered free transport to the hospital	2023 Pilot	Social determinants of health	Hospital	Families in most deprived areas	90% of the study group taking up the offer of help with travel and another 140 appointments that were reallocated.	Administration staff worked extra hours making phone calls, supported by Trust's interpreter team	Staff also started to develop better relationships with the people they were contacting, helping to build trust and increase engagement with their local community	Number of uptake, reduction in WNB
36	Micro	England	Sheffield Children's Hospital	Intervention	Families Matter Project - aimed to provide parents with resources to allow them to maintain basic hygiene from the point of admission, ensure that no families go hungry by developing a food pledge and supporting families by signposting them to resources to support them financially alongside the development of access to Citizen's advice booths.	2022	Social determinants of health	Hospital	All	Positive feedback from families on the provision of basic care resources, particularly sanitary products. Staff members have found that families are less stressed about how they will look after themselves when in patients, it has also been a positive for staff wellbeing in taking care of our families.	All staff	N/A	Qualitative feedback from parents
37	Macro	Australia	Sydney Children's Hospital Network	Strategy	Strategy with interventions as a result such as projects/ increasing number of aboriginal staff, coordinator role for Aboriginal Health Outcomes and Equity Project (aiming to ensure the constant delivery of high-quality, equitable services to Aboriginal children)	2018	General	Hospital and community	Aboriginal children	Aboriginal workforce has grown by 20 staff in clinical and non-clinical roles. Established new dashboard to look at real time data and if aboriginal patients have been seen by an Aboriginal health worker within 24 hours of admission	All staff withing the hospital network, the Aboriginal Health Outcomes and Equity Project Manager, Aboriginal Health Workers	"The advantage of addressing Aboriginal health as part of a children's hospital network is that we have a wide range of services and, therefore, an opportunity to intervene early in the life course."	Not reported
38	Micro	Canada	Health Sciences Centre Children's hospital Winnipeg	Intervention	Employed a 'Jordan's Principal Coordinator - The Jordan's Principle Coordinator at HSC Children's Hospital will assist First Nation children and youth under 18 and their families in accessing programs, services, and supports to improve health, education, and social outcomes.	2022	Access	Hospital	First nation children	None reported	Coordinator and team	N/A	Not reported
39	Micro	England	Newcastle Upon Tyne Hospitals NHS Trust	Intervention	Health contact for school aged children living in homeless accommodation (previously only offered to <5 year olds). Children and parents are offered an appointment for a health assessment on two days per month, initially for a three-month pilot period	2022	Access	Community	Children living in homeless accommodation	In the three-month trial period, 20 school age children were offered health assessments. 17 out of those 20 have had the health assessments. 10 children were supported with oral hygiene needs, with four referrals into treatment at acute level, six had vision needs and four were referred to Ophthalmology for treatment. Five had incomplete immunisations, three received referrals to tuberculosis service, and two had an increased BMI.	Two public health school nurses	The service has used this model of service delivery to support health assessments for asylum seekers locally, and all children under 19 have received health assessments prior to being rehoused. Staff visit the asylum-seeking families' temporary accommodation once per week. Also plan to offer the same whole family support to families in a domestic abuse refuge.	Number of children offered health assessments and number of health interactions that resulted from those (e.g. referrals to a certain service, immunisations)
39	Meso	England	Gateshead Health NHS Foundation Trust	Service	Single point of access for refugee and asylum seekers. Undertake a health needs assessment and remove barriers by connecting families directly to the right health, statutory or voluntary services.	2022	Access	Community	Refugees and Asylum Seekers	Over 140 children had comprehensive health assessments in a secondary care clinic, and dozens of families had similar assessments in a local asylum seeker accommodation through primary care. "Health needs that would have been unrecognised and unmet have been addressed, improving health outcomes for these families." Based on these data, a project spanning primary and secondary care, linking a wide range of other services, has been commissioned to further improve capacity and quality of this work.	Not specified	Engagement of the various stakeholders is critical for this approach to be successful.	Numbers of appointments

For peer review only

Table 1. Summary of included approaches

In-text ref	Approach	Country	Hospital	Approach	Details	Year	Inequity	Setting	Population	Outcomes reported	Staff/ resources involved	Other useful learning	Evaluation/ measure of success
40	Macro	Canada	Hospital for Sick Children Toronto (SickKids)	Strategy	EDI strategy and indigenous health council aims to make care more equitable for indigenous people, ultimately striving to improve child health outcomes.	2020	General	Hospital and community	All	None reported	Patients, EDI Committee	N/A	Measurement and evaluation of disparities in access to care; research and education to minimize variation
41	Macro	England	Alder Hey, Liverpool	Strategy	Innovation strategy and associated projects e.g. analysis of 'was not brought' (WNB) children data to identify families at risk of nonattendance. Other innovation e.g. transparent masks	2022 pilot	General	Hospital	All	WNB brought project- AI- ability to calculate the likelihood of whether a child will come to their appointment or not, and so make an early intervention: piloted on 34 patients in general paediatrics, model has 80% accuracy.	Innovation team	Planning to share WNB project AI tool with other Trusts	Success of tools e.g. accuracy of AI predictor
42	Macro	England	Sheffield Children's Hospital	Strategy	Strategy seeks to minimise the impact of health inequalities on mental and physical health every time a family interacts with us to improve experience, access, and outcomes. Working with partners to address the root causes of inequalities and invest where the need is greatest.	2022	General	Hospital and advocating wider	All	None reported	Not specified	N/A	Not reported
43	Macro	USA	Boston Children's Hospital	Declaration and goals	To address racial injustice and health disparities, Boston Children's released a Declaration on Equity, Diversity, and Inclusivity. The declaration established six goals to elevate health equity as an enterprise priority, core to everything the hospital does.	2020	General	Hospital	All	Six goals committing to addressing inclusion, recruitment, structural racism, employee education, leading on elimination of and metrics to track health disparities. No other outcomes reported	Chief Equity and Inclusion Officer	N/A	Not reported
44	Macro	New Zealand	Starship Hospital, Auckland	Ethos	The Starship Foundation is committed to meeting children and their whānau where they are, ensuring all children in New Zealand have access to world-class healthcare regardless of their ethnicity, where they live, or their family circumstance, decreasing ethnic health disparities in Māori Children, seeking to improve the health journey for tamariki Māori and their whānau, to ultimately achieve equitable outcomes.	2019	General	Hospital and community	All	Online content resonates strongly with Māori and Pacific audiences, with 667,006 post engagements reflecting strong connection and cut-through, Healthy Homes supported 492 households and fitted 237 homes with thermal curtains.	All staff, Starship Child Health has now embedded a Māori Health Team	Produce clinical guidelines for other hospitals	Yearly impact reports
45	Macro	England	Evelina	Intervention	Staff initiative, NHS Rainbow Badges are being worn by staff. When staff sign up to wear the badge they are provided with information about the challenges people who identify as LGBT+ can face accessing healthcare and what they can do to support them. Wearing the badge is a sign that the wearer is someone patients can talk to about issues of sexuality and gender identity.	2019	Access	Hospital	Young LGBT+	Almost a third of staff have signed up to wear and be trained. This has since been rolled out across NHS Trusts in the UK and an associated toolkit developed. In 2021 the NHS Rainbow badge moved from a purely visual symbol to also incorporating an assessment and accreditation mode for NHS Trust.	All staff	Rolled out across other Trusts in England	Uptake
46	Meso	USA	Boston Children's Hospital	Established department/ centre	Office of Health Equity and Inclusion. Coordinates equity, diversity and inclusion initiatives across the hospital.	2017	General	Hospital	All	Initiatives such as professional development seminars and training, career advancement and pipeline programs, mentoring, research, development of metrics	Led by Chief Equity and Inclusion Officer	Work in partnership with Harvard Medical School and other partners	Not reported
47	Meso	USA	Boston Children's Hospital	Established research centre/ portfolio	Institute for Paediatric Health Equity: focuses on 3 areas: health equity research, inclusion, and public policy. Ensuring equitable health care access, delivery, and outcomes for all children.	2021	General	Hospital	All	Research projects to recruit diverse families, studies investigating missed appointments and culturally effective interventions. Seed grants to fund inequalities research.	Led by Senior Vice President. Also, Chief Equity and Inclusion Officer, Paediatric Radiologist	N/A	Not reported
48	Meso	USA	Children's Hospital of Philadelphia (CHOP)	Established department/ centre	Established Center for health equity: 4 core workstreams: Community Translation, Clinical Quality and Safety, Advocacy and Social Justice, and Research and Education.	2022	General	Hospital and community	All	Formation of a Neighbourhood Council	Led by senior manager. Employ community consultant for each core area of focus and a village of community health workers. The Neighbourhood Council will consist of CHOP employees who live in the West Philadelphia area.	Has website and repository of resources for professionals	Not reported

For peer review only

Table 1. Summary of included approaches

In-text ref	Approach	Country	Hospital	Approach	Details	Year	Inequity	Setting	Population	Outcomes reported	Staff/ resources involved	Other useful learning	Evaluation/ measure of success
49	Meso	USA	Children's Hospital of Philadelphia (CHOP)	Established research centre/ portfolio	'Policy Lab': A research portfolio investigating how policies and programs can improve the health of historically marginalized children and adolescents. Advancing health equity through a research lens. PolicyLab is a Center of Emphasis within Children's Hospital of Philadelphia's Research Institute	2022	General	Online, hospital, community	Minority infants and mothers, LGBTQ children, immigrant families, low-income families	Research articles and multiple projects including a brachial plexus recruitment study. Before the health equity intervention, 54% of diverse families and 35% of white families agreed to participate in the study. After implementing the recommended strategies, 68% of diverse families and 71% of white families enrolled in the study.	Team includes 35 faculty and more than 60 staff who are experts in medicine, public health, social work, psychology, law, biostatistics, health services research and population health	Database/ repository of projects and publications. Inclusive recruitment strategies help recruit patients into trials, so that the study population is more representative of the real population. Simple modifications are required to completely change the outcome of recruitment – "it's simple things that make a difference."	Brachial plexus study - the % of diverse and % of white families that were invited to participate in the research that decided to take part (changes pre to post study)
50	Meso	USA	Cincinnati Children's	Established department/ centre	The Center for Child Equity will build equitable care capabilities among staff through training, consultation and partnerships with research and clinical divisions, diversity and community relations teams, as well as a broad coalition of families and other external stakeholders.	2021	General	Hospital and community	All	None reported	Hospital, community and social care partners	N/A	Not reported
51	Meso	USA	Seattle Children's Hospital	Established department/ centre	Center for Diversity and Health Equity (CDHE). Includes patient and family education programme, interpreter services, cultural navigation, family resource centre (kitchen, showers, computers, laundry, TV, Wi-Fi, parent support, phone chargers etc.), guest services, spiritual care, resources for transgender patients. Health Equity Research Grants Program: to fund project to advance work around health equity, have Education and resources for healthcare professionals on equity, and a programme around human trafficking.	2021	General	Hospital	All	Programme of work around human trafficking including video workshops and video. Training for staff around health equity.	Diversity and Health Equity team: 14 people, work closely with the Odessa Brown Children's Clinic, Seattle Children's Research Institute and many other groups within Seattle Children's	Created a self-assessment toolkit for staff to assess performance towards health equity.	Not reported
52	Meso	USA	Children's Hospital of Philadelphia (CHOP)	Partnership Initiative	To target the social determinants of health. Healthier Together partners with community groups, nonprofits and government agencies to learn from each other and multiply the impact of the work. Residents and community organisations participate in oversight/governance meetings, and outreach for program development. Advisers include experts from across CHOP, residents from West and Southwest Philadelphia, and representatives from the School District of Philadelphia, Philadelphia Department of Public Health and other services	2019	Social determinants of health	Hospital and community	Children in three zip codes in West and Southwest Philadelphia	$10 million invested in the community<br>13,450 individuals served with 21 partners and collaborators<br>$3,840 back to families on average through financial counselling and tax prep<br>136 homes renovated to reduce asthma triggers<br>45,570+ healthy meals distributed to families	Hospital and partners	Purchase goods and services from local companies that value a diverse workforce and provide meaningful work opportunities to underrepresented members of our community. Healthier Together partners with community groups, nonprofits and government agencies to learn from each other and multiply the impact of the work. Residents and community organisations participate in oversight/governance meetings, and outreach for program development.	$ invested in community, individuals served, homes renovated, meals distributed
53	Meso	Canada	Holland Bloorview Kids Rehabilitation Hospital, Toronto	Screening tool	Created a social needs screening tool, used during appointments by trained clinicians to identify the social needs and barriers of clients and their families. Family Navigation Hub was created: a resource that offers one-on-one consultations and services as well as monthly virtual workshops in partnership with external organizations, known as the CommunityCONNECT series, for families to access and to help close the gaps when it comes to housing, food, childcare, respite and community connection. A partnership with FoodShare to look at food security needs.	2023	Social determinants of health	Hospital and community	Children with a disability	Community Kitchens Series from FoodShare Toronto: families who register are sent ingredients to prepare culturally diverse recipes. They prepare the meals together with other families while on Zoom, Housing support, social needs screening tool for clinicians.	Screened by clinicians, if eligible family support specialists work with families	The FoodShare program has brought families together and provided a sense of community.	Qualitative feedback from parents

For peer review only

Table 1. Summary of included approaches

In-text ref	Approach	Country	Hospital	Approach	Details	Year	Inequity	Setting	Population	Outcomes reported	Staff/ resources involved	Other useful learning	Evaluation/ measure of success
54	Meso	Australia	Queensland Childrens Hospital, Brisbane	Service	Indigenous liaison service - to support, advocate, provide assistance to indigenous people accessing health and social services	2020	Outcomes	Hospital	Children and families from Aboriginal and Torres Strait Islander communities	None reported	Indigenous liaison officers	N/A	Not reported
55	Micro	USA	Boston Children's Hospital	Advocacy	Analysed data on impact of Covid-19 on communities of colour. Raised awareness on structural racism and differences in outcomes. Communication through Twitter	2021	Outcomes	Online	Communities of colour	The posts reached more than 100,000 people, with reposts by hospitals and several state legislators.	Group of paediatricians	Analysing data showed that rates of Covid-19 infection, serious illness, and death were far higher in Black populations throughout the country. Recognized that data can play an important role in driving more equitable decisions and policies. Social media can be a useful medium to spread awareness, but it has its limitations as well.	Number of people reached on Twitter, number of reposts.
56	Micro	Canada	Hospital for Sick Children Toronto (SickKids)	Screening tool	Developed a paediatric data tool and health equity survey. Also provided training for staff	2019	General	Hospital	All	Increased focus on equity. Better data collection. Staff trained	Clerical staff collected data	Registration is an optimal point in the healthcare visit to collect sociodemographic information. Patients and caregivers willing to provide information	Satisfaction of parents in giving information. Number and percentage of tools completed. Number of staff trained
57	Micro	Canada	British Columbia Children's Hospital Vancouver	Service	Primary healthcare outreach clinics for children, youth, and families in Vancouver's inner-city neighbourhoods. the RICHER program seeks to address health equity disparities and particularly disparities in health care access that many marginalized and vulnerable children and families experience. The model recognizes the importance of trusted relationships and seeks to increase capacity and resilience for the families served.	2023	Access	Community	Inner city families	A wide range of community outreach services at a wide range of centres	Nurse Practitioners, general paediatricians, development paediatricians, Adolescent Health and Medicine doctors, Child and Adolescent Psychiatry physicians, dermatology doctor, research manager	N/A	Not reported
58	Micro	Australia	Sydney Children's Hospital Network	Intervention	Two new projects - the RuralKidsGPS project and PEACH project. Aim to improve equity and access to healthcare services. RuralKids will trial integrated care in rural districts and PEACH will establish technological solutions and sustainable systems for complex needs	2021	Access	Community	Rural populations and socioeconomically disadvantaged communities	RuralKidsGPS: Paediatric care co-ordination has already shown promising results in metropolitan Sydney. Over a two-year period, it demonstrated a 40 per cent reduction in emergency department presentations and a 42 per cent decrease in day-only admissions – saving more than 50,000km of travel for families.	Care coordinators will work closely with local care teams and families to create shared care plans, support technology-enabled healthcare, and connect families with local services to meet the child's needs.	N/A	The success of the RuralKidsGPS and PEACH will be evaluated in the next few years with the intention to roll out both programs more widely across the state
59	Micro	USA	Boston Children's Hospital	Intervention	Case management and home visits by a community health worker for children from low-income families of colour with asthma	2023	Outcomes	Community	Children with asthma from low-income families of colour	By 2022, there were more than 2,491 patients who have enrolled in the Community Asthma Initiative. Their involvement led to: 82% reduction in the percentage of patients with asthma-related hospitalization, 55% reduction in the percentage of patients with Emergency Department visits, 45% reduction in the percentage of patients with missed school days for children, 55% reduction in the percentage of patients with lost work days for parents	Community health workers	Beneficial to wider health of children involved	Changes in the percentage (%) of patients with asthma-related hospitalization, % of patients with Emergency Department visits, % of patients with missed school days for children, % of patients with lost workdays for parents

For peer review only

PRISMA 2020 Checklist

Section and Topic	Item #	Checklist item	Location where item is reported
**TITLE**			
Title	1	Identify the report as a scoping review.	Pg 5
**ABSTRACT**			
Abstract	2	See the PRISMA 2020 for Abstracts checklist.	Pg 2
**INTRODUCTION**			
Rationale	3	Describe the rationale for the review in the context of existing knowledge.	Pg 4
Objectives	4	Provide an explicit statement of the objective(s) or question(s) the review addresses.	Pg 4
**METHODS**			
Eligibility criteria	5	Specify the inclusion and exclusion criteria for the review and how studies were grouped for the syntheses.	Pg 5
Information sources	6	Specify all databases, registers, websites, organisations, reference lists and other sources searched or consulted to identify studies. Specify the date when each source was last searched or consulted.	Pg 5
Search strategy	7	Present the full search strategies for all databases, registers and websites, including any filters and limits used.	Pg 17
Selection process	8	Specify the methods used to decide whether a study met the inclusion criteria of the review, including how many reviewers screened each record and each report retrieved, whether they worked independently, and if applicable, details of automation tools used in the process.	Pg 5
Data collection process	9	Specify the methods used to collect data from reports, including how many reviewers collected data from each report, whether they worked independently, any processes for obtaining or confirming data from study investigators, and if applicable, details of automation tools used in the process.	Pg 5
Data items	10a	List and define all outcomes for which data were sought. Specify whether all results that were compatible with each outcome domain in each study were sought (e.g. for all measures, time points, analyses), and if not, the methods used to decide which results to collect.	Pg 6
	10b	List and define all other variables for which data were sought (e.g. participant and intervention characteristics, funding sources). Describe any assumptions made about any missing or unclear information.	Pg 18
Study risk of bias assessment	11	Specify the methods used to assess risk of bias in the included studies, including details of the tool(s) used, how many reviewers assessed each study and whether they worked independently, and if applicable, details of automation tools used in the process.	N/A
Effect measures	12	Specify for each outcome the effect measure(s) (e.g. risk ratio, mean difference) used in the synthesis or presentation of results.	N/A
Synthesis methods	13a	Describe the processes used to decide which studies were eligible for each synthesis (e.g. tabulating the study intervention characteristics and comparing against the planned groups for each synthesis (item #5)).	Pg 6
	13b	Describe any methods required to prepare the data for presentation or synthesis, such as handling of missing summary statistics, or data conversions.	Pg 6
	13c	Describe any methods used to tabulate or visually display results of individual studies and syntheses.	Pg 6
	13d	Describe any methods used to synthesize results and provide a rationale for the choice(s). If meta-analysis was performed, describe the model(s), method(s) to identify the presence and extent of statistical heterogeneity, and software package(s) used.	Pg 6
	13e	Describe any methods used to explore possible causes of heterogeneity among study results (e.g. subgroup analysis, meta-regression).	N/A
	13f	Describe any sensitivity analyses conducted to assess robustness of the synthesized results.	N/A
Reporting bias assessment	14	Describe any methods used to assess risk of bias due to missing results in a synthesis (arising from reporting biases).	N/A
Certainty	15	Describe any methods used to assess certainty (or confidence) in the body of evidence for an outcome.	N/A

For peer review only - http://bmjopen.bmj.com/site/about/guidelines.xhtml

PRISMA 2020 Checklist

Section and Topic	Item #	Checklist item	Location where item is reported
assessment			
**RESULTS**			
Study selection	16a	Describe the results of the search and selection process, from the number of records identified in the search to the number of studies included in the review, ideally using a flow diagram.	Pg 6
	16b	Cite studies that might appear to meet the inclusion criteria, but which were excluded, and explain why they were excluded.	N/A
Study characteristics	17	Cite each included study and present its characteristics.	Pgs 6 – 8 and Table 1
Risk of bias in studies	18	Present assessments of risk of bias for each included study.	N/A
Results of individual studies	19	For all outcomes, present, for each study: (a) summary statistics for each group (where appropriate) and (b) an effect estimate and its precision (e.g. confidence/credible interval), ideally using structured tables or plots.	N/A
Results of syntheses	20a	For each synthesis, briefly summarise the characteristics and risk of bias among contributing studies.	Table 1
	20b	Present results of all statistical syntheses conducted. If meta-analysis was done, present for each the summary estimate and its precision (e.g. confidence/credible interval) and measures of statistical heterogeneity. If comparing groups, describe the direction of the effect.	N/A
	20c	Present results of all investigations of possible causes of heterogeneity among study results.	N/A
	20d	Present results of all sensitivity analyses conducted to assess the robustness of the synthesized results.	N/A
Reporting biases	21	Present assessments of risk of bias due to missing results (arising from reporting biases) for each synthesis assessed.	N/A
Certainty of evidence	22	Present assessments of certainty (or confidence) in the body of evidence for each outcome assessed.	N/A
**DISCUSSION**			
Discussion	23a	Provide a general interpretation of the results in the context of other evidence.	Pg 9
	23b	Discuss any limitations of the evidence included in the review.	Pg 10
	23c	Discuss any limitations of the review processes used.	Pg 10
	23d	Discuss implications of the results for practice, policy, and future research.	Pgs 10-11
**OTHER INFORMATION**			
Registration and protocol	24a	Provide registration information for the review, including register name and registration number, or state that the review was not registered.	N/A
	24b	Indicate where the review protocol can be accessed, or state that a protocol was not prepared.	Pg 5
	24c	Describe and explain any amendments to information provided at registration or in the protocol.	Pg 5
Support	25	Describe sources of financial or non-financial support for the review, and the role of the funders or sponsors in the review.	Pg 11
Competing interests	26	Declare any competing interests of review authors.	Pg 11
Availability of data, code and other materials	27	Report which of the following are publicly available and where they can be found: template data collection forms; data extracted from included studies; data used for all analyses; analytic code; any other materials used in the review.	Pgs 18 and Table 1

For peer review only - http://bmjopen.bmj.com/site/about/guidelines.xhtml

From: Page MJ, McKenzie JE, Bossuyt PM, Boutron I, Hoffmann TC, Mulrow CD, et al. The PRISMA 2020 statement: an updated guideline for reporting systematic reviews. BMJ 2021;372:n71. doi: 10.1136/bmj.n71

PRISMA 2020 Checklist

For more information, visit: http://www.prisma-statement.org/

For peer review only

BMJ Open

How do children's hospitals address health inequalities: a grey literature scoping review

Journal:	*BMJ Open*
Manuscript ID	bmjopen-2023-079744.R1
Article Type:	Original research
Date Submitted by the Author:	13-Dec-2023
Complete List of Authors:	Brennan, Louise; Lancaster University, Medical School; St Helens and Knowsley Teaching Hospitals NHS Trust<br>Stres, Dora Pestotnik; Lancaster University, Lancaster Medical School<br>Egboko, Fiona; Lancaster University, Lancaster Medical School<br>Patel, Pallavi; Lancaster University, Lancaster Medical School; St Helens and Knowsley Teaching Hospitals NHS Trust,<br>Broad, Eleanor; Dumfries and Galloway Royal Infirmary<br>Brewster, Liz; Lancaster University, Lancaster Medical School<br>Lunn, Judith; Lancaster University, Lancaster Medical School<br>Isba, Rachel; Lancaster University, Lancaster Medical School; Alder Hey Children's NHS Foundation Trust
**Primary Subject Heading**:	Public health
Secondary Subject Heading:	Paediatrics
Keywords:	Health Equity, PAEDIATRICS, PUBLIC HEALTH

SCHOLARONE™
Manuscripts

I, the Submitting Author has the right to grant and does grant on behalf of all authors of the Work (as defined in the below author licence), an exclusive licence and/or a non-exclusive licence for contributions from authors who are: i) UK Crown employees; ii) where BMJ has agreed a CC-BY licence shall apply, and/or iii) in accordance with the terms applicable for US Federal Government officers or employees acting as part of their official duties; on a worldwide, perpetual, irrevocable, royalty-free basis to BMJ Publishing Group Ltd ("BMJ") its licensees and where the relevant Journal is co-owned by BMJ to the co-owners of the Journal, to publish the Work in this journal and any other BMJ products and to exploit all rights, as set out in our licence.

The Submitting Author accepts and understands that any supply made under these terms is made by BMJ to the Submitting Author unless you are acting as an employee on behalf of your employer or a postgraduate student of an affiliated institution which is paying any applicable article publishing charge ("APC") for Open Access articles. Where the Submitting Author wishes to make the Work available on an Open Access basis (and intends to pay the relevant APC), the terms of reuse of such Open Access shall be governed by a Creative Commons licence – details of these licences and which Creative Commons licence will apply to this Work are set out in our licence referred to above.

Other than as permitted in any relevant BMJ Author's Self Archiving Policies, I confirm this Work has not been accepted for publication elsewhere, is not being considered for publication elsewhere and does not duplicate material already published. I confirm all authors consent to publication of this Work and authorise the granting of this licence.

For peer review only

How do children's hospitals address health inequalities: a grey literature scoping review

Louise Brennan [1, 2] l.brennan2@lancaster.ac.uk

Dora Pestotnik Stres [1] d.pestotnikstres@lancaster.ac.uk

Fiona Egboko [1] f.egboko@lancaster.ac.uk

Pallavi Patel [1,2] pallavi.patel4@nhs.net

Eleanor Broad [3] eleanor.broad@doctors.org.uk

Liz Brewster [1] e.brewster@lancaster.ac.uk

Judith Lunn [1] j.lunn1@lancaster.ac.uk

Rachel Isba [1,4] rachel.isba@lancaster.ac.uk (corresponding author)

[1] Lancaster Medical School, Lancaster University, Lancaster, UK

[2] St Helens and Knowsley Teaching Hospitals NHS Trust, Prescot, UK

[3] Dumfries and Galloway Royal Infirmary, Cargenbridge, Dumfries, UK

[4] Alder Hey Children's NHS Foundation Trust, Liverpool, UK

For peer review only

ABSTRACT (291)

Objectives:

Health inequalities are systematic differences in health between people, that are avoidable and unfair. Globally, more political strategies are required to address health inequalities, which have increased since the global SARS-CoV-2/COVID-19 pandemic, with a disproportionate impact on children.

This scoping review aimed to identify and collate information on how hospitals around the world that deliver care to children have addressed health inequalities.

Design:

Scoping review focussed solely on grey literature due to the appropriateness of that approach for complex, multistakeholder interventions that lack robust data and predictability.

Eligibility criteria for selecting studies: Following Joanna Briggs Institute guidelines, a four-step approach to identifying literature was adopted.

Data extraction and synthesis: Retrieved items were screened against clear inclusion and exclusion criteria before data were extracted by two independent reviewers using a data extraction tool. Studies were tabulated by hospital. Meta-analysis was not conducted due to the varied nature of studies and approaches.

Results:

Our study identified 26 approaches to reduction of health inequalities, from 17 children's hospitals. Approaches were categorised based on their size and scope. Seven approaches were defined as macro, including hospital-wide inequality strategies. Ten approaches were classed as meso, including establishment of new departments and research centres. Micro approaches (n=9) included one-off projects or interventions offered to specific groups/ services. Almost half of the reported approaches did not discuss evaluation of impact.

Conclusions:

Children's hospitals provide a suitable location to conduct public health interventions. This scoping review provides examples of approaches on three scales delivered at hospitals across high-income countries. Hospitals with the most comprehensive and extensive range of approaches employed dedicated staff within the hospital and community. This review indicates the value of recruitment of both public health-trained staff as well as culturally-similar staff to deliver community-based interventions.

For peer review only - http://bmjopen.bmj.com/site/about/guidelines.xhtml

Strengths and limitations:

- Unlike traditional scoping reviews of peer-reviewed literature, this review focusses solely on grey literature.
- An extensive search of online grey literature databases, targeted websites and children's 1hospitals webpages was conducted.
- Grey literature may be more appropriate for areas of rapidly changing research such as new approaches to addressing health inequalities.
- Items were only included if available in English or translatable through online apps.

Key words:

Health equity, paediatrics, public health

Word Count: 3,833

For peer review only

INTRODUCTION

Health inequalities are systematic differences in health between people, that are avoidable and unfair.[1] Whilst most-commonly researched in relation to socioeconomic deprivation [2,3,4,5], the links between health inequalities and other types of social inequality such as disability,[6] race,[7] religious beliefs [8] and sexual orientation [9] are also well-evidenced.

Health inequalities are evident between and within countries across the world. Two examples of this are life expectancy and under-5s mortality rates. In sub-Saharan Africa, children are 14 times more likely to die before the age of 5 than in the rest of the world.[10] In India, the under-5 mortality rate was 106 per 1000 births to mothers with no education, compared with 49 per 1000 births to mothers with secondary or higher education.[11] In the UK, life expectancy is also socially patterned, with, for example, people residing in the most deprived areas of England living an average of 14 years less than those in the least deprived areas.[12]

Disparities in health outcomes are also evident globally. Research conducted in the USA found that rates of low birthweight were three times higher amongst babies born to Black mothers with no college education, compared with babies born to white mothers with a college education.[13] In the UK, children living in deprived areas are more likely to suffer from conditions such as tooth decay, asthma, diabetes and obesity.[14]

However, health inequalities are not just measured in life expectancy and health outcomes; patterns in accessing care are also correlated to socioeconomic status. In New Zealand, Māori young people are the least likely to be enrolled with a Primary Health Care provider.[15] In the UK, children from the most deprived areas are less likely to be brought to outpatient appointments,[16] but more likely to attend the Emergency Department, than those living in the least deprived areas.[17,18]

Whilst the World Health Organization (WHO) has promoted the reduction of health inequalities as a global priority since its inaugural World Health Report in 1995,[19], political strategies to address the social determinants of health are still required in both high-, middle- and low-income countries.[20] In the UK, the National Health Service (NHS; a government-funded, free at the point of access, universal medical and healthcare service) has a legal duty to address health inequalities,[21] and this was made a key commitment in the *NHS Long Term Plan.*[22] However, the SARS-CoV-2/COVID-19 pandemic has widened health inequalities [23] and critics have argued that the absence of a national health inequalities strategy has led to local healthcare systems – including hospitals – struggling to develop their own approach to addressing health inequalities.[24]

Recognising the disproportionately negative impact of the pandemic on children,[25, 26] and lack of central steer on health inequalities in children and young people (CYP) in the UK [27, 28] and other countries,[29] this scoping review aimed to identify and collate information on how hospitals around the world that deliver care to children have addressed health inequalities. The findings of this scoping review are intended to be used to inform children's hospitals on approaches, outcomes and experiences based on previous work, to influence future interventions and policy.

Review Questions

1. What approaches do hospitals take to address health inequalities in children and young people?

2. What health inequalities do the approaches focus on?

3. How is effectiveness measured and demonstrated?

For peer review only - http://bmjopen.bmj.com/site/about/guidelines.xhtml

METHODS

As presented in the published protocol,[30] this scoping review focussed solely on grey literature due to the appropriateness of that approach for complex, multistakeholder interventions that lack robust data and predictability.[31] Grey literature is also more appropriate for topics that are under current review and moving at a fast pace, as is the case with interventions to address health inequalities. Furthermore, as hospitals and clinicians often have less capacity to publish academic literature in peer-reviewed journals, this methodology was deemed more appropriate as it allowed for a deeper, more systematic search of the internet and hospital websites.

Inclusion and exclusion criteria

As detailed in the protocol, items were included if initiated by a hospital, published in English, post-2010, focussed on children and with a clear aim to address health inequalities. Items were excluded if they did not explicitly state reducing health inequalities in their aim/ focus, and if the item did not describe an approach. Whole hospital approaches (including adults) and antenatal and prenatal services were also excluded.

Search Strategy

During January 2023, the online databases Overton, Open Grey, Openmd and tripdatabase were searched using the search terms outlined in the protocol. Secondly, search engines duckduckgo (allowing searches unaffected by previous search histories) and Google (using the anonymised search function) were searched using the search terms detailed in Appendix 1. The first 10 pages of returns were screened, unless items appeared to have continuing relevancy past 10 pages. Thirdly, targeted websites of the American Public Health Association (APHA), WHO, NHS England, the UK's Office for Health Improvement and Disparities (OHID) and the Health Management Information Consortium (HMIC) were explored using their search functions.

Fourth, as an add-on to the original search strategy, a list of worldwide children's hospitals was identified through Wikipedia (a free online, publicly editable encyclopaedia).[32] The authors searched the full list of children's hospital's websites individually, using individual website search bars and key terms. Non-English websites were translated using the Google Translate function.

Finally, snowballing was conducted at all stages of the search, where additional resources are identified through citations or weblinks.[33]

The full search strategy and terms is detailed in Appendix 1.

Study selection and data extraction

Searches and results were managed using Microsoft Excel. All items were screened based on their title (and where necessary associated text). Items deemed relevant were recorded by saving their website link. All items retrieved in this title screening stage were then screened in full against the inclusion and exclusion criteria by two independent reviewers. Any uncertainties or disagreements were first discussed between the two reviewers and then resolved by a third reviewer (RI). Data extraction was carried out by two separate reviewers, using a data extraction form developed using JBI guidelines [34] (Appendix 2). Where more than one approach was identified from the same hospital, they were classed as separate (and classed as a unique item) if they had separate aims. In some cases, multiple records were identified from one hospital, however they were all relating to the same aim/ approach (e.g. items under one strategy), and in these cases, records were combined into one entry.

For peer review only - http://bmjopen.bmj.com/site/about/guidelines.xhtml

Data synthesis

Following data extraction, studies were tabulated by hospital. Key details of the approach and its associated resources, setting and other key details were also presented alongside any outcome data. Due to the varied nature of studies and approaches no meta-analysis was conducted. Quality assessment was not undertaken due to the grey literature focus and lack of reporting consistency.

Patients and public involvement

No patients or public were involved.

RESULTS

Search results

In total, 2,752 results were returned from our database searches (identification stage). Of these, 2,585 were deemed inappropriate for inclusion, leaving 167 records for full text screening against the inclusion and exclusion criteria. During full text screening a further 18 records were identified through snowballing. Forty-five records were identified as duplicates and excluded. Items were then excluded if they did not meet the inclusion criteria (n=108). Six records were excluded after discussion with a third independent reviewer as consensus could not be met between the initial two reviewers, leading to 115 exclusions in total. Data were extracted from 26 texts (Figure 1).

General characteristics of approaches

Articles included in the results were published online between 2017 and the search date in January 2023. The approaches were from five high-income countries (United States of America, Australia, Canada, UK and New Zealand). Seventeen separate hospitals were included in the results. Boston Children's Hospital initiated five of the included approaches, Children's Hospital of Philadelphia (CHOP) initiated three separate approaches, with The Hospital for Sick Children Toronto (SickKids) and Sheffield Children's Hospital each initiating two; the remainder of hospitals had one record each. A summary of the included approaches is presented in supplementary Table 1. Whilst our protocol intended to investigate all hospitals providing care to children, the majority of our results originated from stand-alone children's hospitals.

Categorisation of approaches

Approaches were categorised into three themes based on the size and reach of the approach they were describing and are detailed below. 'Macro' approaches were organisation-wide, impacting the activity of the whole children's hospital, e.g. a formally-stated strategy. The approaches were classified as 'meso' if they involved setting up a new department or would change the activity of more than one department or service. 'Micro' approaches were single interventions, or projects that were short term or small in scale, e.g. reducing missed outpatient appointments. Almost half of the total 26 approaches (n=12) aimed to address a broad range of inequalities for the whole paediatric population. Meso or micro level approaches were more likely to focus on specific inequalities (e.g. financial hardship [35, 36]) or inequalities in access or outcomes for specific groups (e.g. Aboriginal children [37] First Nation Children [38] or refugees and asylum seekers).[39]

Macro

Seven approaches were categorised as macro. Four of these were hospital-wide strategies, specifically aimed to address: health equity (Australia);[37] equity, diversity and inclusion (EDI)

For peer review only - http://bmjopen.bmj.com/site/about/guidelines.xhtml

(Canada);[40] health inequalities within an innovation strategy (UK);[41] and inclusion of health inequalities in a clinical strategy (UK).[42]

Two hospitals had an 'ethos of equity' – in Boston this was formalised through a declaration and goals.[43] At the Starship Hospital in New Zealand this was not formalised in a strategy but is conceptualised as a commitment in all that they do.[44]

The final macro approach to health inequalities began as a micro approach but has since become hospital-wide and replicated across the UK. The *rainbow badge initiative* is for staff, who, when signing up to wear a rainbow badge receive information and training about the challenges that people who identify as LGBT+ can face when accessing healthcare and what they can do to support them.[45]

Meso.

Ten approaches were characterised as meso. Four of the US hospitals had established a centre aiming to address equity with dedicated staff. In Boston, the *Office for Health Equity and Inclusion*, aimed to coordinate EDI initiatives across the whole hospital [46] and the *Fenwick Institute*, focused on research into paediatric health equity.[47] The latter has provided funding for inequalities research and delivered research projects, including investigation into missed appointments and effective interventions to recruit diverse families. CHOP also established a *Center for Health Equity* [48] and a research *Policy Lab*, which aimed to advance health equity through a research lens.[49] The Cincinnati Children's Hospital's *Center for Child Equity* aimed to build equitable care capabilities in staff,[50] and Seattle Children's Hospital's *Center for Diversity and Health Equity* focused on patient and family education programmes.[51] These latter hospitals have no separate research centre but include research in their equity approach.

Alongside their centre, CHOP had also established the *Healthier Together* partnership, together with community groups and non-profit government agencies to invest money into the community, families and individuals. Interventions included distribution of healthy meals, home renovation and financial counselling.[52] In Toronto, Canada, the *Family Navigation Hub* was established following the development of a social needs screening tool.[53] The hub aimed to help families to access and close the gaps around availability of housing, food, childcare, respite and community connection.[49]

Several meso level approaches focused on specific patient groups. An *Indigenous Liaison Service,* established in Queensland Children's Hospital, Australia, targeted support, advocacy and provided assistance to indigenous people accessing health and social services.[54] Using the nationally-established *Jordan's Principle*, which ensures that First Nations children living in Canada can access services when/ as they are needed, the Health Sciences Centre Children's Hospital (Winnipeg) employed a *Jordan's Principle* co-ordinator to assist First Nations children and young people to access care at their facility.[38] Similarly, in Gateshead, England, a service had been developed to provide a single point of access for refugee and asylum-seeking children and families. This aimed to reduce barriers to healthcare by connecting families to the right health, statutory or voluntary service leading to referrals for oral health, obesity, immunisations and the tuberculosis (TB) service.[39]

Micro

Nine approaches were categorised as 'micro' meaning they were projects or interventions used across one service/ department rather than at a larger scale. Three projects originated in England. The *Families Matter* project in Sheffield aimed to help parents with financial cost of staying in

For peer review only - http://bmjopen.bmj.com/site/about/guidelines.xhtml

hospital, and provided resources to allow them to maintain basic hygiene and needs, including sanitary products, food and additional support such as signposting to additional providers of financial advice. At the time of data extraction, the hospital was piloting a free breakfast for all parents staying with their child. Feedback from staff and families was positive, with staff reporting that caregivers appeared less stressed as a result of the enhanced offers of support.[36] In Birmingham, a pilot project was running to offer free transport to families at risk of non-attendance at the hospital. Pilot data showed that 90% of the study group took up the offer of help and 140 appointments were relocated during the eight weeks.[35] Thirdly, Newcastle upon Tyne Hospitals NHS Trust trialled assigning a health contact, who conducted health assessments for school-aged children living in homeless accommodation. In the three-month pilot, 17 of the 20 children assigned a health contact had health assessments, leading to support with oral health, ophthalmology treatment, referrals for immunisations and access to the TB service.[39] Data collection and analysis was the focus of two projects that fell into the micro approach category. During the SARS-CoV-2/COVID-19 pandemic, Boston Children's Hospital analysed data on the impact of the pandemic on communities of colour and published the findings through Twitter. The posts reached more than 100,000 people, raising awareness and reaching state legislators.[55] SickKids, Canada developed a paediatric data tool and health equity survey as part of a data collection pilot. Clerical staff collected data and 3,000 staff received training on culturally-competent care. Parents and carers reported satisfaction with the data tool and registration was identified as the optimal point in the healthcare visit to collect sociodemographic data.[56]

Community outreach projects were common in the approaches we found. In Canada, led by British Columbia Children's Hospital, the *RICHER* (Responsive Intersectoral Child and Community Health Education and Research) programme was designed to address disparities in access to primary care for children, youth and families in Vancouver's inner cities.[57] A project run by Sydney Children's Hospital aimed to improve access and equity for children with complex needs using technology and those living in rural districts. They demonstrated a 40% reduction in Emergency Department visits and a 42% decrease in day admissions, saving more than 50,000km travel for families.[58] In Boston, children from low-income families of colour, with asthma, were the target population for case management and home visits by a community health worker. An 82% reduction in asthma-related hospitalisation rates was reported in the 2,491 enrolled patients. Other outcomes included 55% reduction in lost workdays for parents and 45% reduction in missed school days.[59]

Evaluation tools

Nearly half (n=12) of the approaches reported no evaluation/ measure of success in their communication. Those hospitals reporting their outcomes used a range of different measures, with uptake/ number of people involved being the most common (n=10). Several approaches used caregiver satisfaction (n=3), with qualitative findings providing powerful quotes from service users.[53] Other less common measures were number of staff trained (n=2), accuracy of prediction tool (n=1), and money invested in the wider community by the hospital (n=1). Those hospitals that attempted to quantify their impact on health inequalities reported outcomes such as homes renovated [52] or modified,[44] change in research study uptake in diverse families,[49] reductions in hospitalisation, missed days from work and lost days at school,[59] reduction in emergency admissions and miles travelled.[58]

Staffing/ resources

Approaches varied in their approaches to staffing. Shorter-term projects used existing staff such as clerical staff for data collection,[56, 35] whereas new centres/ dedicated departments reported new

For peer review only - http://bmjopen.bmj.com/site/about/guidelines.xhtml

staff structures with dedicated teams and additional infrastructure. For community outreach or targeted interventions with specific communities, positive outcomes were attributed to culturally-similar staffing.[44, 37, 54, 38] Taking the service out into the community was also reported to improve outcomes for traditionally-marginalised patients, and in these cases, community health workers were often utilised. Approaches did not specify the cost of establishing and delivering their approach and therefore data could not be compared.

DISCUSSION

In this paper we have provided a synthesis of the approaches taken by children's hospitals in addressing health inequalities experienced by children and young people. Our search of grey literature aimed to be a comprehensive way to investigate unpublished literature produced by hospitals and their partners. Whilst our search aimed to investigate all hospitals providing care for children, the results originated solely from children's hospitals.

Our study identified 26 approaches to reduction of health inequalities, from 16 children's hospitals, all in high-income countries. Approaches have been categorised according to their depth and breadth of reach, from macro whole-organisation approaches, down to small micro pilot projects. The majority of approaches were categorised as meso and were commonly newly-established departments or centres dedicated to addressing equity and research into this area.

Twelve approaches had no specific inequality as their focus, instead taking a broad approach to addressing equity. Seven hospitals addressed inequalities in access to healthcare, a further four focussed on social determinants of health and the remaining three addressed health outcomes for specific groups.

Almost half of the approaches failed to report outcomes or their methods of evaluation/ assessment and very few reported costs. Although it is notoriously difficult to demonstrate the impact of public health interventions,[60] there is still a recognised need to build an evidence base around interventions that aim reduce health inequalities and improve population health.[61] It is therefore important for hospitals to better evaluate and communicate their activities and findings in the future.

Few publications relating to hospital approaches to address health inequalities in the academic literature led to the refocus of this scoping review to grey literature only. Previous research has suggested that lack of time is the most common reason for non-publication of medical and health related studies;[62] however, this scoping review highlights the merits of publishing information on websites and other non-peer reviewed repositories of information, whilst also supporting the utilisation of grey literature for reviews of hospital-based interventions.

Hospitals that did evaluate their approaches varied in their evaluation methods. At the most basic level, hospitals counted numbers involved and uptake of interventions. More complex evaluations looked at changes in missed appointments and emergency admissions by demographics such as ethnicity. Other hospitals considered missed school days and days from work for parents and carers. As this is a scoping review, an assessment of study quality was not undertaken, and findings should therefore be interpreted with care.

Hospitals with dedicated centres and a team of staff reported a greater number of more comprehensive outcomes. Hospitals with a dedicated health inequalities focussed strategy, or health inequalities within their wider strategies were also more likely to have an increased level of activity. However, strategy did not always equate to outcomes, and it is therefore important for hospitals to

For peer review only - http://bmjopen.bmj.com/site/about/guidelines.xhtml

consolidate strategy with projects and appropriate staff resources. Projects involving culturally-similar staff or dedicated community teams reported success in reaching historically marginalised communities.

There are several reasons for the possible exclusion of approaches from middle- and low-income countries. Firstly, due to time and resource constraints our search was limited to materials available in the English language. Translate apps were utilised where possible as all searching was completed using the internet, nevertheless this possibly restricted findings from some sites where translation was not accurate enough to identify alternative terms for our keywords. Secondly, it is highly probable that the terminology used to describe health inequalities e.g. disparities and inequities, are not universally adopted around the world, and approaches that were relevant were excluded due to the way the intervention was described. This was confirmed during screening of worldwide children's hospitals websites, where several potentially relevant approaches aimed to provide culturally-appropriate care rather than equity in health, and were therefore excluded. Hence, lack of reporting on approaches does not imply lack of activity in this review. Thirdly the exclusion of hospital-wide, antenatal, prenatal and adult focussed approaches may have led to the exclusion of early years and some family-centred approaches to addressing health inequalities. Similarly, the exclusion of whole-hospital strategies that include adults may explain the results only including children's hospitals. It is possible that hospital-wide strategies existed in non-freestanding children's hospitals, that also addressed inequities in children, however these were not eligible for inclusion in this review.

The context of healthcare systems is important to consider when interpreting these results. The majority of approaches originated from US hospitals, where healthcare is not universal and hospital activity could be driven by factors different to those where healthcare is free at the point of access. Furthermore, since the Affordable Care Act of 2010, American non-profit hospitals are mandated to undertake a community needs assessment and documentation of how they are addressing needs every three years.[63] Similarly, in England there has been a national drive to address health inequalities due to their inclusion in the NHS Long Term Plan [22] and the current 'cost of living crisis' has impacted access to care [64] and fuelled activity to address the cost of hospital appointments for patients.

CONCLUSIONS AND RECOMMENDATIONS

Children's hospitals and hospitals that care for children provide a suitable location to conduct public health interventions. This scoping review has explored and summarised the approaches that some children's hospitals in high-income countries have taken to address health inequalities. Several children's hospitals (mainly in the US) have advanced equity programmes of work, but this may be driven by the regulatory environment.

There was a lack of robust evaluation of the approaches, despite public health interventions requiring appropriate evaluation to enable evidence-based practice.

The lack of published literature on hospital-led approached to health inequalities highlights the need for hospitals to promote the work that they are doing, so areas of good practice and learning can be shared and implemented. As demonstrated here, this could be via hospital websites or through networks. The dearth of peer-reviewed articles also highlights the relevance of grey literature for hospital-based research such as this. The development of further methodological guidance on grey literature-focused scoping and systematic reviews is warranted.

For peer review only - http://bmjopen.bmj.com/site/about/guidelines.xhtml

The findings of this review originated from a small number of high-income countries. It is possible that relevant approaches are being used elsewhere but not being published, and/ or are not labelled as health inequalities. Further research into this would be warranted to complete a broader summary of approaches.

Hospitals with the most comprehensive and extensive range of approaches employ dedicated staff within the hospital and community. This review therefore provides evidence for the value of the recruitment of both public health trained staff as well as culturally-similar staff to deliver community-based interventions.

Contributorship statement

Louise Brennan, Rachel Isba, Liz Brewster and Judith Lunn conceptualised the work. Louise Brennan, Dora Pestotnik Stres, Fiona Egboko, Pallavi Patel and Eleanor Broad were responsible for running searches and screening retrieved items. Louise Brennan and Dora Pestotnik Stres independently extracted data. Discrepancies were discussed with Rachel Isba. Louise Brennan prepared the original draft. Rachel Isba and Liz Brewster critically reviewed and edited the work. All authors edited and agreed the final version and are accountable for the accuracy and integrity of the work.

Competing interests

The authors declare that they have no known competing financial interests or personal relationships that could have appeared to influence the work reported in this paper.

Funding

This work was supported by the National Paediatric Accelerator Programme, now the Children's Hospital Alliance, contracted by Sheffield Children's Hospital, grant number SCH5628.

Data sharing statement

No additional data available.

Acknowledgements

Thank you to Louise Speakman, Lancaster University who kindly advised on the original search strategy.

For peer review only - http://bmjopen.bmj.com/site/about/guidelines.xhtml

References

1 Williams E, Buck D, Babalola G, et al. What are health inequalities?. *The King's Fund* 2022.

2 Marmot M. Fair society, healthy lives. *Fair society, healthy lives* 2013:1-74.

3 Graham H. Unequal lives: McGraw-Hill Education (UK) 2007.

4 Acheson D. Independent Inquiry into Inequalities in Health. *Science* 1998;10:217-33.

5 Patel JA, Nielsen F, Badiani AA, et al. Poverty, inequality and COVID-19: the forgotten vulnerable. *Public Health* 2020;183:110.

6 Emerson E, Baines S, Allerton L, et al. Health inequalities and people with learning disabilities in the UK 2011.

7 Elwell-Sutton T, Deeny S, Stafford M. Emerging findings on the impact of COVID-19 on black and minority ethnic people. *The Health Foundation* 2020.

8 Office for National Statistics. Religion and Health in England and Wales. 2020; Available at: https://www.ons.gov.uk/peoplepopulationandcommunity/culturalidentity/religion/articles/religionandhealthinenglandandwales/february2020. Accessed 30/06/2023.

9 Booker CL, Rieger G, Unger JB. Sexual orientation health inequality: evidence from understanding society, the UK longitudinal household study. *Prev Med* 2017;101:126-32.

10 World Health Organization. Health Inequalities and their causes. 2018; Available at: https://www.who.int/news-room/facts-in-pictures/detail/health-inequities-and-their-causes. Accessed 30/06/2023.

11 Balarajan Y, Selvaraj S, Subramanian SV. Health care and equity in India. *The Lancet* 2011;377:505-15.

12 Rashid T, Bennett JE, Paciorek CJ, et al. Life expectancy and risk of death in 6791 communities in England from 2002 to 2019: high-resolution spatiotemporal analysis of civil registration data. *The Lancet Public Health* 2021;6:e805-16.

13 Pollock EA, Gennuso KP, Givens ML, et al. Trends in infants born at low birthweight and disparities by maternal race and education from 2003 to 2018 in the United States. *BMC Public Health* 2021;21:1117.

14 Wickham S, Anwar E, Barr B, et al. Poverty and child health in the UK: using evidence for action. *Arch Dis Child* 2016;101:759-66.

15 Irurzun-Lopez M, Jeffreys M, Cumming J. The enrolment gap: who is not enrolling with primary health organizations in Aotearoa New Zealand and what are the implications? An exploration of 2015–2019 administrative data. *International journal for equity in health* 2021;20:1-11.

For peer review only - http://bmjopen.bmj.com/site/about/guidelines.xhtml

16 French LR, Turner KM, Morley H, et al. Characteristics of children who do not attend their hospital appointments, and GPs' response: a mixed methods study in primary and secondary care. *British Journal of General Practice* 2017;67:e483-9.

17 Jarvis S, Livingston J, Childs A, et al. Outpatient appointment non-attendance and unplanned health care for children and young people with neurological conditions: a retrospective cohort study. *Developmental Medicine & Child Neurology* 2019;61:840-6.

18 Scantlebury R, Rowlands G, Durbaba S, et al. Socioeconomic deprivation and accident and emergency attendances: cross-sectional analysis of general practices in England. *British Journal of General Practice* 2015;65:e649-54.

19 King NB, Harper S, Young ME. Who cares about health inequalities? Cross-country evidence from the World Health Survey. *Health Policy Plan* 2013;28:558-71.

20 Rebouças P, Falcão IR, Barreto ML. Social inequalities and their impact on children's health: a current and global perspective. *J Pediatr* 2022;98:55-65.

21 Equality Act. Equality Act 2010.

22 NHS. The NHS long term plan. *NHS* 2019.

23 Buck D, Lewis T. NHS England and tackling inequalities: times are changing. *The Kings Fund* 2021.

24 Ford J, Sowden S, Olivera J, et al. Transforming health systems to reduce health inequalities. *Future Healthcare Journal* 2021;8:e204.

25 McLellan A, Abbasi K. The NHS is not living with covid, it's dying from it. *BMJ* 2022;378.

26 Pickett K, Taylor-Robinson D, Erlam J. Child of the North: Building a fairer future after COVID-19 2021.

27 Bradley SH. The ethics and politics of addressing health inequalities. *Clinical Medicine* 2021;21:147.

28 Choudry M. Tackling health inequalities and promoting prevention: If not now, then when?. *Future Healthcare Journal* 2021;8:4.

29 Dickman SL, Himmelstein DU, Woolhandler S. Inequality and the health-care system in the USA. *The Lancet* 2017;389:1431-41.

30 Brennan L, Brewster L, Lunn J, et al. How do hospitals address health inequalities experienced by children and young people: a grey literature scoping review protocol. *BMJ open* 2023;13:e071682.

31 Tyndall J, Merlin T, Lockwood C. Impact of findings from grey literature on the outcomes of systematic reviews on interventions to prevent obesity among children: a systematic review. *JBI Evidence Synthesis* 2012;10:1-14.

32 Wikipedia. List of Children's hospitals. 2023; Available at: https://en.wikipedia.org/wiki/List_of_children%27s_hospitals. Accessed 18/03/2023.

For peer review only - http://bmjopen.bmj.com/site/about/guidelines.xhtml

33 Sayers A. Tips and tricks in performing a systematic review. *British Journal of General Practice* 2008;58:136.

34 Peters MD, Godfrey CM, Khalil H, et al. Guidance for conducting systematic scoping reviews. *JBI Evidence Implementation* 2015;13:141-6.

35 Birmingham Women's and Children's NHS Foundation Trust. New pilot project reduces 'was not brought' rates for children with hospital appointments. 2023; Available at: https://bwc.nhs.uk/news/new-pilot-project-reduces-was-not-brought-rates-for-children-with-hospital-appointments-7946. Accessed 18/03/2023.

36 Sheffield Children's Hospital. Reducing the financial cost – Families Matter. 2022; Available at: https://www.sheffieldchildrens.nhs.uk/patients-and-parents/patienthq/families-matter/. Accessed 18/03/2023.

37 The Sydney Children's Network. Transforming health for a more equitable future. Available at: https://www.schn.health.nsw.gov.au/news/articles/2022/03/transforming-health-for-a-more-equitable-future. Accessed 18/03/2023.

38 Southern Chiefs' Organization. SCO adds Jordan's Principle Coordinator at HSC Winnipeg Children's Hospital. 2022; Available at: https://hsc.mb.ca/wp-content/uploads/SCO-Media-Release-SCO-Adds-JP-Coordinator-at-HSC.pdf.

39 Child Health and Wellbeing Network. Addressing Child Health Inequalities in the North East and North Cumbria - Tackling health inequalities in school age children living in homeless accommodation. *North East and North Cumbria* 2022 doi:https://northeastnorthcumbria.nhs.uk/media/0ybppw33/addressing-child-health-inequalities-project-showcase.pdf.

40 SickKids. Equity, Diversity and Inclusion Strategy. 2022; Accessed 18/03/2023.

41 Alder Hey Children's NHS Foundation Trust. Alder Hey Innovation announces far-reaching strategy to address healthcare challenges faced by children and young people. 2023; Available at: https://www.alderhey.nhs.uk/alder-hey-innovation-announces-far-reaching-strategy-to-address-healthcare-challenges-faced-by-children-and-young-people/. Accessed 18/03/2023.

42 Sheffield Children's NHS Foundation Trust. A clinical strategy for a healthier future. 2022; Available at: https://www.sheffieldchildrens.nhs.uk/download/316/general/58220/a-clinical-strategy-for-a-healthier-future.pdf. Accessed 18/03/2023.

43 Boston Children's Hospital. About Us: Health Equity. 2023; Available at: https://www.childrenshospital.org/about-us/health-equity. Accessed 18/03/2023.

44 Starship. Equity. 2022; Available at: https://starship.org.nz/foundation/equity-ensuring-no-child-is-left-behind/. Accessed 18/03/2023.

45 Guy's and St Thomas' NHS Foundation Trust. NHS rainbow badges promote inclusion. 2019; Available at: https://www.evelinalondon.nhs.uk/about-us/news-events/2019-news/20190321-nhs-rainbow-badges-promote-inclusion.aspx. Accessed 18/03/2023.

For peer review only - http://bmjopen.bmj.com/site/about/guidelines.xhtml

46 Boston Children's Hospital. About Us: Office of Health Equity & Inclusion. 2023; Available at: https://www.childrenshospital.org/about-us/health-equity/office-health-equity-inclusion. Accessed 18/03/2023.

47 Boston Children's Hospital. The Sandra L. Fenwick Institute for Pediatric Health Equity and Inclusion. 2023; Available at: https://www.childrenshospital.org/about-us/health-equity/fenwick-institute. Accessed 18/03/2023.

48 Children's Hospital of Philadelphia. Center for Health Equity. Available at: https://www.chop.edu/centers-programs/center-health-equity. Accessed 18/03/2023.

49 Children's Hospital of Philadelphia. Policy Lab. Health Equity. 2022; Available at: https://policylab.chop.edu/our-research/health-equity. Accessed 18/03/2023.

50 Cincinnati Children's Hospital. Cincinnati Children's new Center for Child Health Equity named for departing CEO Michael Fisher. 2021; Available at: https://www.cincinnatichildrens.org/news/release/2021/child-health-equity. Accessed 18/03/2023.

51 Seattle Children's Hospital. Center for Diversity and Health Equity. 2023; Available at: https://www.seattlechildrens.org/clinics/diversity-health-equity/. Accessed 18/03/2023.

52 Children's Hospital of Philadelphia. Healthier Together Initiative. 2022; Available at: https://www.chop.edu/centers-programs/healthier-together#parentHorizontalTab4. Accessed 18/03/2023.

53 Holland Bloorview Kids Rehabilitation Hospital. Addressing family needs through the power of community. 2023; Available at: https://hollandbloorview.ca/stories-news-events/news/addressing-family-needs-through-power-community.

54 Queensland Children's Hospital. Aboriginal and Torres Strait Island Support. 2022; Available at: https://www.sunshinecoast.health.qld.gov.au/patients-and-visitors/aboriginal-and-torres-strait-islander-support#section__aboriginal-and-torres-strait-islander-hospital-liaison-officers. Accessed 18/03/2023.

55 Boston Children's Hospital. COVID-19 exposed health inequities. These doctors let people know. 2021; Available at: https://answers.childrenshospital.org/covid-19-health-inequities-twitter/. Accessed 18/03/2023.

56 SickKids. Advancing Health Equity at the Hospital for Sick Children. 2019; Available at: https://www.health.belgium.be/sites/default/files/uploads/fields/fpshealth_theme_file/karmali_health_equity_brussels_nov2019.pdf. Accessed 18/03/2023.

57 British Columbia Children's Hospital. Social Pediatrics. 2023; Available at: http://www.bcchildrens.ca/our-services/clinics/social-pediatrics#RICHER--Initiative.

58 The Sydney Children's Hospitals Network. Priority Populations programs given multi-million dollar boost. 2021; Available at: https://www.schn.health.nsw.gov.au/news/articles/2021/10/priority-populations-programs-given-multi-million-dollar-boost.

59 Boston Children's Hospital. Community Asthma Initiative. 2023; Available at: https://www.childrenshospital.org/programs/community-asthma-initiative. Accessed 18/03/2023.

For peer review only - http://bmjopen.bmj.com/site/about/guidelines.xhtml

60 Petticrew M. Emerging challenges in evidence-based public health and how to address them. *Journal of Health Monitoring* 2020;5:7.

61 Klepac B, Krahe M, Spaaij R, et al. Increasing research evidence translation and utilisation to improve population health outcomes 2022.

62 Song F, Loke Y, Hooper L. Why are medical and health-related studies not being published? A systematic review of reasons given by investigators. *PloS one* 2014;9:e110418.

63 Wahowiak L. Community needs assessments leading to better outcomes: ACA requirement fortifying health. *The Nation's health* 2017:1-8.

64 Healthwatch. Cost of living: People are increasingly avoiding NHS appointments and prescriptions. 2023; Available at: https://www.healthwatch.co.uk/news/2023-01-09/cost-living-people-are-increasingly-avoiding-nhs-appointments-and-prescriptions#:~:text=The%20impact%20the%20cost%20of%20living%20crisis%20is,prescriptions%20and%20over-the-counter%20medications%20because%20of%20the%20costs.

Figure 1 – Flow diagram of searches and screening.

Supplementary table 1 – Detailed summary of included approaches.

For peer review only

Figure 1 – Flow diagram of searches and screening

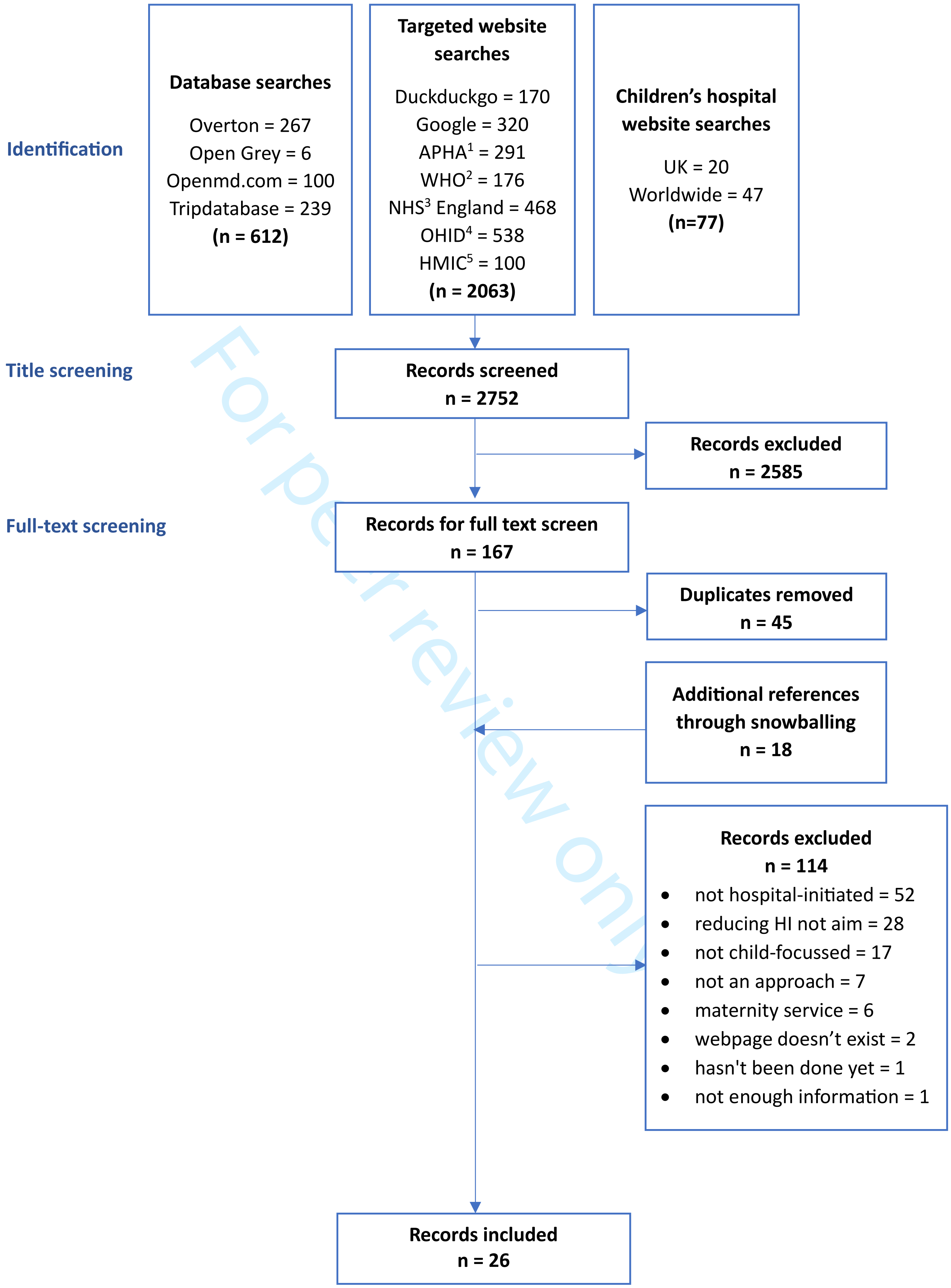

[1] American Public Health Association, [2] World Health Organization, [3] National Health Service, [4] Office for Health Improvement and Disparities, [5] Health Management Information Consortium

For peer review only

Table 1. Detailed summary of included approaches

In-text ref	Country	Hospital	Approach	Type	Details	Year	Inequity	Setting	Population	Outcomes reported	Staff/ resources involved	Other useful learning	Evaluation/ measure of success
35	England	Birmingham Women and Childrens Hospital	Intervention	Micro	Pilot project targeting families at risk of nonattendance - offered free transport to the hospital.	2023 Pilot	Social determinants of health	Hospital	Families in most deprived areas	90% of the study group took up the offer of help with travel and another 140 appointments that were reallocated.	Administration staff worked extra hours making phone calls, supported by Trust's interpreter team.	Staff also started to develop better relationships with the people they were contacting, helping to build trust and increase engagement with their local community.	Number of uptake, reduction in WNB.
36	England	Sheffield Children's Hospital	Intervention	Micro	Families Matter Project - aimed to provide parents with resources to allow them to maintain basic hygiene from the point of admission, ensure that no families go hungry by developing a food pledge and supporting families by signposting them to resources to support them financially alongside the development of access to Citizen's Advice booths.	2022	Social determinants of health	Hospital	All	Positive feedback from families on the provision of basic care resources, particularly sanitary products. Staff members have found that families are less stressed about how they will look after themselves when in patients, it has also been a positive for staff wellbeing in taking care of our families.	All staff	N/A	Qualitative feedback from parents.
37	Australi a	Sydney Children's Hospital Network	Strategy	Macro	Strategy with interventions as a result such as projects/ increasing number of Aboriginal staff, coordinator role for Aboriginal Health Outcomes and Equity Project (aiming to ensure the constant delivery of high-quality, equitable services to Aboriginal children).	2018	General	Hospital and community	Aboriginal children	Aboriginal workforce has grown by 20 staff in clinical and non-clinical roles. Established new dashboard to look at real time data and if aboriginal patients have been seen by an Aboriginal health worker within 24 hours of admission.	All staff withing the hospital network, the Aboriginal Health Outcomes and Equity Project Manager, Aboriginal Health Workers	"The advantage of addressing Aboriginal health as part of a children's hospital network is that we have a wide range of services and, therefore, an opportunity to intervene early in the life course."	Not reported.
38	Canada	Health Sciences Centre Children's hospital Winnipeg	Intervention	Micro	Employed a 'Jordan's Principal Coordinator' - The Jordan's Principle Coordinator at HSC Children's Hospital will assist First Nation children and youth under 18 and their families in accessing programs, services, and supports to improve health, education, and social outcomes.	2022	Access	Hospital	First nation children	None reported	Coordinator and team	N/A	Not reported.
39	England	Newcastle Upon Tyne Hospitals NHS Trust	Intervention	Micro	Health contact for school-aged children living in homeless accommodation (previously only offered to <5 year olds). Children and parents are offered an appointment for a health assessment on two days per month, initially for a three-month pilot period.	2022	Access	Community	Children living in homeless accommodation	In the three-month trial period, 20 school age children were offered health assessments. 17 out of those 20 have had the health assessments. Ten children were supported with oral hygiene needs, with four referrals into treatment at acute level, six had vision needs and four were referred to Ophthalmology for treatment. Five had incomplete immunisations, three received referrals to tuberculosis service, and two had an increased BMI.	Two public health school nurses	The service has used this model of service delivery to support health assessments for asylum seekers locally, and all children under 19 have received health assessments prior to being rehoused. Staff visit the asylum-seeking families' temporary accommodation once per week. Also plan to offer the same whole family support to families in a domestic abuse refuge.	Number of children offered health assessments and number of health interactions that resulted from those (e.g. referrals to a certain service, immunisations).
39	England	Gateshead Health NHS Foundation Trust	Service	Meso	Single point of access for refugee and asylum seekers. Undertake a health needs assessment and remove barriers by connecting families directly to the right health, statutory or voluntary services.	2022	Access	Community	Refugees and Asylum Seekers	Over 140 children had comprehensive health assessments in a secondary care clinic, and dozens of families had similar assessments in a local asylum seeker accommodation through primary care. "Health needs that would have been unrecognised and unmet have been addressed, improving health outcomes for these families." Based on these data, a project spanning primary and secondary care, linking a wide range of other services, has been commissioned to further improve capacity and quality of this work.	Not specified	Engagement of the various stakeholders is critical for this approach to be successful.	Numbers of appointments.

For peer review only

Table 1. Detailed summary of included approaches

In-text ref	Country	Hospital	Approach	Type	Details	Year	Inequity	Setting	Population	Outcomes reported	Staff/ resources involved	Other useful learning	Evaluation/ measure of success
40	Canada	Hospital for Sick Children Toronto (SickKids)	Strategy	Macro	EDI strategy and indigenous health council aims to make care more equitable for indigenous people, ultimately striving to improve child health outcomes.	2020	General	Hospital and community	All	None reported.	Patients, EDI Committee	N/A	Measurement and evaluation of disparities in access to care; research and education to minimize variation .
41	England	Alder Hey, Liverpool	Strategy	Macro	Innovation strategy and associated projects e.g. analysis of 'was not brought' (WNB) children data to identify families at risk of nonattendance. Other innovation e.g. transparent masks	2022 pilot	General	Hospital	All	WNB brought project- AI- ability to calculate the likelihood of whether a child will come to their appointment or not, and so make an early intervention: piloted on 34 patients in general paediatrics, model has 80% accuracy.	Innovation team	Planning to share WNB project AI tool with other Trusts.	Success of tools e.g. accuracy of AI predictor.
42	England	Sheffield Children's Hospital	Strategy	Macro	Strategy seeks to minimise the impact of health inequalities on mental and physical health every time a family interacts with us to improve experience, access, and outcomes. Working with partners to address the root causes of inequalities and invest where the need is greatest.	2022	General	Hospital and advocating wider	All	None reported.	Not specified	N/A	Not reported.
43	USA	Boston Children's Hospital	Declaration and goals	Macro	To address racial injustice and health disparities, Boston Children's released a Declaration on Equity, Diversity, and Inclusivity. The declaration established six goals to elevate health equity as an enterprise priority, core to everything the hospital does.	2020	General	Hospital	All	Six goals committing to addressing inclusion, recruitment, structural racism, employee education, leading on elimination of and metrics to track health disparities. No other outcomes reported.	Chief Equity and Inclusion Officer	N/A	Not reported.
44	New Zealand	Starship Hospital, Auckland	Ethos	Macro	The Starship Foundation is committed to meeting children and their whānau where they are, ensuring all children in New Zealand have access to world-class healthcare regardless of their ethnicity, where they live, or their family circumstance, decreasing ethnic health disparities in Māori Children, seeking to improve the health journey for tamariki Māori and their whānau, to ultimately achieve equitable outcomes.	2019	General	Hospital and community	All	Online content resonates strongly with Māori and Pacific audiences, with 667,006 post engagements reflecting strong connection and cut-through, Healthy Homes supported 492 households and fitted 237 homes with thermal curtains.	All staff, Starship Child Health has now embedded a Māori Health Team	Produce clinical guidelines for other hospitals.	Yearly impact reports.
45	England	Evelina	Intervention	Macro	NHS Rainbow Badges are being worn by staff. When staff sign up to wear the badge they are provided with information about the challenges people who identify as LGBT+ can face accessing healthcare and what they can do to support them. Wearing the badge is a sign that the wearer is someone patients can talk to about issues of sexuality and gender identity.	2019	Access	Hospital	Young LGBT+	Almost a third of staff have signed up to wear and be trained. This has since been rolled out across NHS Trusts in the UK and an associated toolkit developed. In 2021 the NHS Rainbow badge moved from a purely visual symbol to also incorporating an assessment and accreditation mode for NHS Trust.	All staff	Rolled out across other Trusts in England.	Uptake.
46	USA	Boston Children's Hospital	Established department / centre	Meso	Office of Health Equity and Inclusion. Coordinates equity, diversity and inclusion initiatives across the hospital.	2017	General	Hospital	All	Initiatives such as professional development seminars and training, career advancement and pipeline programs, mentoring, research, development of metrics.	Led by Chief Equity and Inclusion Officer	Work in partnership with Harvard Medical School and other partners.	Not reported.
47	USA	Boston Children's Hospital	Established research centre/ portfolio	Meso	Institute for Paediatric Health Equity: focuses on 3 areas: health equity research, inclusion, and public policy. Ensuring equitable health care access, delivery, and outcomes for all children.	2021	General	Hospital	All	Research projects to recruit diverse families, studies investigating missed appointments and culturally effective interventions. Seed grants to fund inequalities research.	Led by Senior Vice President. Also, Chief Equity and Inclusion Officer, Paediatric Radiologist	N/A	Not reported.
48	USA	Children's Hospital of Philadelphia (CHOP)	Established department / centre	Meso	Established Center for health equity: 4 core workstreams: Community Translation, Clinical Quality and Safety, Advocacy and Social Justice, and Research and Education.	2022	General	Hospital and community	All	Formation of a Neighbourhood Council.	Led by senior manager. Employ community consultant for each core area of focus and a village of community health workers. The Neighbourhood Council will consist of CHOP employees who live in the West Philadelphia area.	Has website and repository of resources for professionals.	Not reported.

Table 1. Detailed summary of included approaches

In-text ref	Country	Hospital	Approach	Type	Details	Year	Inequity	Setting	Population	Outcomes reported	Staff/ resources involved	Other useful learning	Evaluation/ measure of success
49	USA	Children's Hospital of Philadelphia (CHOP)	Established research centre/ portfolio	Meso	'Policy Lab': A research portfolio investigating how policies and programs can improve the health of historically marginalized children and adolescents. Advancing health equity through a research lens. PolicyLab is a Center of Emphasis within Children's Hospital of Philadelphia's Research Institute	2022	General	Online, hospital, community	Minority infants and mothers, LGBTQ children, immigrant families, low-income families	Research articles and multiple projects including a brachial plexus recruitment study. Before the health equity intervention, 54% of diverse families and 35% of white families agreed to participate in the study. After implementing the recommended strategies, 68% of diverse families and 71% of white families enrolled in the study.	Team includes 35 faculty and more than 60 staff who are experts in medicine, public health, social work, psychology, law, biostatistics, health services research and population health	Database/ repository of projects and publications.<br>Inclusive recruitment strategies help recruit patients into trials, so that the study population is more representative of the real population.<br>Simple modifications are required to completely change the outcome of recruitment – "it's simple things that make a difference."	Brachial plexus study - the % of diverse and % of white families that were invited to participate in the research that decided to take part (changes pre to post study).
50	USA	Cincinnati Children's	Established department / centre	Meso	The Center for Child Equity builds equitable care capabilities among staff through training, consultation and partnerships with research and clinical divisions, diversity and community relations teams, as well as a broad coalition of families and other external stakeholders.	2021	General	Hospital and community	All	None reported.	Hospital, community and social care partners	N/A	Not reported.
51	USA	Seattle Children's Hospital	Established department / centre	Meso	Center for Diversity and Health Equity (CDHE). Includes patient and family education programme, interpreter services, cultural navigation, family resource centre (kitchen, showers, computers, laundry, TV, Wi-Fi, parent support, phone chargers etc.), guest services, spiritual care, resources for transgender patients.<br>Health Equity Research Grants Program: to fund project to advance work around health equity, have Education and resources for healthcare professionals on equity, and a programme around human trafficking.	2021	General	Hospital	All	Programme of work around human trafficking including video workshops and video. Training for staff around health equity.	Diversity and Health Equity team: 14 people, work closely with the Odessa Brown Children's Clinic, Seattle Children's Research Institute and many other groups within Seattle Children's	Created a self-assessment toolkit for staff to assess performance towards health equity.	Not reported.
52	USA	Children's Hospital of Philadelphia (CHOP)	Partnership Initiative	Meso	To target the social determinants of health. Healthier Together partners with community groups, nonprofits and government agencies to learn from each other and multiply the impact of the work. Residents and community organisations participate in oversight/governance meetings, and outreach for program development. Advisers include experts from across CHOP, residents from West and Southwest Philadelphia, and representatives from the School District of Philadelphia, Philadelphia Department of Public Health and other services.	2019	Social determinants of health	Hospital and community	Children in three zip codes in West and Southwest Philadelphia	$10 million invested in the community. 13,450 individuals served with 21 partners and collaborators. $3,840 back to families on average through financial counselling and tax prep. 136 homes renovated to reduce asthma triggers. 45,570+ healthy meals distributed to families	Hospital and partners	Purchase goods and services from local companies that value a diverse workforce and provide meaningful work opportunities to underrepresented members of our community.<br>Healthier Together partners with community groups, nonprofits and government agencies to learn from each other and multiply the impact of the work. Residents and community organisations participate in oversight/governance meetings, and outreach for program development.	$ invested in community, individuals served, homes renovated, meals distributed.
53	Canada	Holland Bloorview Kids Rehabilitation Hospital, Toronto	Screening tool	Meso	Created a social needs screening tool, used during appointments by trained clinicians to identify the social needs and barriers of clients and their families. Family Navigation Hub was created: a resource that offers one-on-one consultations and services as well as monthly virtual workshops in partnership with external organizations, known as the CommunityCONNECT series, for families to access and to help close the gaps when it comes to housing, food, childcare, respite and community connection. Developed a partnership with FoodShare to look at food security needs.	2023	Social determinants of health	Hospital and community	Children with a disability	Community Kitchens Series from FoodShare Toronto: families who register are sent ingredients to prepare culturally diverse recipes. They prepare the meals together with other families while on Zoom, Housing support, social needs screening tool for clinicians.	Screened by clinicians, if eligible family support specialists work with families	The FoodShare program has brought families together and provided a sense of community.	Qualitative feedback from parents.

For peer review only

Table 1. Detailed summary of included approaches

In-text ref	Country	Hospital	Approach	Type	Details	Year	Inequity	Setting	Population	Outcomes reported	Staff/ resources involved	Other useful learning	Evaluation/ measure of success
54	Australia	Queensland Childrens Hospital, Brisbane	Service	Meso	Indigenous liaison service - to support, advocate, provide assistance to indigenous people accessing health and social services.	2020	Outcomes	Hospital	Children and families from Aboriginal and Torres Strait Islander communities	None reported.	Indigenous liaison officers	N/A	Not reported
55	USA	Boston Children's Hospital	Advocacy	Micro	Analysed data on impact of Covid-19 on communities of colour. Raised awareness on structural racism and differences in outcomes. Communication through Twitter.	2021	Outcomes	Online	Communities of colour	The posts reached more than 100,000 people, with reposts by hospitals and several state legislators.	Group of paediatricians	Analysing data showed that rates of Covid-19 infection, serious illness, and death were far higher in Black populations throughout the country. Recognized that data can play an important role in driving more equitable decisions and policies. Social media can be a useful medium to spread awareness, but it has its limitations as well.	Number of people reached on Twitter, number of reposts.
56	Canada	Hospital for Sick Children Toronto (SickKids)	Screening tool	Micro	Developed a paediatric data tool and health equity survey. Also provided training for staff.	2019	General	Hospital	All	Increased focus on equity. Better data collection. Staff trained.	Clerical staff collected data	Registration is an optimal point in the healthcare visit to collect sociodemographic information. Patients and caregivers willing to provide information.	Satisfaction of parents in giving information. Number and percentage of tools completed. Number of staff trained.
57	Canada	British Columbia Children's Hospital Vancouver	Service	Micro	Primary healthcare outreach clinics for children, youth, and families in Vancouver's inner-city neighbourhoods. the RICHER program seeks to address health equity disparities and particularly disparities in health care access that many marginalized and vulnerable children and families experience. The model recognizes the importance of trusted relationships and seeks to increase capacity and resilience for the families served.	2023	Access	Community	Inner city families	A wide range of community outreach services at a wide range of centres.	Nurse Practitioners, general paediatricians, development paediatricians, Adolescent Health and Medicine doctors, Child and Adolescent Psychiatry physicians, dermatology doctor, research manager	N/A	Not reported.
58	Australia	Sydney Children's Hospital Network	Intervention	Micro	Two new projects - the RuralKidsGPS project and PEACH project. Aim to improve equity and access to healthcare services. RuralKids will trial integrated care in rural districts and PEACH will establish technological solutions and sustainable systems for complex needs.	2021	Access	Community	Rural populations and socioeconomically disadvantaged communities	RuralKidsGPS: Paediatric care co-ordination has already shown promising results in metropolitan Sydney. Over a two-year period, it demonstrated a 40 per cent reduction in emergency department presentations and a 42 per cent decrease in day-only admissions – saving more than 50,000km of travel for families.	Care coordinators will work closely with local care teams and families to create shared care plans, support technology-enabled healthcare, and connect families with local services to meet the child's needs.	N/A	The success of the RuralKidsGPS and PEACH will be evaluated in the next few years with the intention to roll out both programs more widely across the state.
59	USA	Boston Children's Hospital	Intervention	Micro	Case management and home visits by a community health worker for children from low-income families of colour with asthma.	2023	Outcomes	Community	Children with asthma from low-income families of colour	By 2022, there were more than 2,491 patients who enrolled in the Community Asthma Initiative. Their involvement led to: 82% reduction in the percentage of patients with asthma-related hospitalization, 55% reduction in the percentage of patients with Emergency Department visits, 45% reduction in the percentage of patients with missed school days for children, 55% reduction in the percentage of patients with lost work days for parents.	Community health workers	Beneficial to wider health of children involved.	Changes in the percentage (%) of patients with asthma-related hospitalization, % of patients with Emergency Department visits, % of patients with missed school days for children, % of patients with lost workdays for parents.

For peer review only

Appendix I: Search terms

Database	Search	Terms
Overton	1	(child OR children OR paediatric OR pediatric OR "young people") AND (hospital) AND ("health inequality" OR "health disparity" OR "health equity")
	2	title: equity child
	3	title: inequality child
	4	title: children hospital equity
	5	title: addressing inequalities
	6	title: addressing inequity
Open Grey	1	Health inequality
	2	Health equity
	3	Health Disparity
Openmd.com	1	(child OR children OR paediatric OR pediatric OR "young people") AND (hospital) AND ("health inequality" OR "health disparity" OR "health equity")
Tripdatabase	1	title: inequalities children hospital
	2	title: address health equity
	3	addressing child health inequality hospital
Duckduckgo	1	hospital based approaches to addressing health inequalities
	2	how do childrens hospitals reduce inequalities
	3	(child OR children OR paediatric OR pediatric OR "young people") AND (hospital) AND ("health inequality" OR "health disparity" OR "health equity")
Google	1	(child OR children OR paediatric OR pediatric OR "young people") AND (hospital) AND ("health inequality" OR "health disparity" OR "health equity")
	2	how do childrens hospitals reduce inequalities
	3	childrens hospital approach to inequalities
	4	Childrens hospital strategy to reduce health inequalities
APHA	1	Childrens hospital
	2	child health inequality
	3	child equity
	4	child health disparity
WHO	1	children's hospital
	2	health inequality
	3	child health equity
	4	health equity
	5	health disparity
NHS England	1	childrens hospital
	2	health inequality
	3	health equity
	4	health disparity
	5	health inequality
OHID	1	"children's hospital"
	2	"child health inequalities"
	3	"health inequalities" AND "hospital"
HMIC	1	“inequalities”
Hospital websites	All	Inequal* OR inequity OR disparit*

For peer review only

Appendix II: Data extraction form

**Study details and characteristics**	
Reference/ weblink	
Hospital name	
Country of study	
Year published/ website updated	
Population and sample size	
Context (healthcare system)	
Publication type (e.g. report/ website/ news article	
Stand alone hospital / part of network	
**Details of the approach**	
Type of approach (e.g. strategy/ centre..)	
Aim of the approach	
Inequality in focus	
Population in focus	
Setting	
Length of intervention?	
Staff	
**Results extracted from study** (in relation to the concept of the scoping review)	
Main Outcomes/ Results	
Additional outcomes/ any differential effects	
Key findings/ Conclusions	
How were effects measured?	
Other notes	

For peer review only

Preferred Reporting Items for Systematic reviews and Meta-Analyses extension for Scoping Reviews (PRISMA-ScR) Checklist

SECTION	ITEM	PRISMA-ScR CHECKLIST ITEM	REPORTED ON PAGE #
**TITLE**			
Title	1	Identify the report as a scoping review.	1
**ABSTRACT**			
Structured summary	2	Provide a structured summary that includes (as applicable): background, objectives, eligibility criteria, sources of evidence, charting methods, results, and conclusions that relate to the review questions and objectives.	2
**INTRODUCTION**			
Rationale	3	Describe the rationale for the review in the context of what is already known. Explain why the review questions/objectives lend themselves to a scoping review approach.	4
Objectives	4	Provide an explicit statement of the questions and objectives being addressed with reference to their key elements (e.g., population or participants, concepts, and context) or other relevant key elements used to conceptualize the review questions and/or objectives.	4
**METHODS**			
Protocol and registration	5	Indicate whether a review protocol exists; state if and where it can be accessed (e.g., a Web address); and if available, provide registration information, including the registration number.	5
Eligibility criteria	6	Specify characteristics of the sources of evidence used as eligibility criteria (e.g., years considered, language, and publication status), and provide a rationale.	5
Information sources*	7	Describe all information sources in the search (e.g., databases with dates of coverage and contact with authors to identify additional sources), as well as the date the most recent search was executed.	5
Search	8	Present the full electronic search strategy for at least 1 database, including any limits used, such that it could be repeated.	17
Selection of sources of evidence†	9	State the process for selecting sources of evidence (i.e., screening and eligibility) included in the scoping review.	5
Data charting process‡	10	Describe the methods of charting data from the included sources of evidence (e.g., calibrated forms or forms that have been tested by the team before their use, and whether data charting was done independently or in duplicate) and any processes for obtaining and confirming data from investigators.	5
Data items	11	List and define all variables for which data were sought and any assumptions and simplifications made.	6
Critical appraisal of individual sources of evidence§	12	If done, provide a rationale for conducting a critical appraisal of included sources of evidence; describe the methods used and how this information was used in any data synthesis (if appropriate).	N/A

St. Michael's
Inspired Care. Inspiring Science.

For peer review only - http://bmjopen.bmj.com/site/about/guidelines.xhtml

SECTION	ITEM	PRISMA-ScR CHECKLIST ITEM	REPORTED ON PAGE #
Synthesis of results	13	Describe the methods of handling and summarizing the data that were charted.	6
**RESULTS**			
Selection of sources of evidence	14	Give numbers of sources of evidence screened, assessed for eligibility, and included in the review, with reasons for exclusions at each stage, ideally using a flow diagram.	6
Characteristics of sources of evidence	15	For each source of evidence, present characteristics for which data were charted and provide the citations.	Table 1
Critical appraisal within sources of evidence	16	If done, present data on critical appraisal of included sources of evidence (see item 12).	N/A
Results of individual sources of evidence	17	For each included source of evidence, present the relevant data that were charted that relate to the review questions and objectives.	Table 1
Synthesis of results	18	Summarize and/or present the charting results as they relate to the review questions and objectives.	Table 1
**DISCUSSION**			
Summary of evidence	19	Summarize the main results (including an overview of concepts, themes, and types of evidence available), link to the review questions and objectives, and consider the relevance to key groups.	9
Limitations	20	Discuss the limitations of the scoping review process.	10
Conclusions	21	Provide a general interpretation of the results with respect to the review questions and objectives, as well as potential implications and/or next steps.	10
**FUNDING**			
Funding	22	Describe sources of funding for the included sources of evidence, as well as sources of funding for the scoping review. Describe the role of the funders of the scoping review.	11

JBI = Joanna Briggs Institute; PRISMA-ScR = Preferred Reporting Items for Systematic reviews and Meta-Analyses extension for Scoping Reviews.
* Where *sources of evidence* (see second footnote) are compiled from, such as bibliographic databases, social media platforms, and Web sites.
† A more inclusive/heterogeneous term used to account for the different types of evidence or data sources (e.g., quantitative and/or qualitative research, expert opinion, and policy documents) that may be eligible in a scoping review as opposed to only studies. This is not to be confused with *information sources* (see first footnote).
‡ The frameworks by Arksey and O'Malley (6) and Levac and colleagues (7) and the JBI guidance (4, 5) refer to the process of data extraction in a scoping review as data charting.
§ The process of systematically examining research evidence to assess its validity, results, and relevance before using it to inform a decision. This term is used for items 12 and 19 instead of "risk of bias" (which is more applicable to systematic reviews of interventions) to include and acknowledge the various sources of evidence that may be used in a scoping review (e.g., quantitative and/or qualitative research, expert opinion, and policy document).

From: Tricco AC, Lillie E, Zarin W, O'Brien KK, Colquhoun H, Levac D, et al. PRISMA Extension for Scoping Reviews (PRISMAScR): Checklist and Explanation. Ann Intern Med. 2018;169:467–473. doi: 10.7326/M18-0850.

How do children’s hospitals address health inequalities: a grey literature scoping review

Journal:	*BMJ Open*
Manuscript ID	bmjopen-2023-079744.R2
Article Type:	Original research
Date Submitted by the Author:	15-Dec-2023
Complete List of Authors:	Brennan, Louise; Lancaster University, Medical School; St Helens and Knowsley Teaching Hospitals NHS Trust<br>Stres, Dora Pestotnik; Lancaster University, Lancaster Medical School<br>Egboko, Fiona; Lancaster University, Lancaster Medical School<br>Patel, Pallavi; Lancaster University, Lancaster Medical School; St Helens and Knowsley Teaching Hospitals NHS Trust,<br>Broad, Eleanor; Dumfries and Galloway Royal Infirmary<br>Brewster, Liz; Lancaster University, Lancaster Medical School<br>Lunn, Judith; Lancaster University, Lancaster Medical School<br>Isba, Rachel; Lancaster University, Lancaster Medical School; Alder Hey Children's NHS Foundation Trust
**Primary Subject Heading**:	Public health
Secondary Subject Heading:	Paediatrics
Keywords:	Health Equity, PAEDIATRICS, PUBLIC HEALTH

SCHOLARONE™ Manuscripts

I, the Submitting Author has the right to grant and does grant on behalf of all authors of the Work (as defined in the below author licence), an exclusive licence and/or a non-exclusive licence for contributions from authors who are: i) UK Crown employees; ii) where BMJ has agreed a CC-BY licence shall apply, and/or iii) in accordance with the terms applicable for US Federal Government officers or employees acting as part of their official duties; on a worldwide, perpetual, irrevocable, royalty-free basis to BMJ Publishing Group Ltd ("BMJ") its licensees and where the relevant Journal is co-owned by BMJ to the co-owners of the Journal, to publish the Work in this journal and any other BMJ products and to exploit all rights, as set out in our licence.

The Submitting Author accepts and understands that any supply made under these terms is made by BMJ to the Submitting Author unless you are acting as an employee on behalf of your employer or a postgraduate student of an affiliated institution which is paying any applicable article publishing charge ("APC") for Open Access articles. Where the Submitting Author wishes to make the Work available on an Open Access basis (and intends to pay the relevant APC), the terms of reuse of such Open Access shall be governed by a Creative Commons licence – details of these licences and which Creative Commons licence will apply to this Work are set out in our licence referred to above.

Other than as permitted in any relevant BMJ Author's Self Archiving Policies, I confirm this Work has not been accepted for publication elsewhere, is not being considered for publication elsewhere and does not duplicate material already published. I confirm all authors consent to publication of this Work and authorise the granting of this licence.

For peer review only

How do children's hospitals address health inequalities: a grey literature scoping review

Louise Brennan [1, 2] l.brennan2@lancaster.ac.uk

Dora Pestotnik Stres [1] d.pestotnikstres@lancaster.ac.uk

Fiona Egboko [1] f.egboko@lancaster.ac.uk

Pallavi Patel [1,2] pallavi.patel4@nhs.net

Eleanor Broad [3] eleanor.broad@doctors.org.uk

Liz Brewster [1] e.brewster@lancaster.ac.uk

Judith Lunn [1] j.lunn1@lancaster.ac.uk

Rachel Isba [1,4] rachel.isba@lancaster.ac.uk (corresponding author)

[1] Lancaster Medical School, Lancaster University, Lancaster, UK

[2] St Helens and Knowsley Teaching Hospitals NHS Trust, Prescot, UK

[3] Dumfries and Galloway Royal Infirmary, Cargenbridge, Dumfries, UK

[4] Alder Hey Children's NHS Foundation Trust, Liverpool, UK

For peer review only

ABSTRACT (299)

Objectives:

Health inequalities are systematic differences in health between people, that are avoidable and unfair. Globally, more political strategies are required to address health inequalities, which have increased since the global SARS-CoV-2/COVID-19 pandemic, with a disproportionate impact on children.

This scoping review aimed to identify and collate information on how hospitals around the world that deliver care to children have addressed health inequalities.

Design:

Scoping review focussed solely on grey literature.

Eligibility criteria for selecting studies: Following Joanna Briggs Institute guidelines, a four-step approach to identifying literature was adopted.

Data sources: Overton, Open Grey, Openmd, tripdatabase, duckduckgo, Google, targeted websites and children's hospital websites were searched during March 2023 for items published since 2010.

Data extraction and synthesis: Retrieved items were screened against clear inclusion and exclusion criteria before data were extracted by two independent reviewers using a data extraction tool. Studies were tabulated by hospital. Meta-analysis was not conducted due to the varied nature of studies and approaches.

Results:

Our study identified 26 approaches to reduction of health inequalities, from 17 children's hospitals. Approaches were categorised based on their size and scope. Seven approaches were defined as macro, including hospital-wide inequality strategies. Ten approaches were classed as meso, including establishment of new departments and research centres. Micro approaches (n=9) included one-off projects or interventions offered to specific groups/ services. Almost half of the reported approaches did not discuss evaluation of impact.

Conclusions:

Children's hospitals provide a suitable location to conduct public health interventions. This scoping review provides examples of approaches on three scales delivered at hospitals across high-income countries. Hospitals with the most comprehensive and extensive range of approaches employed dedicated staff within the hospital and community. This review indicates the value of recruitment of both public health-trained staff as well as culturally-similar staff to deliver community-based interventions.

For peer review only

Strengths and limitations:

- Unlike traditional scoping reviews of peer-reviewed literature, this review focusses solely on grey literature.
- An extensive search of online grey literature databases, targeted websites and children's hospitals webpages was conducted.
- Grey literature may be more appropriate for areas of rapidly changing research such as new approaches to addressing health inequalities.
- Items were only included if available in English or translatable through online apps.

Key words:

Health equity, paediatrics, public health

Word Count: 3,833

For peer review only

INTRODUCTION

Health inequalities are systematic differences in health between people, that are avoidable and unfair.[1] Whilst most-commonly researched in relation to socioeconomic deprivation, [2, 3, 4, 5] the links between health inequalities and other types of social inequality such as disability,[6] race,[7] religious beliefs [8] and sexual orientation [9] are also well-evidenced.

Health inequalities are evident between and within countries across the world. Two examples of this are life expectancy and under-5s mortality rates. In sub-Saharan Africa, children are 14 times more likely to die before the age of 5 than in the rest of the world.[10] In India, the under-5 mortality rate was 106 per 1000 births to mothers with no education, compared with 49 per 1000 births to mothers with secondary or higher education.[11] In the UK, life expectancy is also socially patterned, with, for example, people residing in the most deprived areas of England living an average of 14 years less than those in the least deprived areas.[12]

Disparities in health outcomes are also evident globally. Research conducted in the USA found that rates of low birthweight were three times higher amongst babies born to Black mothers with no college education, compared with babies born to white mothers with a college education.[13] In the UK, children living in deprived areas are more likely to suffer from conditions such as tooth decay, asthma, diabetes and obesity.[14]

However, health inequalities are not just measured in life expectancy and health outcomes; patterns in accessing care are also correlated to socioeconomic status. In New Zealand, Māori young people are the least likely to be enrolled with a Primary Health Care provider.[15] In the UK, children from the most deprived areas are less likely to be brought to outpatient appointments,[16] but more likely to attend the Emergency Department, than those living in the least deprived areas.[17, 18]

Whilst the World Health Organization (WHO) has promoted the reduction of health inequalities as a global priority since its inaugural World Health Report in 1995,[19] political strategies to address the social determinants of health are still required in both high-, middle- and low-income countries.[20] In the UK, the National Health Service (NHS; a government-funded, free at the point of access, universal medical and healthcare service) has a legal duty to address health inequalities,[21] and this was made a key commitment in the *NHS Long Term Plan.*[22] However, the SARS-CoV-2/COVID-19 pandemic has widened health inequalities [23] and critics have argued that the absence of a national health inequalities strategy has led to local healthcare systems – including hospitals – struggling to develop their own approach to addressing health inequalities.[24]

Recognising the disproportionately negative impact of the pandemic on children,[25, 26] and lack of central steer on health inequalities in children and young people (CYP) in the UK [27, 28] and other countries,[29] this scoping review aimed to identify and collate information on how hospitals around the world that deliver care to children have addressed health inequalities. The findings of this scoping review are intended to be used to inform children's hospitals on approaches, outcomes and experiences based on previous work, to influence future interventions and policy.

Review Questions

1. What approaches do hospitals take to address health inequalities in children and young people?

2. What health inequalities do the approaches focus on?

3. How is effectiveness measured and demonstrated?

For peer review only

METHODS

As presented in the published protocol,[30] this scoping review focussed solely on grey literature due to the appropriateness of that approach for complex, multistakeholder interventions that lack robust data and predictability.[31] Grey literature is also more appropriate for topics that are under current review and moving at a fast pace, as is the case with interventions to address health inequalities. Furthermore, as hospitals and clinicians often have less capacity to publish academic literature in peer-reviewed journals, this methodology was deemed more appropriate as it allowed for a deeper, more systematic search of the internet and hospital websites.

Inclusion and exclusion criteria

As detailed in the protocol, items were included if initiated by a hospital, published in English, post-2010, focussed on children and with a clear aim to address health inequalities. Items were excluded if they did not explicitly state reducing health inequalities in their aim/ focus, and if the item did not describe an approach. Whole hospital approaches (including adults) and antenatal and prenatal services were also excluded.

Search Strategy

During January 2023, the online databases Overton, Open Grey, Openmd and tripdatabase were searched using the search terms outlined in the protocol. Secondly, search engines duckduckgo (allowing searches unaffected by previous search histories) and Google (using the anonymised search function) were searched using the search terms detailed in Appendix 1. The first 10 pages of returns were screened, unless items appeared to have continuing relevancy past 10 pages. Thirdly, targeted websites of the American Public Health Association (APHA), WHO, NHS England, the UK's Office for Health Improvement and Disparities (OHID) and the Health Management Information Consortium (HMIC) were explored using their search functions.

Fourth, as an add-on to the original search strategy, a list of worldwide children's hospitals was identified through Wikipedia (a free online, publicly editable encyclopaedia).[32] The authors searched the full list of children's hospital's websites individually, using individual website search bars and key terms. Non-English websites were translated using the Google Translate function.

Finally, snowballing was conducted at all stages of the search, where additional resources are identified through citations or weblinks.[33]

The full search strategy and terms is detailed in Appendix 1.

Study selection and data extraction

Searches and results were managed using Microsoft Excel. All items were screened based on their title (and where necessary associated text). Items deemed relevant were recorded by saving their website link. All items retrieved in this title screening stage were then screened in full against the inclusion and exclusion criteria by two independent reviewers. Any uncertainties or disagreements were first discussed between the two reviewers and then resolved by a third reviewer (RI). Data extraction was carried out by two separate reviewers, using a data extraction form developed using JBI guidelines [34] (Appendix 2). Where more than one approach was identified from the same hospital, they were classed as separate (and classed as a unique item) if they had separate aims. In some cases, multiple records were identified from one hospital, however they were all relating to the same aim/ approach (e.g. items under one strategy), and in these cases, records were combined into one entry.

For peer review only

Data synthesis

Following data extraction, studies were tabulated by hospital. Key details of the approach and its associated resources, setting and other key details were also presented alongside any outcome data. Due to the varied nature of studies and approaches no meta-analysis was conducted. Quality assessment was not undertaken due to the grey literature focus and lack of reporting consistency.

Patients and public involvement

No patients or public were involved.

RESULTS

Search results

In total, 2,752 results were returned from our database searches (identification stage). Of these, 2,585 were deemed inappropriate for inclusion, leaving 167 records for full text screening against the inclusion and exclusion criteria. During full text screening a further 18 records were identified through snowballing. Forty-five records were identified as duplicates and excluded. Items were then excluded if they did not meet the inclusion criteria (n=108). Six records were excluded after discussion with a third independent reviewer as consensus could not be met between the initial two reviewers, leading to 115 exclusions in total. Data were extracted from 26 texts (Figure 1).

General characteristics of approaches

Articles included in the results were published online between 2017 and the search date in January 2023. The approaches were from five high-income countries (United States of America, Australia, Canada, UK and New Zealand). Seventeen separate hospitals were included in the results. Boston Children's Hospital initiated five of the included approaches, Children's Hospital of Philadelphia (CHOP) initiated three separate approaches, with The Hospital for Sick Children Toronto (SickKids) and Sheffield Children's Hospital each initiating two; the remainder of hospitals had one record each. A summary of the included approaches is presented in supplementary Table 1. Whilst our protocol intended to investigate all hospitals providing care to children, the majority of our results originated from stand-alone children's hospitals.

Categorisation of approaches

Approaches were categorised into three themes based on the size and reach of the approach they were describing and are detailed below. 'Macro' approaches were organisation-wide, impacting the activity of the whole children's hospital, e.g. a formally-stated strategy. The approaches were classified as 'meso' if they involved setting up a new department or would change the activity of more than one department or service. 'Micro' approaches were single interventions, or projects that were short term or small in scale, e.g. reducing missed outpatient appointments. Almost half of the total 26 approaches (n=12) aimed to address a broad range of inequalities for the whole paediatric population. Meso or micro level approaches were more likely to focus on specific inequalities (e.g. financial hardship [35, 36]) or inequalities in access or outcomes for specific groups (e.g. Aboriginal children [37] First Nation Children [38] or refugees and asylum seekers).[39]

Macro

Seven approaches were categorised as macro. Four of these were hospital-wide strategies, specifically aimed to address: health equity (Australia);[37] equity, diversity and inclusion (EDI)

For peer review only

(Canada);[40] health inequalities within an innovation strategy (UK);[41] and inclusion of health inequalities in a clinical strategy (UK).[42]

Two hospitals had an 'ethos of equity' – in Boston this was formalised through a declaration and goals.[43] At the Starship Hospital in New Zealand this was not formalised in a strategy but is conceptualised as a commitment in all that they do.[44]

The final macro approach to health inequalities began as a micro approach but has since become hospital-wide and replicated across the UK. The *rainbow badge initiative* is for staff, who, when signing up to wear a rainbow badge receive information and training about the challenges that people who identify as LGBT+ can face when accessing healthcare and what they can do to support them.[45]

Meso.

Ten approaches were characterised as meso. Four of the US hospitals had established a centre aiming to address equity with dedicated staff. In Boston, the *Office for Health Equity and Inclusion*, aimed to coordinate EDI initiatives across the whole hospital [46] and the *Fenwick Institute*, focused on research into paediatric health equity.[47] The latter has provided funding for inequalities research and delivered research projects, including investigation into missed appointments and effective interventions to recruit diverse families. CHOP also established a *Center for Health Equity* [48] and a research *Policy Lab*, which aimed to advance health equity through a research lens.[49] The Cincinnati Children's Hospital's *Center for Child Equity* aimed to build equitable care capabilities in staff,[50] and Seattle Children's Hospital's *Center for Diversity and Health Equity* focused on patient and family education programmes.[51] These latter hospitals have no separate research centre but include research in their equity approach.

Alongside their centre, CHOP had also established the *Healthier Together* partnership, together with community groups and non-profit government agencies to invest money into the community, families and individuals. Interventions included distribution of healthy meals, home renovation and financial counselling.[52] In Toronto, Canada, the *Family Navigation Hub* was established following the development of a social needs screening tool.[53] The hub aimed to help families to access and close the gaps around availability of housing, food, childcare, respite and community connection.[49]

Several meso level approaches focused on specific patient groups. An *Indigenous Liaison Service,* established in Queensland Children's Hospital, Australia, targeted support, advocacy and provided assistance to indigenous people accessing health and social services.[54] Using the nationally-established *Jordan's Principle*, which ensures that First Nations children living in Canada can access services when/ as they are needed, the Health Sciences Centre Children's Hospital (Winnipeg) employed a *Jordan's Principle* co-ordinator to assist First Nations children and young people to access care at their facility.[38] Similarly, in Gateshead, England, a service had been developed to provide a single point of access for refugee and asylum-seeking children and families. This aimed to reduce barriers to healthcare by connecting families to the right health, statutory or voluntary service leading to referrals for oral health, obesity, immunisations and the tuberculosis (TB) service.[39]

Micro

Nine approaches were categorised as 'micro' meaning they were projects or interventions used across one service/ department rather than at a larger scale. Three projects originated in England. The *Families Matter* project in Sheffield aimed to help parents with financial cost of staying in

For peer review only

hospital, and provided resources to allow them to maintain basic hygiene and needs, including sanitary products, food and additional support such as signposting to additional providers of financial advice. At the time of data extraction, the hospital was piloting a free breakfast for all parents staying with their child. Feedback from staff and families was positive, with staff reporting that caregivers appeared less stressed as a result of the enhanced offers of support.[36] In Birmingham, a pilot project was running to offer free transport to families at risk of non-attendance at the hospital. Pilot data showed that 90% of the study group took up the offer of help and 140 appointments were relocated during the eight weeks.[35] Thirdly, Newcastle upon Tyne Hospitals NHS Trust trialled assigning a health contact, who conducted health assessments for school-aged children living in homeless accommodation. In the three-month pilot, 17 of the 20 children assigned a health contact had health assessments, leading to support with oral health, ophthalmology treatment, referrals for immunisations and access to the TB service.[39] Data collection and analysis was the focus of two projects that fell into the micro approach category. During the SARS-CoV-2/COVID-19 pandemic, Boston Children's Hospital analysed data on the impact of the pandemic on communities of colour and published the findings through Twitter. The posts reached more than 100,000 people, raising awareness and reaching state legislators.[55] SickKids, Canada developed a paediatric data tool and health equity survey as part of a data collection pilot. Clerical staff collected data and 3,000 staff received training on culturally-competent care. Parents and carers reported satisfaction with the data tool and registration was identified as the optimal point in the healthcare visit to collect sociodemographic data.[56]

Community outreach projects were common in the approaches we found. In Canada, led by British Columbia Children's Hospital, the *RICHER* (Responsive Intersectoral Child and Community Health Education and Research) programme was designed to address disparities in access to primary care for children, youth and families in Vancouver's inner cities.[57] A project run by Sydney Children's Hospital aimed to improve access and equity for children with complex needs using technology and those living in rural districts. They demonstrated a 40% reduction in Emergency Department visits and a 42% decrease in day admissions, saving more than 50,000km travel for families.[58] In Boston, children from low-income families of colour, with asthma, were the target population for case management and home visits by a community health worker. An 82% reduction in asthma-related hospitalisation rates was reported in the 2,491 enrolled patients. Other outcomes included 55% reduction in lost workdays for parents and 45% reduction in missed school days.[59]

Evaluation tools

Nearly half (n=12) of the approaches reported no evaluation/ measure of success in their communication. Those hospitals reporting their outcomes used a range of different measures, with uptake/ number of people involved being the most common (n=10). Several approaches used caregiver satisfaction (n=3), with qualitative findings providing powerful quotes from service users.[53] Other less common measures were number of staff trained (n=2), accuracy of prediction tool (n=1), and money invested in the wider community by the hospital (n=1). Those hospitals that attempted to quantify their impact on health inequalities reported outcomes such as homes renovated [52] or modified,[44] change in research study uptake in diverse families,[49] reductions in hospitalisation, missed days from work and lost days at school,[59] reduction in emergency admissions and miles travelled.[58]

Staffing/ resources

Approaches varied in their approaches to staffing. Shorter-term projects used existing staff such as clerical staff for data collection,[56, 35] whereas new centres/ dedicated departments reported new

For peer review only

staff structures with dedicated teams and additional infrastructure. For community outreach or targeted interventions with specific communities, positive outcomes were attributed to culturally-similar staffing.[44, 37, 54, 38] Taking the service out into the community was also reported to improve outcomes for traditionally-marginalised patients, and in these cases, community health workers were often utilised. Approaches did not specify the cost of establishing and delivering their approach and therefore data could not be compared.

DISCUSSION

In this paper we have provided a synthesis of the approaches taken by children's hospitals in addressing health inequalities experienced by children and young people. Our search of grey literature aimed to be a comprehensive way to investigate unpublished literature produced by hospitals and their partners. Whilst our search aimed to investigate all hospitals providing care for children, the results originated solely from children's hospitals.

Our study identified 26 approaches to reduction of health inequalities, from 16 children's hospitals, all in high-income countries. Approaches have been categorised according to their depth and breadth of reach, from macro whole-organisation approaches, down to small micro pilot projects. The majority of approaches were categorised as meso and were commonly newly-established departments or centres dedicated to addressing equity and research into this area.

Twelve approaches had no specific inequality as their focus, instead taking a broad approach to addressing equity. Seven hospitals addressed inequalities in access to healthcare, a further four focussed on social determinants of health and the remaining three addressed health outcomes for specific groups.

Almost half of the approaches failed to report outcomes or their methods of evaluation/ assessment and very few reported costs. Although it is notoriously difficult to demonstrate the impact of public health interventions,[60] there is still a recognised need to build an evidence base around interventions that aim reduce health inequalities and improve population health.[61] It is therefore important for hospitals to better evaluate and communicate their activities and findings in the future.

Few publications relating to hospital approaches to address health inequalities in the academic literature led to the refocus of this scoping review to grey literature only. Previous research has suggested that lack of time is the most common reason for non-publication of medical and health related studies;[62] however, this scoping review highlights the merits of publishing information on websites and other non-peer reviewed repositories of information, whilst also supporting the utilisation of grey literature for reviews of hospital-based interventions.

Hospitals that did evaluate their approaches varied in their evaluation methods. At the most basic level, hospitals counted numbers involved and uptake of interventions. More complex evaluations looked at changes in missed appointments and emergency admissions by demographics such as ethnicity. Other hospitals considered missed school days and days from work for parents and carers. As this is a scoping review, an assessment of study quality was not undertaken, and findings should therefore be interpreted with care.

Hospitals with dedicated centres and a team of staff reported a greater number of more comprehensive outcomes. Hospitals with a dedicated health inequalities focussed strategy, or health inequalities within their wider strategies were also more likely to have an increased level of activity. However, strategy did not always equate to outcomes, and it is therefore important for hospitals to

For peer review only

consolidate strategy with projects and appropriate staff resources. Projects involving culturally-similar staff or dedicated community teams reported success in reaching historically marginalised communities.

There are several reasons for the possible exclusion of approaches from middle- and low-income countries. Firstly, due to time and resource constraints our search was limited to materials available in the English language. Translate apps were utilised where possible as all searching was completed using the internet, nevertheless this possibly restricted findings from some sites where translation was not accurate enough to identify alternative terms for our keywords. Secondly, it is highly probable that the terminology used to describe health inequalities e.g. disparities and inequities, are not universally adopted around the world, and approaches that were relevant were excluded due to the way the intervention was described. This was confirmed during screening of worldwide children's hospitals websites, where several potentially relevant approaches aimed to provide culturally-appropriate care rather than equity in health, and were therefore excluded. Hence, lack of reporting on approaches does not imply lack of activity in this review. Thirdly the exclusion of hospital-wide, antenatal, prenatal and adult focussed approaches may have led to the exclusion of early years and some family-centred approaches to addressing health inequalities. Similarly, the exclusion of whole-hospital strategies that include adults may explain the results only including children's hospitals. It is possible that hospital-wide strategies existed in non-freestanding children's hospitals, that also addressed inequities in children, however these were not eligible for inclusion in this review.

The context of healthcare systems is important to consider when interpreting these results. The majority of approaches originated from US hospitals, where healthcare is not universal and hospital activity could be driven by factors different to those where healthcare is free at the point of access. Furthermore, since the Affordable Care Act of 2010, American non-profit hospitals are mandated to undertake a community needs assessment and documentation of how they are addressing needs every three years.[63] Similarly, in England there has been a national drive to address health inequalities due to their inclusion in the NHS Long Term Plan [22] and the current 'cost of living crisis' has impacted access to care [64] and fuelled activity to address the cost of hospital appointments for patients.

CONCLUSIONS AND RECOMMENDATIONS

Children's hospitals and hospitals that care for children provide a suitable location to conduct public health interventions. This scoping review has explored and summarised the approaches that some children's hospitals in high-income countries have taken to address health inequalities. Several children's hospitals (mainly in the US) have advanced equity programmes of work, but this may be driven by the regulatory environment.

There was a lack of robust evaluation of the approaches, despite public health interventions requiring appropriate evaluation to enable evidence-based practice.

The lack of published literature on hospital-led approached to health inequalities highlights the need for hospitals to promote the work that they are doing, so areas of good practice and learning can be shared and implemented. As demonstrated here, this could be via hospital websites or through networks. The dearth of peer-reviewed articles also highlights the relevance of grey literature for hospital-based research such as this. The development of further methodological guidance on grey literature-focused scoping and systematic reviews is warranted.

For peer review only

The findings of this review originated from a small number of high-income countries. It is possible that relevant approaches are being used elsewhere but not being published, and/ or are not labelled as health inequalities. Further research into this would be warranted to complete a broader summary of approaches.

Hospitals with the most comprehensive and extensive range of approaches employ dedicated staff within the hospital and community. This review therefore provides evidence for the value of the recruitment of both public health trained staff as well as culturally-similar staff to deliver community-based interventions.

Research Ethics statement

This study does not involve human participants

Contributorship statement

Louise Brennan, Rachel Isba, Liz Brewster and Judith Lunn conceptualised the work. Louise Brennan, Dora Pestotnik Stres and Fiona Egboko planned the study. Dora Pestotnik Stres and Louise Brennan designed the data extraction tool. Louise Brennan and Pallavi Patel ran the database and website searches. Louise Brennan, Dora Pestotnik Stres, Fiona Egboko, Pallavi Patel and Eleanor Broad searched the children's hospital websites and were also all responsible for screening all retrieved items. Louise Brennan and Dora Pestotnik Stres independently extracted data. Discrepancies were discussed with Rachel Isba. Themes were discussed and developed by Louise Brennan, Dora Pestotnik Stres, Liz Brewster and Judith Lunn. Louise Brennan prepared the original draft. Louise Brennan, Dora Pestotnik Stres, Fiona Egboko, Pallavi Patel and Eleanor Broad designed and populated the supplementary table. Rachel Isba and Liz Brewster critically reviewed the work. All authors edited and approved the final version and are accountable for the accuracy and integrity of the work.

Competing interests

The authors declare that they have no known competing financial interests or personal relationships that could have appeared to influence the work reported in this paper.

Funding

This work was supported by the National Paediatric Accelerator Programme, now the Children's Hospital Alliance, contracted by Sheffield Children's Hospital, grant number SCH5628.

Data sharing statement

No additional data available.

Acknowledgements

Thank you to Louise Speakman, Lancaster University who kindly advised on the original search strategy.

For peer review only

For peer review only

References

1 Williams E, Buck D, Babalola G, et al. What are health inequalities?. *The King's Fund* 2022.

2 Marmot M. Fair society, healthy lives. *Fair society, healthy lives* 2013:1-74.

3 Graham H. Unequal lives: McGraw-Hill Education (UK) 2007.

4 Acheson D. Independent Inquiry into Inequalities in Health. *Science* 1998;10:217-33.

5 Patel JA, Nielsen F, Badiani AA, et al. Poverty, inequality and COVID-19: the forgotten vulnerable. *Public Health* 2020;183:110.

6 Emerson E, Baines S, Allerton L, et al. Health inequalities and people with learning disabilities in the UK 2011.

7 Elwell-Sutton T, Deeny S, Stafford M. Emerging findings on the impact of COVID-19 on black and minority ethnic people. *The Health Foundation* 2020.

8 Office for National Statistics. Religion and Health in England and Wales. 2020; Available at: https://www.ons.gov.uk/peoplepopulationandcommunity/culturalidentity/religion/articles/religionandhealthinenglandandwales/february2020. Accessed 30/06/2023.

9 Booker CL, Rieger G, Unger JB. Sexual orientation health inequality: evidence from understanding society, the UK longitudinal household study. *Prev Med* 2017;101:126-32.

10 World Health Organization. Health Inequalities and their causes. 2018; Available at: https://www.who.int/news-room/facts-in-pictures/detail/health-inequities-and-their-causes. Accessed 30/06/2023.

11 Balarajan Y, Selvaraj S, Subramanian SV. Health care and equity in India. *The Lancet* 2011;377:505-15.

12 Rashid T, Bennett JE, Paciorek CJ, et al. Life expectancy and risk of death in 6791 communities in England from 2002 to 2019: high-resolution spatiotemporal analysis of civil registration data. *The Lancet Public Health* 2021;6:e805-16.

13 Pollock EA, Gennuso KP, Givens ML, et al. Trends in infants born at low birthweight and disparities by maternal race and education from 2003 to 2018 in the United States. *BMC Public Health* 2021;21:1117.

14 Wickham S, Anwar E, Barr B, et al. Poverty and child health in the UK: using evidence for action. *Arch Dis Child* 2016;101:759-66.

15 Irurzun-Lopez M, Jeffreys M, Cumming J. The enrolment gap: who is not enrolling with primary health organizations in Aotearoa New Zealand and what are the implications? An exploration of 2015–2019 administrative data. *International journal for equity in health* 2021;20:1-11.

16 French LR, Turner KM, Morley H, et al. Characteristics of children who do not attend their hospital appointments, and GPs' response: a mixed methods study in primary and secondary care. *British Journal of General Practice* 2017;67:e483-9.

For peer review only

17 Jarvis S, Livingston J, Childs A, et al. Outpatient appointment non-attendance and unplanned health care for children and young people with neurological conditions: a retrospective cohort study. *Developmental Medicine & Child Neurology* 2019;61:840-6.

18 Scantlebury R, Rowlands G, Durbaba S, et al. Socioeconomic deprivation and accident and emergency attendances: cross-sectional analysis of general practices in England. *British Journal of General Practice* 2015;65:e649-54.

19 King NB, Harper S, Young ME. Who cares about health inequalities? Cross-country evidence from the World Health Survey. *Health Policy Plan* 2013;28:558-71.

20 Rebouças P, Falcão IR, Barreto ML. Social inequalities and their impact on children's health: a current and global perspective. *J Pediatr* 2022;98:55-65.

21 Equality Act. Equality Act 2010.

22 NHS. The NHS long term plan. *NHS* 2019.

23 Buck D, Lewis T. NHS England and tackling inequalities: times are changing. *The Kings Fund* 2021.

24 Ford J, Sowden S, Olivera J, et al. Transforming health systems to reduce health inequalities. *Future Healthcare Journal* 2021;8:e204.

25 McLellan A, Abbasi K. The NHS is not living with covid, it's dying from it. *BMJ* 2022;378.

26 Pickett K, Taylor-Robinson D, Erlam J. Child of the North: Building a fairer future after COVID-19 2021.

27 Bradley SH. The ethics and politics of addressing health inequalities. *Clinical Medicine* 2021;21:147.

28 Choudry M. Tackling health inequalities and promoting prevention: If not now, then when?. *Future Healthcare Journal* 2021;8:4.

29 Dickman SL, Himmelstein DU, Woolhandler S. Inequality and the health-care system in the USA. *The Lancet* 2017;389:1431-41.

30 Brennan L, Brewster L, Lunn J, et al. How do hospitals address health inequalities experienced by children and young people: a grey literature scoping review protocol. *BMJ open* 2023;13:e071682.

31 Tyndall J, Merlin T, Lockwood C. Impact of findings from grey literature on the outcomes of systematic reviews on interventions to prevent obesity among children: a systematic review. *JBI Evidence Synthesis* 2012;10:1-14.

32 Wikipedia. List of Children's hospitals. 2023; Available at: https://en.wikipedia.org/wiki/List_of_children%27s_hospitals. Accessed 18/03/2023.

33 Sayers A. Tips and tricks in performing a systematic review. *British Journal of General Practice* 2008;58:136.

34 Peters MD, Godfrey CM, Khalil H, et al. Guidance for conducting systematic scoping reviews. *JBI Evidence Implementation* 2015;13:141-6.

35 Birmingham Women's and Children's NHS Foundation Trust. New pilot project reduces 'was not brought' rates for children with hospital appointments. 2023; Available at: https://bwc.nhs.uk/news/new-pilot-project-reduces-was-not-brought-rates-for-children-with-hospital-appointments-7946. Accessed 18/03/2023.

36 Sheffield Children's Hospital. Reducing the financial cost – Families Matter. 2022; Available at: https://www.sheffieldchildrens.nhs.uk/patients-and-parents/patienthq/families-matter/. Accessed 18/03/2023.

37 The Sydney Children's Network. Transforming health for a more equitable future. Available at: https://www.schn.health.nsw.gov.au/news/articles/2022/03/transforming-health-for-a-more-equitable-future. Accessed 18/03/2023.

38 Southern Chiefs' Organization. SCO adds Jordan's Principle Coordinator at HSC Winnipeg Children's Hospital. 2022; Available at: https://hsc.mb.ca/wp-content/uploads/SCO-Media-Release-SCO-Adds-JP-Coordinator-at-HSC.pdf.

39 Child Health and Wellbeing Network. Addressing Child Health Inequalities in the North East and North Cumbria - Tackling health inequalities in school age children living in homeless accommodation. *North East and North Cumbria* 2022 doi:https://northeastnorthcumbria.nhs.uk/media/0ybppw33/addressing-child-health-inequalities-project-showcase.pdf.

40 SickKids. Equity, Diversity and Inclusion Strategy. 2022; Accessed 18/03/2023.

41 Alder Hey Children's NHS Foundation Trust. Alder Hey Innovation announces far-reaching strategy to address healthcare challenges faced by children and young people. 2023; Available at: https://www.alderhey.nhs.uk/alder-hey-innovation-announces-far-reaching-strategy-to-address-healthcare-challenges-faced-by-children-and-young-people/. Accessed 18/03/2023.

42 Sheffield Children's NHS Foundation Trust. A clinical strategy for a healthier future. 2022; Available at: https://www.sheffieldchildrens.nhs.uk/download/316/general/58220/a-clinical-strategy-for-a-healthier-future.pdf. Accessed 18/03/2023.

43 Boston Children's Hospital. About Us: Health Equity. 2023; Available at: https://www.childrenshospital.org/about-us/health-equity. Accessed 18/03/2023.

44 Starship. Equity. 2022; Available at: https://starship.org.nz/foundation/equity-ensuring-no-child-is-left-behind/. Accessed 18/03/2023.

45 Guy's and St Thomas' NHS Foundation Trust. NHS rainbow badges promote inclusion. 2019; Available at: https://www.evelinalondon.nhs.uk/about-us/news-events/2019-news/20190321-nhs-rainbow-badges-promote-inclusion.aspx. Accessed 18/03/2023.

46 Boston Children's Hospital. About Us: Office of Health Equity & Inclusion. 2023; Available at: https://www.childrenshospital.org/about-us/health-equity/office-health-equity-inclusion. Accessed 18/03/2023.

For peer review only

47 Boston Children's Hospital. The Sandra L. Fenwick Institute for Pediatric Health Equity and Inclusion. 2023; Available at: https://www.childrenshospital.org/about-us/health-equity/fenwick-institute. Accessed 18/03/2023.

48 Children's Hospital of Philadelphia. Center for Health Equity. Available at: https://www.chop.edu/centers-programs/center-health-equity. Accessed 18/03/2023.

49 Children's Hospital of Philadelphia. Policy Lab. Health Equity. 2022; Available at: https://policylab.chop.edu/our-research/health-equity. Accessed 18/03/2023.

50 Cincinnati Children's Hospital. Cincinnati Children’s new Center for Child Health Equity named for departing CEO Michael Fisher. 2021; Available at: https://www.cincinnatichildrens.org/news/release/2021/child-health-equity. Accessed 18/03/2023.

51 Seattle Children's Hospital. Center for Diversity and Health Equity. 2023; Available at: https://www.seattlechildrens.org/clinics/diversity-health-equity/. Accessed 18/03/2023.

52 Children's Hospital of Philadelphia. Healthier Together Initiative. 2022; Available at: https://www.chop.edu/centers-programs/healthier-together#parentHorizontalTab4. Accessed 18/03/2023.

53 Holland Bloorview Kids Rehabilitation Hospital. Addressing family needs through the power of community. 2023; Available at: https://hollandbloorview.ca/stories-news-events/news/addressing-family-needs-through-power-community.

54 Queensland Children's Hospital. Aboriginal and Torres Strait Island Support. 2022; Available at: https://www.sunshinecoast.health.qld.gov.au/patients-and-visitors/aboriginal-and-torres-strait-islander-support#section__aboriginal-and-torres-strait-islander-hospital-liaison-officers. Accessed 18/03/2023.

55 Boston Children's Hospital. COVID-19 exposed health inequities. These doctors let people know. 2021; Available at: https://answers.childrenshospital.org/covid-19-health-inequities-twitter/. Accessed 18/03/2023.

56 SickKids. Advancing Health Equity at the Hospital for Sick Children. 2019; Available at: https://www.health.belgium.be/sites/default/files/uploads/fields/fpshealth_theme_file/karmali_health_equity_brussels_nov2019.pdf. Accessed 18/03/2023.

57 British Columbia Children's Hospital. Social Pediatrics. 2023; Available at: http://www.bcchildrens.ca/our-services/clinics/social-pediatrics#RICHER--Initiative.

58 The Sydney Children's Hospitals Network. Priority Populations programs given multi-million dollar boost. 2021; Available at: https://www.schn.health.nsw.gov.au/news/articles/2021/10/priority-populations-programs-given-multi-million-dollar-boost.

59 Boston Children's Hospital. Community Asthma Initiative. 2023; Available at: https://www.childrenshospital.org/programs/community-asthma-initiative. Accessed 18/03/2023.

60 Petticrew M. Emerging challenges in evidence-based public health and how to address them. *Journal of Health Monitoring* 2020;5:7.

For peer review only - http://bmjopen.bmj.com/site/about/guidelines.xhtml

61 Klepac B, Krahe M, Spaaij R, et al. Increasing research evidence translation and utilisation to improve population health outcomes 2022.

62 Song F, Loke Y, Hooper L. Why are medical and health-related studies not being published? A systematic review of reasons given by investigators. *PloS one* 2014;9:e110418.

63 Wahowiak L. Community needs assessments leading to better outcomes: ACA requirement fortifying health. *The Nation's health* 2017:1-8.

64 Healthwatch. Cost of living: People are increasingly avoiding NHS appointments and prescriptions. 2023; Available at: https://www.healthwatch.co.uk/news/2023-01-09/cost-living-people-are-increasingly-avoiding-nhs-appointments-and-prescriptions#:~:text=The%20impact%20the%20cost%20of%20living%20crisis%20is,prescriptions%20and%20over-the-counter%20medications%20because%20of%20the%20costs.

Figure 1 – Flow diagram of searches and screening.

Table 1 – Summary of included approaches.

For peer review only

Figure 1 – Flow diagram of searches and screening

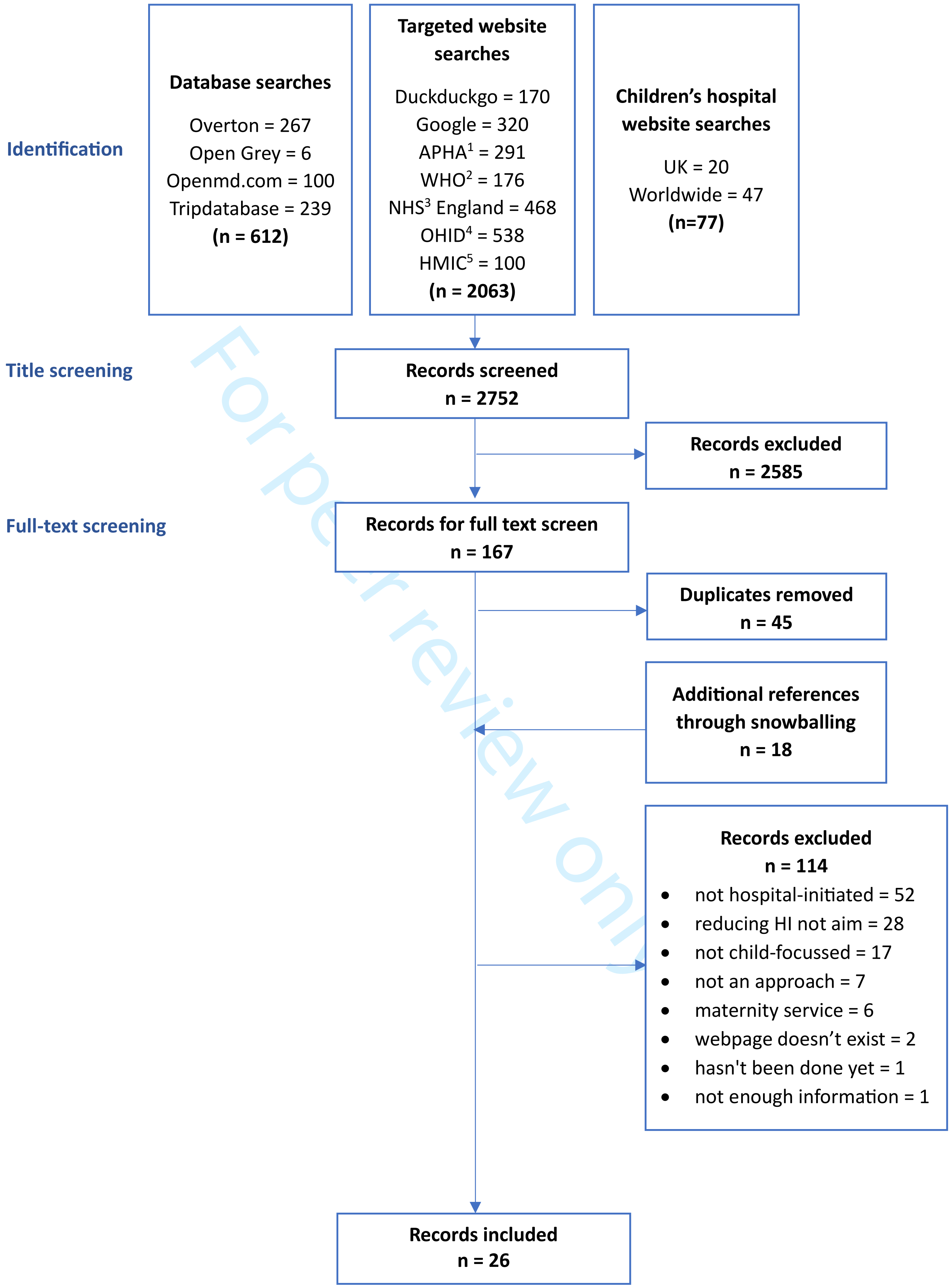

[1] American Public Health Association, [2] World Health Organization, [3] National Health Service, [4] Office for Health Improvement and Disparities, [5] Health Management Information Consortium

For peer review only

Table 1. Detailed summary of included approaches

In-text ref	Country	Hospital	Approach	Type	Details	Year	Inequity	Setting	Population	Outcomes reported	Staff/ resources involved	Other useful learning	Evaluation/ measure of success
35	England	Birmingham Women and Childrens Hospital	Intervention	Micro	Pilot project targeting families at risk of nonattendance - offered free transport to the hospital.	2023 Pilot	Social determinants of health	Hospital	Families in most deprived areas	90% of the study group took up the offer of help with travel and another 140 appointments that were reallocated.	Administration staff worked extra hours making phone calls, supported by Trust's interpreter team.	Staff also started to develop better relationships with the people they were contacting, helping to build trust and increase engagement with their local community.	Number of uptake, reduction in WNB.
36	England	Sheffield Children's Hospital	Intervention	Micro	Families Matter Project - aimed to provide parents with resources to allow them to maintain basic hygiene from the point of admission, ensure that no families go hungry by developing a food pledge and supporting families by signposting them to resources to support them financially alongside the development of access to Citizen's Advice booths.	2022	Social determinants of health	Hospital	All	Positive feedback from families on the provision of basic care resources, particularly sanitary products. Staff members have found that families are less stressed about how they will look after themselves when in patients, it has also been a positive for staff wellbeing in taking care of our families.	All staff	N/A	Qualitative feedback from parents.
37	Australia	Sydney Children's Hospital Network	Strategy	Macro	Strategy with interventions as a result such as projects/ increasing number of Aboriginal staff, coordinator role for Aboriginal Health Outcomes and Equity Project (aiming to ensure the constant delivery of high-quality, equitable services to Aboriginal children).	2018	General	Hospital and community	Aboriginal children	Aboriginal workforce has grown by 20 staff in clinical and non-clinical roles. Established new dashboard to look at real time data and if aboriginal patients have been seen by an Aboriginal health worker within 24 hours of admission.	All staff withing the hospital network, the Aboriginal Health Outcomes and Equity Project Manager, Aboriginal Health Workers	"The advantage of addressing Aboriginal health as part of a children's hospital network is that we have a wide range of services and, therefore, an opportunity to intervene early in the life course."	Not reported.
38	Canada	Health Sciences Centre Children's hospital Winnipeg	Intervention	Micro	Employed a 'Jordan's Principal Coordinator' - The Jordan's Principle Coordinator at HSC Children's Hospital will assist First Nation children and youth under 18 and their families in accessing programs, services, and supports to improve health, education, and social outcomes.	2022	Access	Hospital	First nation children	None reported	Coordinator and team	N/A	Not reported.
39	England	Newcastle Upon Tyne Hospitals NHS Trust	Intervention	Micro	Health contact for school-aged children living in homeless accommodation (previously only offered to <5 year olds). Children and parents are offered an appointment for a health assessment on two days per month, initially for a three-month pilot period.	2022	Access	Community	Children living in homeless accommodation	In the three-month trial period, 20 school age children were offered health assessments. 17 out of those 20 have had the health assessments. Ten children were supported with oral hygiene needs, with four referrals into treatment at acute level, six had vision needs and four were referred to Ophthalmology for treatment. Five had incomplete immunisations, three received referrals to tuberculosis service, and two had an increased BMI.	Two public health school nurses	The service has used this model of service delivery to support health assessments for asylum seekers locally, and all children under 19 have received health assessments prior to being rehoused. Staff visit the asylum-seeking families' temporary accommodation once per week. Also plan to offer the same whole family support to families in a domestic abuse refuge.	Number of children offered health assessments and number of health interactions that resulted from those (e.g. referrals to a certain service, immunisations).
39	England	Gateshead Health NHS Foundation Trust	Service	Meso	Single point of access for refugee and asylum seekers. Undertake a health needs assessment and remove barriers by connecting families directly to the right health, statutory or voluntary services.	2022	Access	Community	Refugees and Asylum Seekers	Over 140 children had comprehensive health assessments in a secondary care clinic, and dozens of families had similar assessments in a local asylum seeker accommodation through primary care. "Health needs that would have been unrecognised and unmet have been addressed, improving health outcomes for these families." Based on these data, a project spanning primary and secondary care, linking a wide range of other services, has been commissioned to further improve capacity and quality of this work.	Not specified	Engagement of the various stakeholders is critical for this approach to be successful.	Numbers of appointments.

For peer review only

Table 1. Detailed summary of included approaches

In-text ref	Country	Hospital	Approach	Type	Details	Year	Inequity	Setting	Population	Outcomes reported	Staff/ resources involved	Other useful learning	Evaluation/ measure of success
40	Canada	Hospital for Sick Children Toronto (SickKids)	Strategy	Macro	EDI strategy and indigenous health council aims to make care more equitable for indigenous people, ultimately striving to improve child health outcomes.	2020	General	Hospital and community	All	None reported.	Patients, EDI Committee	N/A	Measurement and evaluation of disparities in access to care; research and education to minimize variation .
41	England	Alder Hey, Liverpool	Strategy	Macro	Innovation strategy and associated projects e.g. analysis of 'was not brought' (WNB) children data to identify families at risk of nonattendance. Other innovation e.g. transparent masks	2022 pilot	General	Hospital	All	WNB brought project- AI- ability to calculate the likelihood of whether a child will come to their appointment or not, and so make an early intervention: piloted on 34 patients in general paediatrics, model has 80% accuracy.	Innovation team	Planning to share WNB project AI tool with other Trusts.	Success of tools e.g. accuracy of AI predictor.
42	England	Sheffield Children's Hospital	Strategy	Macro	Strategy seeks to minimise the impact of health inequalities on mental and physical health every time a family interacts with us to improve experience, access, and outcomes. Working with partners to address the root causes of inequalities and invest where the need is greatest.	2022	General	Hospital and advocating wider	All	None reported.	Not specified	N/A	Not reported.
43	USA	Boston Children's Hospital	Declaration and goals	Macro	To address racial injustice and health disparities, Boston Children's released a Declaration on Equity, Diversity, and Inclusivity. The declaration established six goals to elevate health equity as an enterprise priority, core to everything the hospital does.	2020	General	Hospital	All	Six goals committing to addressing inclusion, recruitment, structural racism, employee education, leading on elimination of and metrics to track health disparities. No other outcomes reported.	Chief Equity and Inclusion Officer	N/A	Not reported.
44	New Zealand	Starship Hospital, Auckland	Ethos	Macro	The Starship Foundation is committed to meeting children and their whānau where they are, ensuring all children in New Zealand have access to world-class healthcare regardless of their ethnicity, where they live, or their family circumstance, decreasing ethnic health disparities in Māori Children, seeking to improve the health journey for tamariki Māori and their whānau, to ultimately achieve equitable outcomes.	2019	General	Hospital and community	All	Online content resonates strongly with Māori and Pacific audiences, with 667,006 post engagements reflecting strong connection and cut-through, Healthy Homes supported 492 households and fitted 237 homes with thermal curtains.	All staff, Starship Child Health has now embedded a Māori Health Team	Produce clinical guidelines for other hospitals.	Yearly impact reports.
45	England	Evelina	Intervention	Macro	NHS Rainbow Badges are being worn by staff. When staff sign up to wear the badge they are provided with information about the challenges people who identify as LGBT+ can face accessing healthcare and what they can do to support them. Wearing the badge is a sign that the wearer is someone patients can talk to about issues of sexuality and gender identity.	2019	Access	Hospital	Young LGBT+	Almost a third of staff have signed up to wear and be trained. This has since been rolled out across NHS Trusts in the UK and an associated toolkit developed. In 2021 the NHS Rainbow badge moved from a purely visual symbol to also incorporating an assessment and accreditation mode for NHS Trust.	All staff	Rolled out across other Trusts in England.	Uptake.
46	USA	Boston Children's Hospital	Established department / centre	Meso	Office of Health Equity and Inclusion. Coordinates equity, diversity and inclusion initiatives across the hospital.	2017	General	Hospital	All	Initiatives such as professional development seminars and training, career advancement and pipeline programs, mentoring, research, development of metrics.	Led by Chief Equity and Inclusion Officer	Work in partnership with Harvard Medical School and other partners.	Not reported.
47	USA	Boston Children's Hospital	Established research centre/ portfolio	Meso	Institute for Paediatric Health Equity: focuses on 3 areas: health equity research, inclusion, and public policy. Ensuring equitable health care access, delivery, and outcomes for all children.	2021	General	Hospital	All	Research projects to recruit diverse families, studies investigating missed appointments and culturally effective interventions. Seed grants to fund inequalities research.	Led by Senior Vice President. Also, Chief Equity and Inclusion Officer, Paediatric Radiologist	N/A	Not reported.
48	USA	Children's Hospital of Philadelphia (CHOP)	Established department / centre	Meso	Established Center for health equity: 4 core workstreams: Community Translation, Clinical Quality and Safety, Advocacy and Social Justice, and Research and Education.	2022	General	Hospital and community	All	Formation of a Neighbourhood Council.	Led by senior manager. Employ community consultant for each core area of focus and a village of community health workers. The Neighbourhood Council will consist of CHOP employees who live in the West Philadelphia area.	Has website and repository of resources for professionals.	Not reported.

For peer review only

Table 1. Detailed summary of included approaches

In-text ref	Country	Hospital	Approach	Type	Details	Year	Inequity	Setting	Population	Outcomes reported	Staff/ resources involved	Other useful learning	Evaluation/ measure of success
49	USA	Children's Hospital of Philadelphia (CHOP)	Established research centre/ portfolio	Meso	'Policy Lab': A research portfolio investigating how policies and programs can improve the health of historically marginalized children and adolescents. Advancing health equity through a research lens. PolicyLab is a Center of Emphasis within Children's Hospital of Philadelphia's Research Institute	2022	General	Online, hospital, community	Minority infants and mothers, LGBTQ children, immigrant families, low-income families	Research articles and multiple projects including a brachial plexus recruitment study. Before the health equity intervention, 54% of diverse families and 35% of white families agreed to participate in the study. After implementing the recommended strategies, 68% of diverse families and 71% of white families enrolled in the study.	Team includes 35 faculty and more than 60 staff who are experts in medicine, public health, social work, psychology, law, biostatistics, health services research and population health	Database/ repository of projects and publications. Inclusive recruitment strategies help recruit patients into trials, so that the study population is more representative of the real population. Simple modifications are required to completely change the outcome of recruitment – "it's simple things that make a difference."	Brachial plexus study - the % of diverse and % of white families that were invited to participate in the research that decided to take part (changes pre to post study).
50	USA	Cincinnati Children's	Established department / centre	Meso	The Center for Child Equity builds equitable care capabilities among staff through training, consultation and partnerships with research and clinical divisions, diversity and community relations teams, as well as a broad coalition of families and other external stakeholders.	2021	General	Hospital and community	All	None reported.	Hospital, community and social care partners	N/A	Not reported.
51	USA	Seattle Children's Hospital	Established department / centre	Meso	Center for Diversity and Health Equity (CDHE). Includes patient and family education programme, interpreter services, cultural navigation, family resource centre (kitchen, showers, computers, laundry, TV, Wi-Fi, parent support, phone chargers etc.), guest services, spiritual care, resources for transgender patients.<br>Health Equity Research Grants Program: to fund project to advance work around health equity, have Education and resources for healthcare professionals on equity, and a programme around human trafficking.	2021	General	Hospital	All	Programme of work around human trafficking including video workshops and video. Training for staff around health equity.	Diversity and Health Equity team: 14 people, work closely with the Odessa Brown Children's Clinic, Seattle Children's Research Institute and many other groups within Seattle Children's	Created a self-assessment toolkit for staff to assess performance towards health equity.	Not reported.
52	USA	Children's Hospital of Philadelphia (CHOP)	Partnership Initiative	Meso	To target the social determinants of health. Healthier Together partners with community groups, nonprofits and government agencies to learn from each other and multiply the impact of the work. Residents and community organisations participate in oversight/governance meetings, and outreach for program development. Advisers include experts from across CHOP, residents from West and Southwest Philadelphia, and representatives from the School District of Philadelphia, Philadelphia Department of Public Health and other services.	2019	Social determinants of health	Hospital and community	Children in three zip codes in West and Southwest Philadelphia	$10 million invested in the community. 13,450 individuals served with 21 partners and collaborators. $3,840 back to families on average through financial counselling and tax prep. 136 homes renovated to reduce asthma triggers. 45,570+ healthy meals distributed to families	Hospital and partners	Purchase goods and services from local companies that value a diverse workforce and provide meaningful work opportunities to underrepresented members of our community.<br>Healthier Together partners with community groups, nonprofits and government agencies to learn from each other and multiply the impact of the work. Residents and community organisations participate in oversight/governance meetings, and outreach for program development.	$ invested in community, individuals served, homes renovated, meals distributed.
53	Canada	Holland Bloorview Kids Rehabilitation Hospital, Toronto	Screening tool	Meso	Created a social needs screening tool, used during appointments by trained clinicians to identify the social needs and barriers of clients and their families. Family Navigation Hub was created: a resource that offers one-on-one consultations and services as well as monthly virtual workshops in partnership with external organizations, known as the CommunityCONNECT series, for families to access and to help close the gaps when it comes to housing, food, childcare, respite and community connection. Developed a partnership with FoodShare to look at food security needs.	2023	Social determinants of health	Hospital and community	Children with a disability	Community Kitchens Series from FoodShare Toronto: families who register are sent ingredients to prepare culturally diverse recipes. They prepare the meals together with other families while on Zoom, Housing support, social needs screening tool for clinicians.	Screened by clinicians, if eligible family support specialists work with families	The FoodShare program has brought families together and provided a sense of community.	Qualitative feedback from parents.

For peer review only

Table 1. Detailed summary of included approaches

In-text ref	Country	Hospital	Approach	Type	Details	Year	Inequity	Setting	Population	Outcomes reported	Staff/ resources involved	Other useful learning	Evaluation/ measure of success
54	Australia	Queensland Childrens Hospital, Brisbane	Service	Meso	Indigenous liaison service - to support, advocate, provide assistance to indigenous people accessing health and social services.	2020	Outcomes	Hospital	Children and families from Aboriginal and Torres Strait Islander communities	None reported.	Indigenous liaison officers	N/A	Not reported
55	USA	Boston Children's Hospital	Advocacy	Micro	Analysed data on impact of Covid-19 on communities of colour. Raised awareness on structural racism and differences in outcomes. Communication through Twitter.	2021	Outcomes	Online	Communities of colour	The posts reached more than 100,000 people, with reposts by hospitals and several state legislators.	Group of paediatricians	Analysing data showed that rates of Covid-19 infection, serious illness, and death were far higher in Black populations throughout the country. Recognized that data can play an important role in driving more equitable decisions and policies. Social media can be a useful medium to spread awareness, but it has its limitations as well.	Number of people reached on Twitter, number of reposts.
56	Canada	Hospital for Sick Children Toronto (SickKids)	Screening tool	Micro	Developed a paediatric data tool and health equity survey. Also provided training for staff.	2019	General	Hospital	All	Increased focus on equity. Better data collection. Staff trained.	Clerical staff collected data	Registration is an optimal point in the healthcare visit to collect sociodemographic information. Patients and caregivers willing to provide information.	Satisfaction of parents in giving information. Number and percentage of tools completed. Number of staff trained.
57	Canada	British Columbia Children's Hospital Vancouver	Service	Micro	Primary healthcare outreach clinics for children, youth, and families in Vancouver's inner-city neighbourhoods. the RICHER program seeks to address health equity disparities and particularly disparities in health care access that many marginalized and vulnerable children and families experience. The model recognizes the importance of trusted relationships and seeks to increase capacity and resilience for the families served.	2023	Access	Community	Inner city families	A wide range of community outreach services at a wide range of centres.	Nurse Practitioners, general paediatricians, development paediatricians, Adolescent Health and Medicine doctors, Child and Adolescent Psychiatry physicians, dermatology doctor, research manager	N/A	Not reported.
58	Australia	Sydney Children's Hospital Network	Intervention	Micro	Two new projects - the RuralKidsGPS project and PEACH project. Aim to improve equity and access to healthcare services. RuralKids will trial integrated care in rural districts and PEACH will establish technological solutions and sustainable systems for complex needs.	2021	Access	Community	Rural populations and socioeconomically disadvantaged communities	RuralKidsGPS: Paediatric care co-ordination has already shown promising results in metropolitan Sydney. Over a two-year period, it demonstrated a 40 per cent reduction in emergency department presentations and a 42 per cent decrease in day-only admissions – saving more than 50,000km of travel for families.	Care coordinators will work closely with local care teams and families to create shared care plans, support technology-enabled healthcare, and connect families with local services to meet the child's needs.	N/A	The success of the RuralKidsGPS and PEACH will be evaluated in the next few years with the intention to roll out both programs more widely across the state.
59	USA	Boston Children's Hospital	Intervention	Micro	Case management and home visits by a community health worker for children from low-income families of colour with asthma.	2023	Outcomes	Community	Children with asthma from low-income families of colour	By 2022, there were more than 2,491 patients who enrolled in the Community Asthma Initiative. Their involvement led to: 82% reduction in the percentage of patients with asthma-related hospitalization, 55% reduction in the percentage of patients with Emergency Department visits, 45% reduction in the percentage of patients with missed school days for children, 55% reduction in the percentage of patients with lost work days for parents.	Community health workers	Beneficial to wider health of children involved.	Changes in the percentage (%) of patients with asthma-related hospitalization, % of patients with Emergency Department visits, % of patients with missed school days for children, % of patients with lost workdays for parents.

For peer review only

Appendix I: Search terms

Database	Search	Terms
Overton	1	(child OR children OR paediatric OR pediatric OR "young people") AND (hospital) AND ("health inequality" OR "health disparity" OR "health equity")
	2	title: equity child
	3	title: inequality child
	4	title: children hospital equity
	5	title: addressing inequalities
	6	title: addressing inequity
Open Grey	1	Health inequality
	2	Health equity
	3	Health Disparity
Openmd.com	1	(child OR children OR paediatric OR pediatric OR "young people") AND (hospital) AND ("health inequality" OR "health disparity" OR "health equity")
Tripdatabase	1	title: inequalities children hospital
	2	title: address health equity
	3	addressing child health inequality hospital
Duckduckgo	1	hospital based approaches to addressing health inequalities
	2	how do childrens hospitals reduce inequalities
	3	(child OR children OR paediatric OR pediatric OR "young people") AND (hospital) AND ("health inequality" OR "health disparity" OR "health equity")
Google	1	(child OR children OR paediatric OR pediatric OR "young people") AND (hospital) AND ("health inequality" OR "health disparity" OR "health equity")
	2	how do childrens hospitals reduce inequalities
	3	childrens hospital approach to inequalities
	4	Childrens hospital strategy to reduce health inequalities
APHA	1	Childrens hospital
	2	child health inequality
	3	child equity
	4	child health disparity
WHO	1	children's hospital
	2	health inequality
	3	child health equity
	4	health equity
	5	health disparity
NHS England	1	childrens hospital
	2	health inequality
	3	health equity
	4	health disparity
	5	health inequality
OHID	1	"children's hospital"
	2	"child health inequalities"
	3	"health inequalities" AND "hospital"
HMIC	1	“inequalities”
Hospital websites	All	Inequal* OR inequity OR disparit*

For peer review only

Appendix II: Data extraction form

**Study details and characteristics**	
Reference/ weblink	
Hospital name	
Country of study	
Year published/ website updated	
Population and sample size	
Context (healthcare system)	
Publication type (e.g. report/ website/ news article	
Stand alone hospital / part of network	
**Details of the approach**	
Type of approach (e.g. strategy/ centre..)	
Aim of the approach	
Inequality in focus	
Population in focus	
Setting	
Length of intervention?	
Staff	
**Results extracted from study** (in relation to the concept of the scoping review)	
Main Outcomes/ Results	
Additional outcomes/ any differential effects	
Key findings/ Conclusions	
How were effects measured?	
Other notes	

For peer review only

Preferred Reporting Items for Systematic reviews and Meta-Analyses extension for Scoping Reviews (PRISMA-ScR) Checklist

SECTION	ITEM	PRISMA-ScR CHECKLIST ITEM	REPORTED ON PAGE #
**TITLE**			
Title	1	Identify the report as a scoping review.	1
**ABSTRACT**			
Structured summary	2	Provide a structured summary that includes (as applicable): background, objectives, eligibility criteria, sources of evidence, charting methods, results, and conclusions that relate to the review questions and objectives.	2
**INTRODUCTION**			
Rationale	3	Describe the rationale for the review in the context of what is already known. Explain why the review questions/objectives lend themselves to a scoping review approach.	4
Objectives	4	Provide an explicit statement of the questions and objectives being addressed with reference to their key elements (e.g., population or participants, concepts, and context) or other relevant key elements used to conceptualize the review questions and/or objectives.	4
**METHODS**			
Protocol and registration	5	Indicate whether a review protocol exists; state if and where it can be accessed (e.g., a Web address); and if available, provide registration information, including the registration number.	5
Eligibility criteria	6	Specify characteristics of the sources of evidence used as eligibility criteria (e.g., years considered, language, and publication status), and provide a rationale.	5
Information sources*	7	Describe all information sources in the search (e.g., databases with dates of coverage and contact with authors to identify additional sources), as well as the date the most recent search was executed.	5
Search	8	Present the full electronic search strategy for at least 1 database, including any limits used, such that it could be repeated.	17
Selection of sources of evidence†	9	State the process for selecting sources of evidence (i.e., screening and eligibility) included in the scoping review.	5
Data charting process‡	10	Describe the methods of charting data from the included sources of evidence (e.g., calibrated forms or forms that have been tested by the team before their use, and whether data charting was done independently or in duplicate) and any processes for obtaining and confirming data from investigators.	5
Data items	11	List and define all variables for which data were sought and any assumptions and simplifications made.	6
Critical appraisal of individual sources of evidence§	12	If done, provide a rationale for conducting a critical appraisal of included sources of evidence; describe the methods used and how this information was used in any data synthesis (if appropriate).	N/A

For peer review only - http://bmjopen.bmj.com/site/about/guidelines.xhtml

SECTION	ITEM	PRISMA-ScR CHECKLIST ITEM	REPORTED ON PAGE #
Synthesis of results	13	Describe the methods of handling and summarizing the data that were charted.	6
**RESULTS**			
Selection of sources of evidence	14	Give numbers of sources of evidence screened, assessed for eligibility, and included in the review, with reasons for exclusions at each stage, ideally using a flow diagram.	6
Characteristics of sources of evidence	15	For each source of evidence, present characteristics for which data were charted and provide the citations.	Table 1
Critical appraisal within sources of evidence	16	If done, present data on critical appraisal of included sources of evidence (see item 12).	N/A
Results of individual sources of evidence	17	For each included source of evidence, present the relevant data that were charted that relate to the review questions and objectives.	Table 1
Synthesis of results	18	Summarize and/or present the charting results as they relate to the review questions and objectives.	Table 1
**DISCUSSION**			
Summary of evidence	19	Summarize the main results (including an overview of concepts, themes, and types of evidence available), link to the review questions and objectives, and consider the relevance to key groups.	9
Limitations	20	Discuss the limitations of the scoping review process.	10
Conclusions	21	Provide a general interpretation of the results with respect to the review questions and objectives, as well as potential implications and/or next steps.	10
**FUNDING**			
Funding	22	Describe sources of funding for the included sources of evidence, as well as sources of funding for the scoping review. Describe the role of the funders of the scoping review.	11

JBI = Joanna Briggs Institute; PRISMA-ScR = Preferred Reporting Items for Systematic reviews and Meta-Analyses extension for Scoping Reviews.

* Where *sources of evidence* (see second footnote) are compiled from, such as bibliographic databases, social media platforms, and Web sites.

† A more inclusive/heterogeneous term used to account for the different types of evidence or data sources (e.g., quantitative and/or qualitative research, expert opinion, and policy documents) that may be eligible in a scoping review as opposed to only studies. This is not to be confused with *information sources* (see first footnote).

‡ The frameworks by Arksey and O'Malley (6) and Levac and colleagues (7) and the JBI guidance (4, 5) refer to the process of data extraction in a scoping review as data charting.

§ The process of systematically examining research evidence to assess its validity, results, and relevance before using it to inform a decision. This term is used for items 12 and 19 instead of "risk of bias" (which is more applicable to systematic reviews of interventions) to include and acknowledge the various sources of evidence that may be used in a scoping review (e.g., quantitative and/or qualitative research, expert opinion, and policy document).

From: Tricco AC, Lillie E, Zarin W, O'Brien KK, Colquhoun H, Levac D, et al. PRISMA Extension for Scoping Reviews (PRISMAScR): Checklist and Explanation. Ann Intern Med. 2018;169:467–473. doi: 10.7326/M18-0850.

For peer review only - http://bmjopen.bmj.com/site/about/guidelines.xhtml
